# Bidirectional Endothelial Feedback Drives Turing-Vascular Patterning and Drug-Resistance Niches: A Hybrid PDE-Agent-Based Study

**DOI:** 10.3390/bioengineering12101097

**Published:** 2025-10-12

**Authors:** Zonghao Liu, Louis Shuo Wang, Jiguang Yu, Jilin Zhang, Erica Martel, Shijia Li

**Affiliations:** 1 Innovation Center for Cancer Research, Clinical Oncology School, Fujian Medical University, Fuzhou 350014, China; liuzonghao@fjmu.edu.cn; 2 Department of Mathematics, University of Tennessee, Knoxville, TN 37996, USA; swang116@vols.utk.edu; 3 College of Engineering, Boston University, Boston, MA 02215, USA; 4 Department of Mathematics, University College London, London WC1E 6BT, UK; 5 Department of Mathematics, Imperial College London, London SW7 2AZ, UK; jilin.zhang25@imperial.ac.uk; 6 Department of Internal Medicine, Yale School of Medicine, Bridgeport Hospital, Bridgeport, CT 06610, USA; erica.martel@bpthosp.org

**Keywords:** reaction-diffusion equations, Turing instability, angiogenesis, hybrid PDE-agent-based model, endothelial-sourced angiogenic feedback, chemotaxis, pharmacokinetics, perfusion heterogeneity, tumor drug resistance, tumor microenvironment

## Abstract

We present a hybrid partial differential equation-agent-based model (PDE-ABM). In our framework, tumor cells secrete tumor angiogenic factor (TAF), while endothelial cells chemotactically migrate and branch in response. Reaction–diffusion PDEs for TAF, oxygen, and cytotoxic drug are coupled to discrete stochastic dynamics of tumor cells and endothelial tip cells, ensuring multiscale integration. Motivated by observed perfusion heterogeneity in tumors and its pharmacokinetic consequences, we conduct a linear stability analysis for a reduced endothelial–TAF reaction–diffusion subsystem and derive an explicit finite-domain threshold for Turing instability. We demonstrate that bidirectional coupling, where endothelial cells both chemotactically migrate along TAF gradients and secrete TAF, is necessary and sufficient to generate spatially periodic vascular clusters and inter-cluster hypoxic regions. These emergent patterns produce heterogeneous drug penetration and resistant niches. Our results identify TAF clearance, chemotactic sensitivity, and endothelial motility as effective levers to homogenize perfusion. The model is two-dimensional and employs simplified kinetics, and we outline necessary extensions to three dimensions and saturable kinetics required for quantitative calibration. The study links reaction–diffusion mechanisms with clinical principles and suggests actionable strategies to mitigate resistance by targeting endothelial–TAF feedback.

## 1. Introduction

Tumors actively remodel their blood supply. When endothelial cells migrate toward chemical cues secreted by tumors, they generate alternating regions of high and low perfusion. These spatial patterns create treatment-resistant niches where drugs penetrate poorly and hypoxia persists. We develop a multiscale model that couples chemical diffusion of oxygen, drug, and signaling molecules with individual tumor and endothelial cells. The model identifies when and how vascular patterns emerge and how parameter changes can restore uniform perfusion and improve therapy.

Drug resistance remains a major obstacle to successful cancer treatment [1]. It results not only from genetic and epigenetic alterations within tumor cells but also from spatial heterogeneity in the tumor microenvironment [2,3,4,5,6]. Hypoxic and irregular drug diffusion create protective refugia that allow resistant clones to survive and expand [7,8,9]. Understanding how such microenvironmental heterogeneity forms is essential for designing therapies that reach and eliminate all tumor regions.

Endothelial cell migration drives much of this heterogeneity. Endothelial cells line blood vessels and generate new vascular sprouts during angiogenesis. They are not malignant but belong to the host vasculature that tumors recruit to supply oxygen and nutrients. These cells sense and move along gradients of vascular endothelial growth factor (VEGF), a signaling molecule released by hypoxic tumor cells [10]. This process, known as angiogenesis, reorganizes the vascular network and alters the spatial distribution of oxygen and drugs. Where sprouts connect, perfusion improves; where they fail, hypoxia and poor drug delivery persist. Clinically, such disorganized and uneven vasculature correlates strongly with metastasis, therapeutic failure, and relapse. Therefore, understanding how endothelial cells migrate and self-organize holds both scientific and translational importance: it provides insight into tumor progression and guides strategies to normalize vessels and homogenize treatment delivery.

Tumor cells also change dynamically within this evolving microenvironment. They divide, migrate, die, and adapt to chemical and mechanical cues generated by the vasculature. The feedback between vascular remodeling and tumor evolution links microenvironmental architecture with resistance and metastasis [11]. Vascular geometry shapes selective pressures that promote resistant phenotypes, while tumor-derived signals redirect vessel growth. This reciprocal coupling drives both spatial heterogeneity and therapeutic failure.

Mathematical modeling provides a rigorous framework by analyzing these multiscale feedback [12,13]. In mathematical oncology, three main paradigms describe tumor–vascular dynamics: continuum partial differential equation (PDE) models, agent-based models (ABMs), and hybrid PDE-ABM frameworks. Classical continuum models represent angiogenesis at the tissue scale by coupling cell density with diffusion factors [14]. The Keller–Segel formulation captures endothelial migration up VEGF gradients and predicts the conditions under which vascular patterns form [15]. These models provide analytic insight but treat cells as continuous densities and cannot represent stochastic, discrete cell behaviors.

ABMs address this limitation by explicitly simulating individual cells that divide, migrate, and interact with local microenvironments according to probabilistic rules [16]. They describe clonal diversity and drug response with high fidelity but become computationally expensive at the tissue scale. Hybrid PDE-ABM models combine the advantages of both approaches by linking reaction–diffusion equations for chemical transport with discrete cellular agents [17,18,19]. This hybridization links microscale cell behavior to macroscale spatial organization and has become a powerful tool for investigating resistance mechanisms and therapy optimization [12,13,20,21]. Appendix A summarizes representative models and highlights those that include angiogenesis, resistance, or spatial heterogeneity.

Despite significant progress, most existing angiogenesis models assume unidirectional coupling: tumor cells secrete VEGF, endothelial cells migrate up its gradient, but endothelial cells do not regulate VEGF production. In reality, endothelial cells can also modulate VEGF production, creating a feedback loop that amplifies local signaling [22]. Such bidirectional coupling may destabilize homogeneous tissue states and generate self-organized vascular patterns akin to those predicted by Turing’s reaction–diffusion theory [23].

We construct a hybrid PDE-ABM model that integrates stochastic tumor and endothelial tip dynamics with reaction–diffusion equations for oxygen, drug, and tumor angiogenic factor (TAF). The model compares unidirectional and bidirectional coupling. Analytical and numerical analyses show that bidirectional coupling is necessary and sufficient for Turing-type instabilities that generate spatially periodic vascular clusters separated by hypoxic regions. These emergent patterns explain the coexistence of vascularized and hypoxic tumor zones and demonstrate how vascular adaptation fosters drug resistance. The analysis identifies three quantitative control points—TAF clearance, chemotactic sensitivity, and endothelial motility—that can be tuned to homogenize perfusion and improve therapeutic delivery.

Numerically, we implement an alternating direction implicit (ADI) scheme for the PDE components [24] and integrate stochastic agent-based updates for cellular events to ensure stability and efficiency.

The biological implications extend beyond oncology. Controlled vascular patterning also governs success in regenerative medicine and tissue engineering. The same bidirectional endothelial–TAF coupling that drives Turing-type patterns in tumors also provides principles for optimizing scaffold geometry, growth factor delivery, and perfusion to achieve uniform oxygenation and drug accessibility.

**Figure 1:**
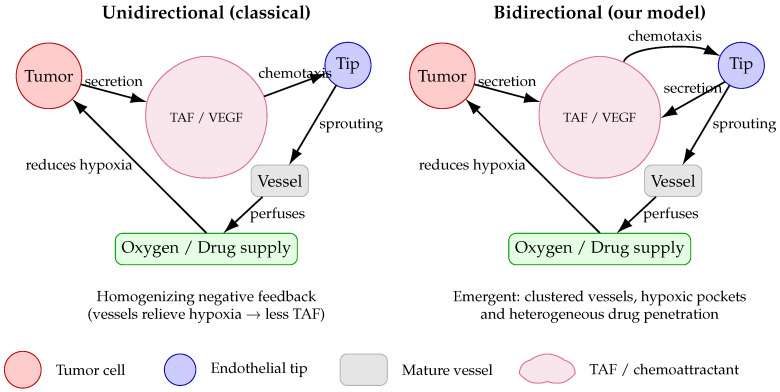
Two-panel schematic. (**Left**) Classical unidirectional picture — hypoxic tumor cells secrete TAF, endothelial tips chemotax, vessels form and supply oxygen/drug, which tends to reduce TAF (stabilizing feedback). (**Right**) Bidirectional picture — endothelial tips also secrete/amplify TAF (autocrine/paracrine feedback), creating a self-reinforcing loop that can produce clustered vascular patterns and hypoxic/drug-poor niches. The panels emphasize two-way coupling between TAF and discrete tips and vessel cells. The green box indicates the microenvironmental field (oxygen or drug supply) mediating feedback to the tumor and vasculature.

**Figure 2:**
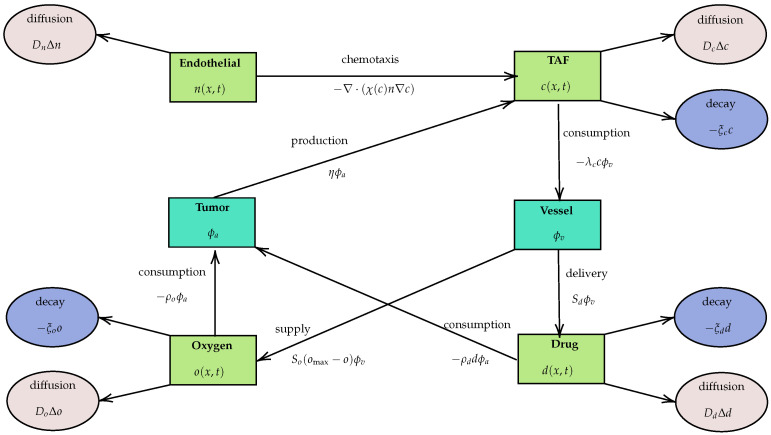
Schematic diagram of the modeled processes and state variables. The four continuum densities—endothelial cells n(x,t), angiogenic factor c(x,t), oxygen o(x,t), and drug d(x,t)—are shown with their diffusion, decay, production, consumption, and delivery terms, together with discrete tumor $\phi_{a}$ and vessel cells $\phi_{v}$. Each labeled arrow corresponds to a process term in the PDE system (e.g., $D_{i}\Delta i$, $-\xi_{i}i$, $S_{i}\phi_{v}$, $-\rho_{i}i\phi_{a}$, −∇·(χ(c)n∇c). This schematic clarifies the meaning of all terms before the equations are introduced in the text.

For clarity, Figure 1 contrasts the classical unidirectional and our bidirectional frameworks, and Figure 2 summarizes the PDE components for the four scalar state variables (n,c,d,o). Section 2 presents the full model, Section 3 details parameter estimation and numerical schemes, Section 4 provides the linear stability analysis, and Section 5 reports numerical simulations, bifurcation behaviors, and Turing patterns. Section 6 concludes with biological and computational implications.

## 2. Methods

We organize Methods into two parts: PDE equations in Section 2.1 and agent-based rules in Section 2.2.

### 2.1. PDE Equations

We model tumor–vasculature interactions using four scalar state variables that depend on space and time: endothelial cell density n(x,t), tumor angiogenic factor (TAF or VEGF) concentration c(x,t), drug concentration d(x,t), and oxygen concentration o(x,t). Throughout, the spatial variable $x=\left(x_{1},x_{2}\right)\in U\subset R^{2}$ represents position in a two-dimensional tissue domain. The same formulation extends directly to three dimensions with $x=(x_{1},x_{2},x_{3})$. Endothelial cells are normal vessel-lining cells; their density is *n*. Tumor cells are modeled as discrete cells (agents) and are denoted by $a\in \Lambda_{t}$ at time *t*. The growing vasculature is represented by vessel sites (endothelial tips/segments) $v\in V_{t}$ at time *t*, and these vessel sites can deliver oxygen and drugs. For clarity, $\Lambda_{t}$ and $V_{t}$ denote the sets of tumor cells and vessel sites at time *t*, respectively.

We further classify tumor cells by local oxygen into normoxic (sufficient oxygen) and hypoxic (reduced oxygen) subsets, written $\Lambda_{t}^{n}$ and $\Lambda_{t}^{h}$ at time *t*. Hypoxia is defined by thresholds on o(x,t) (normoxic if $o>o_{\mathrm{hyp}}$; hypoxic if $o_{\mathrm{apop}}<o\leq o_{\mathrm{hyp}}$), and it drives angiogenic signaling: hypoxic tumor cells secrete TAF, which appears as a source in the *c*-equation, while endothelial cells bias their motion up ∇c (chemotaxis) with sensitivity χ(c), which enters the *n*-equation through the flux term ∇·(χ(c)n∇c). Drugs and oxygen obey transport with vascular supply and tumor uptake. Below, we write the main PDEs for the four scalar variables n,c,d,o and, after each, give a brief biological meaning and how the discrete tumor cells $\Lambda_{t}$ and vessel sites $V_{t}$ act as sources or sinks.

(i) Endothelial dynamics *n*

Endothelial cell movement has two components. The first is random motility, the undirected cell motion that smooths local density and is modeled by the diffusion term $D_{n}\Delta n$. The second is chemotaxis, directed migration up the gradient of TAF/VEGF concentration c(x,t) and is modeled by the drift term −∇·(χ(c)n∇c). Together, these processes satisfy the standard Keller–Segel conservation law [15]

(1) $$\begin{array}{cc} \; & \partial_{t}n=D_{n}\Delta n-\nabla \cdot \left(\chi (c)n\nabla c\right),\\ \; & \chi \left(c\right)=\frac{\chi_{0}k_{1}}{k_{1}+c},\\ \; & \chi \left(c\right)\to \chi_{0}\;\mathrm{as}\;c\to 0,\\ \; & \chi (c)\to 0\;\mathrm{as}\;c\to \infty . \end{array}$$

Here, $D_{n}$ is the endothelial cell diffusion coefficient, and χ(c) is the chemotactic sensitivity. The flux of the endothelial cells is

$$\begin{array}{c} J_{n}=-D_{n}\nabla n+\chi \left(c\right)n\nabla c. \end{array}$$

The first term is random motility, and the second is drift up the TAF gradient. The decreasing, saturating function χ(c) reflects receptor kinetics: if endothelial receptors bind ligand with occupancy $f\left(c\right)=c/\left(k_{1}+c\right)$, then gradient sensing depends on ∇f and diminishes as receptors saturate, producing reduced responsiveness at high *c*. The chosen form $\chi \left(c\right)=\chi_{0}k_{1}/\left(k_{1}+c\right)$ is a simple monotone approximation that captures this biophysical effect [25,26]. Here, $\chi_{0}$ is the maximal chemotactic coefficient and $k_{1}>0$ modulates TAF sensitivity. The companion equation for *c* (stated below) makes its kinetics explicit by including diffusion, natural clearance, secretion by hypoxic tumor cells, and endothelial uptake; production of chemoattractant therefore appears there rather than in Equation (1).

(ii) TAF dynamics *c*

The TAF concentration c(x,t) evolves through diffusion, clearance, secretion by hypoxic tumor cells, and uptake by vessels:

(2) $$\begin{array}{c} \partial_{t}c=D_{c}\Delta c-\xi_{c}c+\eta \sum_{a\in \Lambda_{t}^{h}}\phi_{a}-\lambda c\sum_{v\in V_{t}}\phi_{v}. \end{array}$$

Here, $D_{c}$ is the diffusion coefficient, $\xi_{c}$ the clearance rate, η the secretion rate per hypoxic cell, and λ the uptake rate near vessels. Tumor cells $a\in \Lambda_{t}$ and vessel sites $v\in V_{t}$ occupy positions $a^{x}\left(t\right),v^{x}\left(t\right)\in U$. We emphasize that the positions $a^{x}\left(t\right),v^{x}\left(t\right)$ are not input parameters but dynamical states that enter Equation (2) as moving sources and sinks. Each influences nearby tissue through a normalized spatial kernel:

$$\begin{array}{c} \phi_{a}\left(x,t\right)=\left\{\begin{array}{cc} \frac{1}{\pi R_{c}^{2}} & if∥x-a^{x}\left(t\right)∥\leq R_{c},\\ 0 & \mathrm{otherwise}, \end{array}\right.\\ \phi_{v}\left(x,t\right)=\left\{\begin{array}{cc} \frac{1}{\pi R_{c}^{2}} & if∥x-v^{x}\left(t\right)∥\leq R_{c},\\ 0 & \mathrm{otherwise}. \end{array}\right. \end{array}$$

The normalization ensures $\int_{U}\phi_{a}\left(x,t\right)\;dx=\int_{U}\phi_{v}\left(x,t\right)\;dx=1$. The parameter $R_{c}>0$ defines the cell radius.

Biological interpretation. The diffusive term $D_{c}\Delta c$ captures the passive spread of angiogenic factors in tissue. The term $-\xi_{c}c$ models natural clearance or degradation. The localized source $\eta \sum_{a\in \Lambda_{t}^{h}}\phi_{a}$ adds TAF in regions surrounding hypoxic tumor cells, while the sink term $-\lambda c\sum_{v\in V_{t}}\phi_{v}$ removes TAF near endothelial vessels that absorb it. Together, these processes establish angiogenic gradients that drive the directed migration of endothelial cells in Equation (1), coupling tumor hypoxia to vascular response.

(iii) Drug dynamics (*d*)

Drug concentration d(x,t) changes by diffusion, clearance, uptake by tumor cells, and vascular delivery:

(3) $$\begin{array}{c} \partial_{t}d=D_{d}\Delta d-\xi_{d}d-\rho_{d}d\sum_{a\in \Lambda_{t}}\phi_{a}+S_{d}\left(t\right)\sum_{v\in V_{t}}\phi_{v}. \end{array}$$

Here, $D_{d}$ is the drug diffusion coefficient (spatial dispersal), $\xi_{d}$ is systematic/interstitial clearance, $\rho_{d}$ is tumor uptake per cell (a sink proportional to local *d* and the nearby cell density), and $S_{d}\left(t\right)$ is the vascular delivery at vessel sites. We encode dosing regimens via

$$\begin{array}{c} S_{d}\left(t\right)=\left\{\begin{array}{cc} S_{d}, & \mathrm{treatment}\;\mathrm{on},\\ 0, & \mathrm{treatment}\;\mathrm{off}, \end{array}\right. \end{array}$$

for pulsed therapy, and $S_{d}\left(t\right)\equiv S_{d}$ for continuous low dose administration.

Biological interpretation. The term $D_{d}\Delta d$ governs spatial dispersal of drug. The term $-\xi_{d}d$ captures clearance. The sink $-\rho_{d}d\sum_{a\in \Lambda_{t}}\phi_{a}$ models tumor uptake. The source $S_{d}\left(t\right)\sum_{v\in V_{t}}\phi_{v}$ represents time dependent vascular delivery. Collectively, Equation (3) captures the spatiotemporal distribution of therapeutic agents under distinct dosing regimens.

(iv) Oxygen dynamics (*o*)

We represent oxygen with diffusion, decay, tumor consumption, and feedback-limited vascular supply:

(4) $$\begin{array}{c} \partial_{t}o=D_{o}\Delta o-\xi_{o}o-\rho_{o}\sum_{a\in \Lambda_{t}}\phi_{a}+S_{o}\left(o_{\max}-o\right)\sum_{v\in V_{t}}\phi_{v}. \end{array}$$

Here, $D_{o}$ denotes oxygen diffusion coefficient, $\xi_{o}$ denotes natural degradation, $\rho_{o}$ denotes tumor uptake, $S_{o}$ denotes vessel supply rate, and $o_{\max}$ denotes vessel saturation concentration. Therefore, the term $S_{o}\left(o_{\max}-o\right)$ implies that if local oxygen *o* is near $o_{\max}$, the source from vessels is small.

Biological interpretation. The term $D_{o}\Delta o$ models passive oxygen spread. The term $-\xi_{o}o$ captures oxygen decay. The term $-\rho_{o}\sum_{a\in \Lambda_{t}}\phi_{a}$ captures cellular consumption. The feedback-controlled source term $S_{o}\left(o_{\max}-o\right)\sum_{v\in V_{t}}\phi_{v}$ ensures oxygen saturates near physiological levels. This formulation captures hypoxia in poorly vascularized regions and homeostatic oxygen regulation in well-perfused zones.

We replace linear uptake by Michaelis–Menten saturation kinetics when data justify nonlinear consumption [27]. This formulation reduces to linear uptake at low concentrations while preventing unbounded consumption at high concentrations. For drug and oxygen consumption, we write

$$\begin{array}{c} \frac{\rho_{d}d}{K_{d}+d}\sum_{a\in \Lambda_{t}}\phi_{a},\;\frac{\rho_{o}o}{K_{o}+o}\sum_{a\in \Lambda_{t}}\phi_{a}. \end{array}$$

Here, $\rho_{d},\rho_{o}$ denote maximum consumption rates and $K_{d},K_{o}$ denote half-saturation concentrations. For $d\ll K_{d},o\ll K_{o}$, the kinetics reduce to the linear form $\frac{\rho_{d}d}{K_{d}+d}\approx \frac{\rho }{K_{d}}d,\frac{\rho_{o}o}{K_{o}+o}\approx \frac{\rho_{o}}{K_{o}}o$. In contrast, consumption saturates at $\rho_{d},\rho_{o}$ when concentrations are large. See Ojwang et al. [28] and Ginneken et al. [29] for Michaelis–Menten kinetics of tumor oxygenation and pharmacokinetics, respectively.

Our drug equation includes diffusion, uptake, and decay, but not efflux, an active resistance mechanism in which tumor cells pump drug back into the extracellular space. This omission is deliberate for tractability, though Appendix B illustrates how efflux can be incorporated via a source term proportional to the tumor cell number.

Given the limited availability of reliable $K_{m}$ values for our system, we adopt the linear form as a parsimonious approximation within the biologically relevant range. To further confirm that this simplification does not alter our conclusions, we performed a parallel linear stability analysis analogous to the comprehensive analysis in Section 4. We replace the oxygen supply term $S_{o}\left(o_{\max}-o\right)\sum_{v}\phi_{v}$ by Michaelis–Menten kinetics. Appendix G provides a detailed derivation and demonstrates that the qualitative stability results are robust: all perturbation modes remain stable without any spurious pattern-forming instabilities.

Homogeneous Neumann boundary conditions approximate conservation absent sources in the isolated tissue domain:

$$\begin{array}{c} \vec{n}\cdot \nabla \phi {|}_{\partial U}=0,\;\phi \in \left\{n,c,d,o\right\}. \end{array}$$

Here, $\vec{n}$ is the unit outward normal on the boundary ∂U. Real tumor microenvironment and tissue may receive external signals, violating the Neumann boundary condition. Dirichlet or Robin boundary conditions could be choices, and we leave such an extension for future work. We discretize the chemotaxis Equation (1) with an explicit time-stepping scheme and solve the remaining reaction–diffusion equations with an ADI scheme for numerical efficiency and stability.

Table 1 summarizes the mechanisms encoded in each PDE. We model the system on a two-dimensional tissue slice of effective thickness $h=1\times 10^{-4}$ m, representative of thin in vitro assays [30]. We convert dimensional parameters reported in standard volumetric (3D) units as in the literature to the 2D formulation using this thickness. Appendix E gives the conversion details.

We nondimensionalize using characteristic length scale *L*, diffusion time $\tau =L^{2}/D$, and concentration scales $n_{0},c_{0},d_{0},o_{\max}$. Table 2 lists these characteristic scales. Here, *D* denotes a representative diffusion coefficient, chosen such that $\tau =L^{2}/D=5.76\times 10^{4}$ s for a spatial scale $L=5\times 10^{-3}$ m. All parameter values are taken from the authoritative set in Table 3; see also the machine-readable files params.json and params.csv in the GitHub Repository in the Data Availability Statement Section.

The full nondimensionalization details are (Note that the nondimensional form reparameterizes the saturation as $\alpha =c_{0}/k_{1}$)

(5) $$\begin{array}{cc} \; & {\tilde{D}}_{n}=\frac{D_{n}}{D},\;{\tilde{\chi }}_{0}=\frac{\chi_{0}c_{0}}{D},\;\alpha =\frac{c_{0}}{k_{1}},\\ \; & {\tilde{D}}_{c}=\frac{D_{c}}{D},\;{\tilde{\xi }}_{c}=\tau \xi_{c},\;\tilde{\eta }=\frac{\eta \tau n_{0}}{c_{0}},\;\tilde{\lambda }=\lambda \tau n_{0},\\ \; & {\tilde{D}}_{d}=\frac{D_{d}}{D},\;{\tilde{\xi }}_{d}=\tau \xi_{d},\;{\tilde{\rho }}_{d}=\rho_{d}\tau n_{0},\;{\tilde{S}}_{d}=\frac{S_{d}\tau n_{0}}{d_{0}},\\ \; & {\tilde{D}}_{o}=\frac{D_{o}}{D},\;{\tilde{\xi }}_{o}=\tau \xi_{o},\;{\tilde{\rho }}_{o}=\frac{\rho_{o}\tau n_{0}}{o_{\max}},\;{\tilde{S}}_{o}=S_{o}\tau n_{0}. \end{array}$$

For notational simplicity, unless otherwise stated, we drop the tildes and write all variables in their dimensionless form from here on. After applying the nondimensionalization defined above, the governed PDEs take the form

(6) $$\begin{array}{cc} \partial_{t}n & =D_{n}\Delta n-\nabla \cdot \left(\frac{\chi_{0}}{1+\alpha c}n\nabla c\right), \end{array}$$

(7) $$\begin{array}{cc} \partial_{t}c & =D_{c}\Delta c-\xi_{c}c+\eta \sum_{a\in \Lambda_{t}^{h}}\phi_{a}-\lambda c\sum_{v\in V_{t}}\phi_{v}, \end{array}$$

(8) $$\begin{array}{cc} \partial_{t}d & =D_{d}\Delta d-\xi_{d}d-\rho_{d}d\sum_{a\in \Lambda_{t}}\phi_{a}+S_{d}\sum_{v\in V_{t}}\phi_{v}, \end{array}$$

(9) $$\begin{array}{cc} \partial_{t}o & =D_{o}\Delta o-\xi_{o}o-\rho_{o}\sum_{a\in \Lambda_{t}}\phi_{a}+S_{o}\left(1-o\right)\sum_{v\in V_{t}}\phi_{v}, \end{array}$$

All fields ϕ∈{n,c,d,o} satisfy homogeneous Neumann (no-flux) boundary conditions in dimensionless form:

$$\begin{array}{c} \vec{n}\cdot \nabla \phi {|}_{\partial U}=0. \end{array}$$

The dimensionless coefficients $D_{n},D_{c},\ldots$ represent ratios relative to the chosen characteristic scales *D* and τ. For example, $D_{c}$ is the diffusion coefficient of TAF normalized by *D*, and $D_{n}$ is the normalized endothelial cell motility coefficient. The nondimensionalization $\alpha =c_{0}/k_{1}$ provides one such example of this scaling. Since all variables are in their dimensionless form, unless otherwise stated, t,x,y,n,c,… are understood to be dimensionless quantities. Accordingly, the numerical time steps Δt for PDE and $\Delta t^{\prime }$ for ABM in Section 3.2 are also nondimensional.

Most of our work is performed under the nondimensionalized system (Equations (6)–(9)). All PDEs displayed in Section 2 and Section 4 are in dimensionless form unless explicitly labeled as dimensional; Table 3 lists dimensional parameter values and units.

Table 4 gives an overview of all model parameters used in the study, both for the PDE system, the ABM, and nondimensionalization. The table lists each parameter, along with a brief description of how our modeling framework incorporates it.

We state model assumptions compactly. We assume fixed per cell oxygen and drug consumption rates, linear isotropic diffusion and decay for drug, chemotactic guidance of angiogenic tip cells by TAF gradients, neutral stochastic mutation processes, and a simplified DNA damage repair model without detailed biochemical kinetics. These assumptions restrict scope but keep the model analytically and computationally tractable.

This PDE model gives a rigorous mathematical framework for tumor growth and resistance to therapy, grounded in biological mechanisms. Reaction–diffusion equations capture microenvironmental heterogeneity through diffusion, decay, and local production or consumption. They account for effects such as hypoxic cores, where blood vessels poorly supply certain regions, limited drug penetration, and TAF-induced chemotactic angiogenesis. This continuum PDE formulation sets the stage for the agent-based rules described next. The bidirectional PDE–ABM coupling enables multiscale modeling of cellular heterogeneity. Cells respond to local signaling fields while dynamically altering them through production and consumption. This framework is computationally efficient and readily extends to incorporate advanced cellular behaviors and microenvironmental interactions.

### 2.2. Agent-Based Rules

#### 2.2.1. ABM Tumor Cell Rules

We implement tumor cells as discrete agents embedded in a two-dimensional lattice framework. These agents interact with continuum microenvironmental fields governed by PDEs in Section 2.1. Each cell follows biologically motivated and mathematically explicit rules for proliferation, apoptosis, mutation, and motility. The choice of the lattice neighborhood structure constrains spatial interactions.

**Figure 3:**
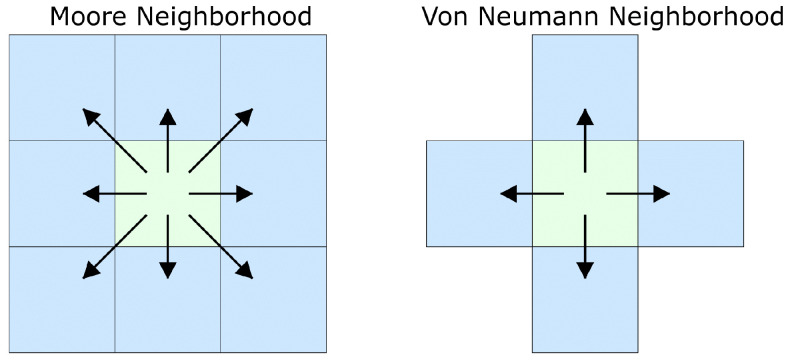
Comparison of Von Neumann and Moore neighborhood structures on a 2D lattice. The Von Neumann neighborhood includes the four orthogonally adjacent lattice sites (left, right, down, up). The Moore neighborhood additionally includes diagonal neighbors. This schematic illustrates how tumor and tip cells detect neighboring agents and respond to local environmental cues.

Figure 3 illustrates two common configurations. The Von Neumann neighborhood comprises the four adjacent lattice sites (left, right, down, up), while the Moore neighborhood additionally incorporates the four diagonal sites. Tumor cells use the Von Neumann neighborhood when computing movement probabilities, whereas branching checks Moore neighbors for available space.

Tumor cells are not confined to lattice positions, as continuous Brownian dynamics describe their motion (Equation (10)). In contrast, lattice positions constraint tip and vessel agents. Therefore, only the four cardinal directions in the Von Neumann neighborhood are allowed for tip motion.

Table 5 summarizes the neighborhood structures used for different cellular processes in the ABM component.

(i) Cell trait

We represent each tumor cell by a state vector tracking lineage identifier ${\mathrm{id}}_{a}$ (dimensionless), its position $a^{x}\left(t\right)$ (unit: m), local oxygen concentration $a^{o}\left(t\right)$ (unit: mol/$m^{3}$) accumulated drug exposure $a^{d}\left(t\right)$ (unit: $\left(\mathrm{mol}\cdot s\right)/m^{3}$) and DNA damage $a^{dam}\left(t\right)$ (unit: $\left(\mathrm{mol}\cdot s\right)/m^{3}$), death threshold $a^{death}\left(t\right)$ (unit: $\left(\mathrm{mol}\cdot s\right)/m^{3}$), the time elapsed since the last division $a^{age}\left(t\right)$ (unit: s), and the cell cycle duration $a^{mat}\left(t\right)$ (unit: s):

$$\begin{array}{c} a=\left\{{\mathrm{id}}_{a},\;a^{x}\left(t\right),\;a^{o}\left(t\right),\;a^{d}\left(t\right),\;a^{dam}\left(t\right),\;a^{death}\left(t\right),\;a^{age}\left(t\right),\;a^{mat}\left(t\right)\right\}. \end{array}$$

For clarity, we provide the complete definition of the state vector in Appendix C.

(ii) Cell motility

At the individual-cell level, tumor motion follows independent and identically distributed (i.i.d.) Brownian dynamics:

(10) $$\begin{array}{c} da^{x}=\varepsilon dW_{t}^{a},\;{\left\{W_{t}^{a}\right\}}_{a\in \Lambda_{t}}\;i.i.d.\;\mathrm{Brownian}\;\mathrm{motions}. \end{array}$$

Here, the parameter ε=0.0215 (dimensionless) directly controls the strength of random fluctuations in cell trajectories. Positions update via Euler–Maruyama:

$$\begin{array}{c} a^{x}\left(t+\Delta t\right)=a^{x}\left(t\right)+\varepsilon \sqrt{\Delta t}\;Z_{t}^{a},\;Z_{t}^{a}∼N\left(0,1\right)\;i.i.d. \end{array}$$

Random movement, combined with displacement from daughter cell division, constitutes the only migration mechanisms for tumor cells. Cells move in continuous space and freely occupy positions off the lattice. We enforce reflective boundaries to prevent tumor agents from leaving the domain.

(iii) Cell sensing

At each ABM time step, tumor cells sense local chemical fields:

$$\begin{array}{c} a^{o}\left(t+\Delta t\right)=o\left(a^{x}\left(t\right),t\right),\;a^{d}\left(t+\Delta t\right)=a^{d}\left(t\right)+d\left(a^{x}\left(t\right),t\right)\Delta t. \end{array}$$

The drug level $a^{d}$ represents cumulative exposure rather than instantaneous intracellular drug concentration. It also implies that the drug enters and remains in the cell linearly. We omit explicit intracellular efflux and degradation, which contribute to drug resistance in many cancers [43]. This death threshold abstraction implicitly encompasses these resistance-related processes, and we left the incorporation of these mechanisms for future work.

(iv) Damage accumulation and death criteria

DNA damage evolves through the following:

$$\begin{array}{c} a^{dam}\left(t+\Delta t\right)=a^{dam}\left(t\right)+\left[d\left(a^{x}\left(t\right),t\right)-p_{r}a^{dam}\left(t\right)\right]\Delta t. \end{array}$$

Here, $p_{r}$ denotes the DNA repair rate. Cell death occurs when $a^{dam}\left(t\right)>a^{death}\left(t\right)$, and death occurs immediately at the current time step. The death threshold follows:

$$\begin{array}{c} a_{R}^{death}=Th_{\mathrm{multi}}\cdot a_{S}^{death}. \end{array}$$

Here, $Th_{\mathrm{multi}}$ is the multiplicative resistance factor that scales the death threshold in resistant clones. We set baseline death threshold value $a_{S}^{death}=0.5$ in nondimensional units. In the preexisting resistance scenario, we fix $Th_{\mathrm{multi}}=5$. In the mutation-acquired resistance scenario, $Th_{\mathrm{multi}}$ evolves according to the neutral mutation rule and is updated by a random multiplier r∼Uniform[0.7,1.7]. Since we impose phenotypic constraint in Equation (12), $Th_{\mathrm{multi}}\in \left[0.5,4\right]$.

(v) Division rules

Cells are classified by the oxygen level $a^{o}\left(t\right)$. Cells are normoxic ($a\in \Lambda_{t}^{n}$) if the local nondimensional oxygen concentration satisfies $a^{o}\left(t\right)>o_{\mathrm{hyp}}$. Cells are hypoxic ($a\in \Lambda_{t}^{h}$) if $o_{\mathrm{apop}}<a^{o}\left(t\right)\leq o_{\mathrm{hyp}}$, and apoptotic (removed immediately from $\Lambda_{t}$) if $a^{o}\left(t\right)\leq o_{\mathrm{apop}}$ during the current time step. Here, $o_{\mathrm{apop}}<o_{\mathrm{hyp}}$ are the critical thresholds. In our nondimensionalization, we set $o_{\max}=1$ as the reference oxygen concentration. The hypoxia and apoptosis thresholds are set to $o_{\mathrm{hyp}}=0.25$ and $o_{\mathrm{apop}}=0.05$, respectively (see Table 3).

Cells age only when normoxic:

$$\begin{array}{c} a^{age}\left(t+\Delta t\right)=\left\{\begin{array}{cc} a^{age}\left(t\right)+\Delta t & \mathrm{if}\;a^{o}\left(t\right)>o_{\mathrm{hyp}},\\ a^{age}\left(t\right) & \mathrm{otherwise}. \end{array}\right. \end{array}$$

Normoxic cells divide when $a^{age}\left(t\right)\geq a^{mat}\left(t\right)$, where maturation time depends on division rate $\alpha_{n}$:

$$\begin{array}{c} a^{mat}\left(t\right)=\frac{\log(2)}{\alpha_{n}}. \end{array}$$

Local density is

$$\begin{array}{c} F\left(x,t\right)=\sum_{\tilde{a}\in \Lambda_{t}}\chi_{B_{R_{c}}}\left(x-{\tilde{a}}^{x}\left(t\right)\right), \end{array}$$

which counts the number of cells within radius $R_{c}$ of a given cell position $a^{x}$, and it regulates crowding during proliferation. If F(x,t) at a cell’s location exceeds the threshold $F_{\max}$, the cell is considered overcrowded and cannot divide at that time step. Instead, it retains its current age and retries in the next time step.

In two dimensions, when we model cells as disks that interact via repulsive forces such as the Lennard–Jones potential, they spontaneously form close-packed hexagonal arrangements [19]. In this ideal packing, each cell has roughly six immediate neighbors, suggesting a natural cutoff of $F_{\max}=6$. Tumor tissue, however, is typically more densely packed than the hexagonal arrangement. To reflect this biological observation while still preventing unrealistic overlap, we define

$$\begin{array}{c} F_{\max}=10. \end{array}$$

This value sets the maximum allowable local occupancy. In other words, $F_{\max}$ defines the crowding threshold, specifying the largest number of neighboring cells within radius $R_{c}$ that still permits cell division.

When $F\left(a^{x}\left(t\right),t\right)\leq F_{\max}$, the mother divides into two daughters, $a_{1}$ and $a_{2}$. Their positions are

(11) $$\begin{array}{cc} a_{1}^{x}\left(t\right) & =a^{x}\left(t\right),\\ a_{2}^{x}\left(t\right) & =a^{x}\left(t\right)+0.1\left(\cos\left(2\pi \theta \right)\Delta x,\sin\left(2\pi \theta \right)\Delta y\right), \end{array}$$

where θ∼Uniform[0,1] and Δx,Δy are spatial discretizations. The small displacement 0.1Δx prevents perfect overlap while remaining negligible relative to the cell diameter, effectively positioning the two daughter cells nearly on top of each other. A slightly larger displacement or random perturbation could better mimic mechanical pushing, but overlap is negligible because the $F_{\max}$ check is in place. If $a_{2}^{x}\left(t\right)$ overlaps with another existing cell center ${\tilde{a}}^{x}\left(t\right)$, θ is resampled until a valid non-overlapping position is found.

(vi) Inheritance

Upon division, daughter cells $a_{1}$ and $a_{2}$ inherit half of the mother cell *a*’s damage and drug load:

$$\begin{array}{c} a_{i}^{dam}\left(t\right)=\frac{1}{2}a^{dam}\left(t\right),\;a_{j}^{d}\left(t\right)=\frac{1}{2}a^{d}\left(t\right),\;i,j\in \left\{1,2\right\}, \end{array}$$

and reset their age to 0. The local oxygen concentration at its position determines the oxygen level of each daughter cell. Daughter cells inherit their mother’s death threshold, proliferation rate, and oxygen consumption rate, with the possibility of mutation in these traits. This mutational mechanism enables initially identical cells to evolve heterogeneous phenotypes over time.

We initialize the zeroth-generation cells with the state vector:

$$\begin{array}{c} a=\left\{\left(k\right),a^{x}\left(t\right),o\left(a^{x}\left(t\right),t\right),0,0,T_{k},N_{k},M_{k}\right\}. \end{array}$$

We initialize the cell cycle duration as

$$\begin{array}{c} M_{k}∼\mathrm{Uniform}\left[3.24\times 10^{4},\;3.96\times 10^{4}\right]\;s. \end{array}$$

Converting to nondimensional units with $\tau =5.76\times 10^{4}$ s yields ${℘}_{age}=M_{k}\in \left[0.56,0.69\right]$. For consistency with Table 3, we use ${℘}_{age}$ to denote the distribution of cell-cycle durations. To represent biologically realistic heterogeneity in cell cycle progression, we sample initial cell ages $N_{k}$ from the uniform distribution $N_{k}∼\mathrm{Uniform}\left[0,M_{k}\right]$. At last, $T_{k}$ is the death threshold. We consider two resistance scenarios.

(i) Preexisting resistance. At initialization, 1% of cells are specified as resistant, with $T_{k}=a_{R}^{\mathrm{death}}=2.5$, while the remaining 99% are sensitive, with $T_{k}=a_{S}^{\mathrm{death}}=0.5$.
(ii) Spontaneous mutation. All cells are initialized as sensitive, with $T_{k}=a_{S}^{\mathrm{death}}=0.5$.

(vii) Mutation

Existing mutation models include [38]: (i) random mutation, in which one of the predefined N>1 phenotypes is selected with equal probability $p=\frac{1}{N}$ during mutation, and (ii) linear mutation, where phenotypes evolve deterministically along a predefined path of increasing resistance and aggressiveness.

Although linear mutation avoids abrupt phenotypic jumps, it enforces a deterministic progression toward aggressive phenotypes. It also disregards microenvironmental selection pressures. To address these limitations, we introduce a neutral non-directional mutation algorithm that prevents abrupt trait shifts and enables unbiased phenotypic evolution. Mutations follow a Poisson process with intensity µ>0 per cell per time step:

$$\begin{array}{c} P\left(\mathrm{mutation}\;\mathrm{in}\;\left[t,t+\Delta t\right]\right)=1-e^{-µ\Delta t}\approx µ\Delta t\;\;\mathrm{for}\;\;\;µ\Delta t\ll 1 \end{array}$$

On a mutation event, we draw independent multipliers $r_{i}∼\mathrm{Uniform}\left[0.7,1.7\right]$ for each mutable trait $x_{i}$ (e.g., $\alpha_{n}$, $a^{death}$, oxygen consumption), not a single multiplier applied to all traits. The new trait value is

$$\begin{array}{c} x_{i,\mathrm{new}}=r_{i}\times x_{i,\mathrm{current}}. \end{array}$$

We enforce biologically plausible bounds immediately to ensure traits remain within the specified phenotype envelope:

(12) $$\begin{array}{c} 0.5\;x_{i,\mathrm{baseline}}\leq x_{i,\mathrm{new}}\leq 4\;x_{i,\mathrm{baseline}}. \end{array}$$

This design: (i) prevents a single mutation from producing unrealistically extreme trait values, (ii) allows independent evolution of different traits, and (iii) preserves the neutral (non-directional) character of the scheme.

The biological rationale and caveats for the chosen multiplier range $r_{i}\in \left[0.7,1.7\right]$ are that it represents moderate per-event effect sizes. A single mutation can increase a trait by up to 70% or reduce it by up to 30%. For example, under a single mutation, the probability that a trait increases by at least 50% is

$$\begin{array}{c} P\left(r_{i}\geq 1.5\right)=\frac{1.7-1.5}{1.7-0.7}=0.2, \end{array}$$

and the probability it increases by at least 40% is 0.30.

Because the scheme is multiplicative, larger phenotype changes (e.g., a 3× increase in the death threshold) require several successive mutations. Using the mean multiplier $E(r_{i})=1.2$, the expected number of mutation events *n* such that $1.2^{n}\geq 3$ is approximately n≈6. These simple calculations demonstrate the following: (i) single mutations are moderate in size, and (ii) high-level resistance accumulates over multiple events, consistent with gradual phenotype evolution rather than abrupt jumps.

Classical mutation models explicitly tie resistance acquisition to cell division events [38,40]. In contrast, we implement mutation as a time-continuous Poisson process with intensity µ, independent of cell division. At each event, every mutable trait (including $Th_{\mathrm{multi}}$) is independently rescaled by $r_{i}∼\mathrm{Uniform}\left[0.7,\;1.7\right]$, with immediate enforcement of the biologically plausible bounds $0.5\leq Th_{\mathrm{multi}}\leq 4$.

The independence assumption reflects biological evidence that mutations can arise from internal cell mechanisms and environmental stresses, rather than being strictly division-dependent [44]. The choice of a Poisson process models the memoryless property of mutation events, decoupling mutation timing in a way that is both analytically tractable and reproducible, regardless of a cell’s division history.

For a comprehensive introduction to stochastic mutation processes, we refer the reader to Ewens [45]. Future iterations of the model could refine this approach by incorporating environment-dependent mutation rates or kinetic signaling modules.

**Remark** **1.** (Limitation: Neutral Mutation Assumption). *In our current framework, resistance mutations are implemented as neutral, meaning they impose a direct fitness cost. This abstraction isolates the effects of microenvironmental heterogeneity without confounding selective pressures. Biologically, however, the acquisition of resistance can impose metabolic burdens or reduce proliferative capacity. Evidence from microbial systems demonstrates that antibiotic resistance acquisition is often associated with a fitness cost for resistant organisms [46]. In cancers, the presence of drug-tolerant persisters (DTPs) (i.e., cells with intrinsically slow growth kinetics that can survive initial drug exposure) further supports the notion that resistance may carry a fitness penalty [47]. Such costs can reshape clonal competition and potentially delay or suppress the expansion of resistant cells. Future extensions should therefore relax the neutrality assumption and incorporate non-neutral mutations. One possible strategy is to link the resistance threshold $a^{death}$ to a reduced proliferation rate $\alpha_{n}$. This modification would enable systematic exploration of trade-offs between therapeutic survival and baseline fitness, thereby aligning the model more closely with experimental observations of resistance evolution.*

**Figure 4:**
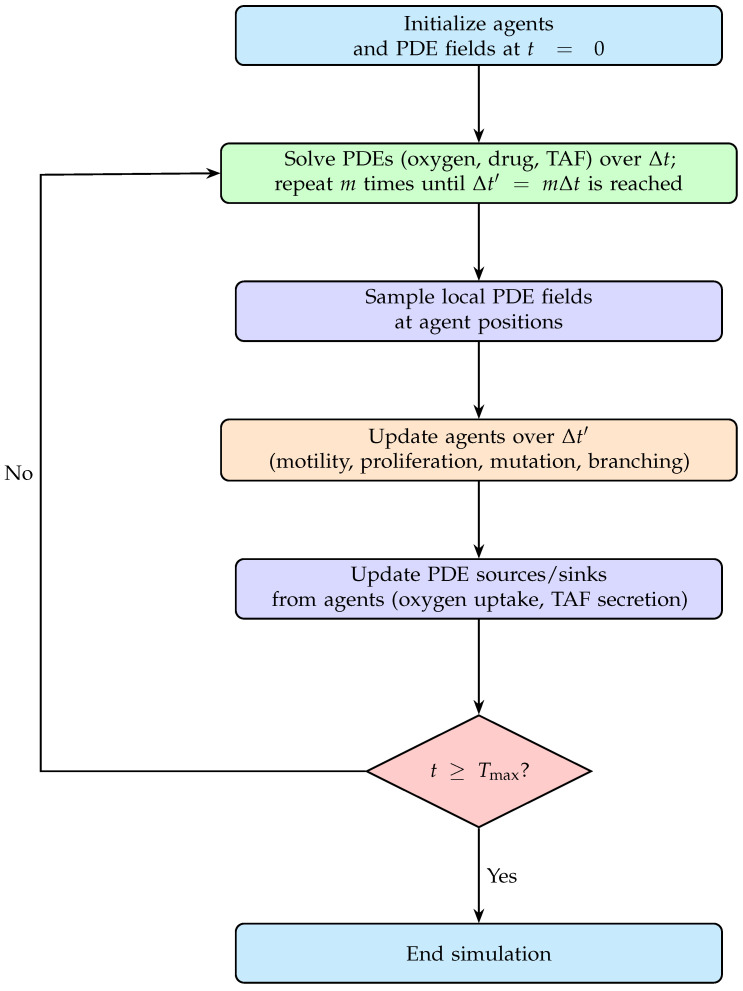
Flowchart of ABM–PDE coupling. PDEs advance with step Δt, ABM with step $\Delta t^{\prime }$. At each global iteration, agents sample local PDE fields and, in turn, update PDE sources, ensuring two-way coupling.

We implement the tumor cell rules described above within the hybrid solver. To ensure clarity and reproducibility, we summarize their integration with the PDE fields in the global time-stepping flowchart (Figure 4). The computational update steps appear explicitly in the PDE-ABM solver (Algorithm A1) and the ABM update operator (Algorithm A2) in Appendix D.

#### 2.2.2. ABM Angiogenesis Rules

We model endothelial tip cells and vessel cells as discrete agents, denoted $b\in T_{t}$ and $v\in V_{t}$, respectively. Chemotaxis, branching, and anastomosis drive angiogenesis. The set $T_{t}$ contains all tip cells at time *t*.

(i) Agent trait

Each endothelial tip cell $b\in T_{t}$ tracks its spatial position $b^{x}\left(t\right)$ (unit: m), age $b^{age}$ (unit: s) since last branching, and a lineage identifier ${\mathrm{id}}_{b}$ (dimensionlessm) that records ancestry:

$$\begin{array}{c} b=\{{\mathrm{id}}_{b},b^{x}\left(t\right),\;b^{age}\left(t\right)\}. \end{array}$$

This lineage scheme parallels the tumor-cell identifiers and ensures consistent reconstruction of vascular branching trees. Full definitions and update rules are provided in Appendix C.

The tip cell age evolves as follows:

$$\begin{array}{c} b^{age}\left(t+\Delta t\right)=b^{age}\left(t\right)+\Delta t. \end{array}$$

Tip migration uses a lattice of Von Neumann neighbors according to chemotaxis-diffusion dynamics. Directional movement probabilities $P_{0}$ (stationary), $P_{1}$ (left), $P_{2}$ (right), $P_{3}$ (down), and $P_{4}$ (up) are derived from the TAF gradient (Equation (17)). The movement probabilities link discrete tip motion to the continuum TAF field; Section 3.2 gives the explicit derivations and numerical implementation.

(ii) Anastomosis

Anastomosis occurs when a migrating tip enters a lattice site occupied by another tip or vessel agent in the Von Neumann neighborhood. Upon anastomosis, the invading tip ceases migration and branching, converting into a vessel segment and contributing to a closed-loop vascular network. This rule not only yields dynamically evolving vascular structures with loops, consistent with physiological neovascularization, but also prevents tip collisions, ensuring biologically realistic vascular connectivity. Classical angiogenesis models have incorporated similar angiogenesis formulations [25].

**Figure 5:**
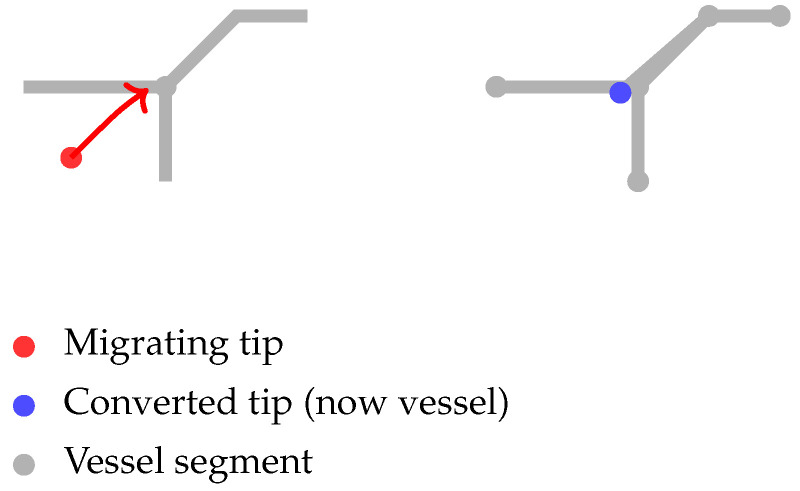
Anastomosis: A migrating tip enters an occupied site, stops migrating/branching, and converts into a vessel segment.

For a schematic illustration of the branching of the tip and the formation of vascular loops through anastomosis, see Figure 9 in [25]. Figure 5 shows the anastomosis process. Here, a migrating tip enters an already occupied site. At this point, it stops further migration and branching, converting into a vessel segment. Therefore, this event prevents tip collisions and stabilizes the emerging vascular architecture. It can even form a closed loop in the network.

(iii) Branching

We model endothelial tip branching as a Poisson process, with the following intensity:

$$\begin{array}{c} \lambda_{br}\left(b,t\right)=c_{br}\frac{c(b^{x}\left(t\right),t)}{{∥c\left(\cdot ,t\right)∥}_{\infty }}\;H\left(b^{age}\left(t\right)-\psi \right). \end{array}$$

Here, $c_{br}$ denotes the baseline branching rate (nondimensional value, corresponding to a dimensional unit of 1/s). The term $c(b^{x}\left(t\right),t)$ represents the local TAF concentration at the tip position $b^{x}$. Finally, ${∥c\left(\cdot ,t\right)∥}_{\infty }$ denotes the domain-wide maximum TAF concentration at time *t*. The Heaviside function *H* enforces a minimum branching age: no branching occurs until $b^{age}\left(t\right)\geq \psi$, with ψ=1.125 (nondimensional time units).

Normalizing $c(b^{x},t)$ by ${∥c\left(\cdot ,t\right)∥}_{\infty }$ bounds the branching intensity by $c_{br}$. This normalization provides a convenient scaling of the stochastic intensity. However, it introduces a nonlocal dependence: each tip implicitly senses the maximum TAF concentration across the tissue domain. Biologically, this global sensing assumption may not be mechanistically realistic, since endothelial tip cells are known to respond to local gradients of VEGF/TAF rather than global maxima. Nevertheless, this choice stabilizes the numerical range of $\lambda_{br}$.

The nonlocality of ${∥c\left(\cdot ,t\right)∥}_{\infty }$ represents a modeling simplification. In more physiologically grounded descriptions, we can express the branching intensity as a saturating function of the local TAF concentration. For example, the Michaelis–Menten type is

$$\begin{array}{c} \lambda_{br}\left(b,t\right)=c_{br}\;\frac{c(b^{x}\left(t\right),t)}{K_{c}+c\left(b^{x}\left(t\right),t\right)}, \end{array}$$

where $K_{c}$ is the half-saturation concentration. Incorporating such nonlinear local response functions is a promising extension for future refinements of the model.

A branching event occurs if $b^{age}>\psi$, at least one Moore neighbor is vacant, and

$$\begin{array}{c} \mathrm{Uniform}\left[0,1\right]<1-e^{-\lambda_{br}\left(b,t\right)\Delta t}\approx \lambda_{br}\left(b,t\right)\Delta t. \end{array}$$

**Figure 6:**
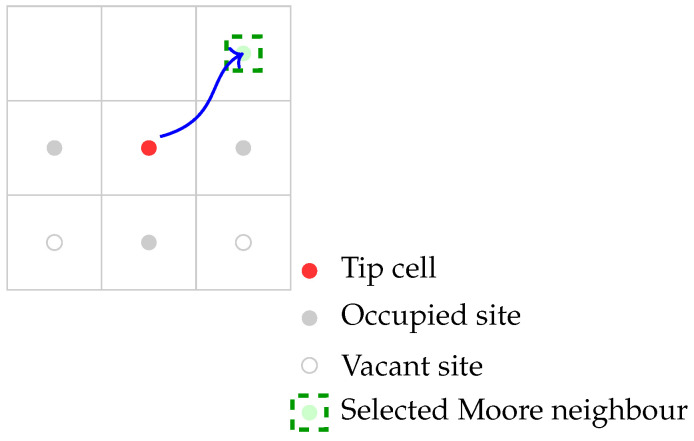
Tip branching: one daughter remains at the original site, while the other occupies a vacant Moore neighbor (diagonals allowed).

Upon branching, one daughter cell remains at the original location, while the other occupies a randomly selected vacant site in the Moore neighborhood, resetting their age to 0. Tip motions are restricted to the four orthogonal directions (Von Neumann neighborhood), whereas branching may place a daughter on a diagonal (Moore) site if vacant. Figure 6 illustrates the branching mechanism. When the stochastic branching criterion holds, the parent tip produces two daughter tips. One remains at the original site, while the other occupies a randomly chosen vacant Moore neighbor, which may include diagonal sites. This rule allows the expansion of vascular network, increasing the likelihood of vessel interconnections.

(iv) Proliferation

Endothelial tip cell proliferation follows a fixed doubling time $\tau_{\mathrm{tip}}=1.125$ ($6.48\times 10^{4}$ s), consistent with classical hybrid angiogenesis models [25]. Each division elongates the sprout by one cell length. During division, one daughter tip continues migration along the sprout direction, while the other becomes a new stalk segment at the original tip location.

Both tip and stalk (non-tip) endothelial cell proliferation contribute to sprout elongation, but experimental and computational evidence indicate that stalk-cell proliferation is mechanically induced by tension generated at the advancing tip. As noted by Santos-Oliveira et al. [48], “the tip cell has the role of creating a tension in the cells that follow its lead. On those first stalk cells, this tension produces strain and/or empty spaces, inevitably triggering cell proliferation. The new cells occupy the space behind the tip, the tension decreases, and the process restarts.” Thus, tip-driven stalk proliferation results in elongation by approximately one cell length per cycle, functionally equivalent to the elongation produced by tip proliferation. To simplify the model while preserving this biological effect, we therefore represent proliferation solely through tip cell proliferation, which serves as an effective proxy for the combined contributions of both tip and stalk endothelial cells. This formulation is consistent with classical angiogenesis models, e.g., [25] and Figure 16 therein.

We implement the tip proliferation rule as follows:

$$\begin{array}{c} \mathrm{Tip}\;\mathrm{cell}\;\mathrm{at}\;\mathrm{position}\;x_{\mathrm{tip}}\;\underset{\mathrm{division}\;\mathrm{at}\;t+\tau_{\mathrm{tip}}}{\to }\;\left\{\begin{array}{c} \mathrm{Daughter}\;\mathrm{tip}\;\mathrm{at}\;x_{\mathrm{tip}}+\Delta x,\\ \mathrm{Stalk}\;\mathrm{segment}\;\mathrm{at}\;x_{\mathrm{tip}}. \end{array}\right. \end{array}$$

**Figure 7:**
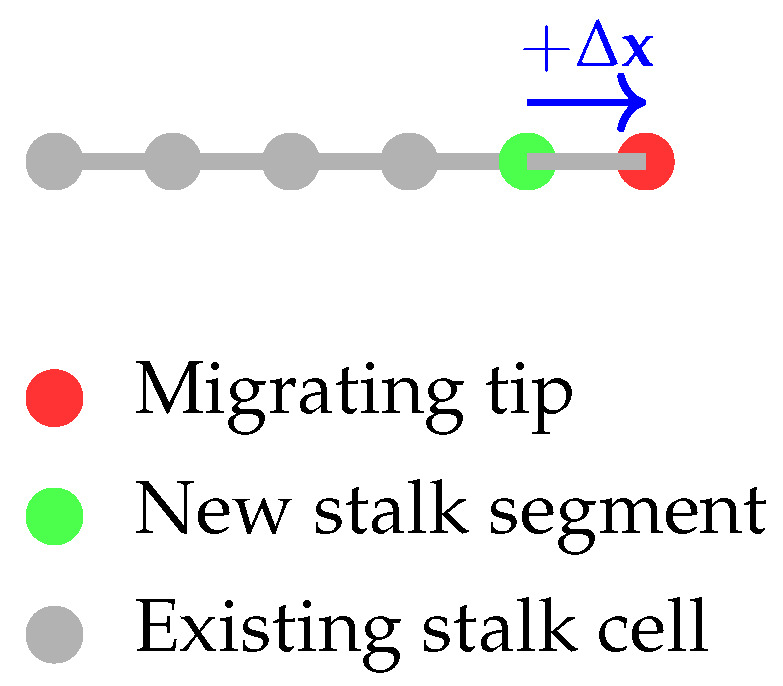
Tip proliferation schematic. After a fixed doubling time $\tau_{\mathrm{tip}}=18$ h, the tip cell (red) divides. One daughter remains a tip that migrates forward by one cell length (+Δx), while the other becomes a stalk segment (green) at the former tip position. This rule elongates the sprout without generating redundant tip cells.

Here, Δx represents the tip migration along the sprout direction. Each tip division adds one new stalk segment behind the migrating tip without generating an additional tip cell at the same location, avoiding redundancy with the branching rule. Figure 7 illustrates the tip proliferation schematically. The red and green nodes represent the migrating tip and the newly created stationary stalk segment, respectively, and the arrow indicates the tip movement along the sprout. At each fixed doubling time, the active tip divides asymmetrically: one daughter advances the sprout by migrating forward, while the other differentiates into a stalk cell at the former tip location. This mechanism ensures that sprout elongation is driven solely by tip proliferation while maintaining exactly one active tip per sprout.

Our current model does not include vessel pruning or regression. Once a site becomes a vessel, it remains one permanently, and regression cannot occur. Such modeling extensions incorporating vessel pruning and regression remain for future work.

(v) Angiogenic network

The motion of an individual endothelial cell at the capillary sprout tip governs the entire sprout’s movement because the remaining endothelial cells lining the sprout wall are contiguous [25]. Thus, the cumulative paths of tip cells define the angiogenic network:

$$\begin{array}{c} A_{t}=\underset{b\in T_{t}}{⋃}\left\{b^{x}\left(s\right):0\leq s\leq t\right\}. \end{array}$$

Each lattice site intersecting $A_{t}$ becomes a vessel agent $v=\left\{v^{x}\left(t\right)\right\}\in V_{t}$, represented geometrically as a circle of radius $R_{c}$ inscribed within the site grid. We encode this geometry by the normalized indicator function $\phi_{v}$ for $v\in V_{t}$. Vessel agents serve as sources of oxygen and drug delivery in the PDEs, thereby coupling the continuum microenvironmental fields with the ABM.

The present formulation omits explicit mechanical interactions (e.g., Lennard–Jones-type potentials) between endothelial and tumor cells. As a result, cells may overlap (up to $F_{\max}$). This simplification follows many prior hybrid PDE-ABM models, which also neglect cell–cell forces for tractability [25,38,40]. For instance, Spill et al. [49] incorporated only random motion and chemotaxis as tip cell motility mechanisms, excluding mechanical interactions.

While this choice isolates the role of chemotaxis and proliferation in angiogenic dynamics, it does not prevent cell overlap. Incorporating simple repulsive-force interactions could enhance biological realism in future work. Established force-based models offer a natural way to integrate such effects [18,19,50]. Their exclusion here allows us to focus specifically on tumor evolution under microenvironmental regulation.

For clarity and reproducibility, the pseudocode in Algorithm A2 summarizes the ABM module per time step. The global flowchart (Figure 4) illustrates its coupling with the PDE fields, including sampling of local concentrations and updating agent-driven sources, and Algorithm A1 details the computational steps of the hybrid PDE-ABM framework.

We present broader limitations and suggested future work in the Discussion. As a prelude to the numerical implementation, we now provide a consolidated summary of the parameter values employed. This bridge is important: the selection and calibration of dimensional quantities directly constrain the behavior of both PDE fields and agent-based dynamics, and hence determine the realism and reproducibility of the simulations.

## 3. Numerical Implementation

### 3.1. Parameterization and Nondimensionalization

To establish biologically relevant scales while ensuring numerical stability, we nondimensionalize the model using characteristic parameters:

$$\begin{array}{c} \tilde{n}=\frac{n}{n_{0}},\;\tilde{c}=\frac{c}{c_{0}},\;\tilde{t}=\frac{t}{\tau }. \end{array}$$

We choose the length scale $L=5\times 10^{-3}$ m and set time scale $\tau =L^{2}/D=5.76\times 10^{4}$ s, where *D* denotes a representative diffusion rate. We set the reference cell density to $n_{0}=6.4\times 10^{13}\;\mathrm{cell}/m^{3}$, representative of a densely packed tissue rather than a sparse culture. Reported tumor cell diameters vary between 10 and 100µm depending on tumor type [38]. Here, we adopt 25µm as a representative diameter such that $2R_{c}=\Delta x$, i.e., each lattice grid cell accommodates exactly one tumor cell. The reference concentration is set to $c_{0}=1.0\times 10^{-7}\;\mathrm{mol}/m^{3}$, following Anderson et al. [25]. This value corresponds to a typical TAF concentration scale used in prior experimental studies to test the endothelial chemotactic coefficient $\chi_{0}$. See Appendix E for the detailed parameter estimation.

It is important to note that $n_{0}$ also serves as the characteristic scale for the agent-based source and sink terms $\sum_{a\in \Lambda_{t}}\phi_{a}$ and $\sum_{v\in V_{t}}\phi_{v}$. Since each normalized indicator function $\phi_{a}$ and $\phi_{v}$ has units of $\mathrm{cell}/m^{3}$, these sums naturally represent local cell densities (In the three-dimensional case, the normalized indicator functions $\phi_{a},\phi_{v}$ have units of $\mathrm{cell}/m^{3}$, interpreted as per-volume cell counts (i.e., local cell density). Because most experimental and modeling parameter values reported in the literature are in volumetric (3D) units, we adopt a three-dimensional reference frame for nondimensionalization.). Therefore, $n_{0}$ provides the appropriate volumetric reference scale for converting per cell production or uptake coefficients ($\eta ,\lambda ,\rho_{d},S_{d},\rho_{o},S_{o}$) into terms with correct physical dimensions. This interpretation ensures consistency between the literature-reported per cell rates (which we list directly in Table 3) and our nondimensionalization procedure. We refer readers for the details of the relevant unit conversions to Appendix E.

The nondimensionalization in Equation (5) defines dimensionless parameters as ratios relative to the chosen characteristic scales. For example, ${\tilde{D}}_{n}$ and ${\tilde{D}}_{c}$ denote diffusion coefficients normalized by the reference diffusivity *D*, ${\tilde{\xi }}_{c}$ and ${\tilde{\xi }}_{o}$ represent decay rates scaled by the characteristic time τ, and $\tilde{\eta }$, $\tilde{\lambda }$, ${\tilde{\rho }}_{d}$, and ${\tilde{\rho }}_{o}$ correspond to per cell production or uptake rates rescaled by the reference cell density $n_{0}$. The chemotactic sensitivity is characterized by ${\tilde{\chi }}_{0}$, and $\alpha =c_{0}/k_{1}$ quantifies the ratio between the reference TAF concentration and the receptor half-saturation constant. Collectively, these dimensionless quantities measure the relative strength of competing processes on biologically relevant spatiotemporal scales. The resulting dimensionless quantities are summarized in Table 3, which provides the authoritative parameter set used in all simulations. These values are also available in machine-readable format (params.json, params.csv) in the GitHub Repository in the Data Availability Statement Section. Under this scaling, a dimensionless time unit of t=0.5 corresponds to $2.88\times 10^{4}$ s, consistent with experimental tumor growth measurements [41,42].

With the nondimensional model and parameter set fixed, we now describe the numerical methods used to integrate the PDEs and update the agent rules.

### 3.2. Numerical Implementation

Building upon the mathematical framework in Section 2.1 and Section 2.2, we now describe the computational methodology used to simulate the coupled tumor–vascular system. The focus is on discretization schemes that ensure numerical stability and accurate coupling between continuum fields and agent-based dynamics.

We employ a hybrid numerical strategy. The reaction–diffusion equations for drug, oxygen, and TAF (Equations (2)–(4)) are solved using the ADI method, which is unconditionally stable for linear diffusion and permits larger time steps than explicit schemes. The endothelial density equation (Equation (1)), which contains a nonlinear chemotactic flux, is advanced by a forward Euler method subject to the Courant–Friedrichs–Lewy (CFL) condition [51]:

(13) $$\begin{array}{c} \Delta t\leq \min\left\{\frac{\Delta x^{2}}{4D_{n}},\;\frac{\Delta x}{{2∥\chi \left(c\right)\nabla c∥}_{\infty }}\right\}, \end{array}$$

where ${∥\cdot ∥}_{\infty }$ denotes the $L^{\infty }\left(U\right)$-norm. This restriction guarantees stability and prevents spurious oscillations.

The computational domain is discretized on a uniform Cartesian grid with $\left(N_{x}+1\right)\times \left(N_{y}+1\right)=100\times 100$ nodes, spanning a $2.5\times 10^{-5}\;m^{2}$ tissue region with mesh spacing $\Delta x=\Delta y=5.0\times 10^{-5}\;m$. To ensure compatibility between the ADI solver and the explicit chemotaxis scheme, the time step Δt is chosen to satisfy the CFL condition (13).

Coupling between PDEs and agents is achieved through a global time-stepping loop. Within each global iteration, PDEs are advanced with step Δt for *m* substeps until reaching the agent-based update interval $\Delta t^{\prime }=m\Delta t$. At this point, agents sample local PDE fields and update motility, proliferation, mutation, branching, and anastomosis. Source and sink terms are then passed back to the PDEs, closing the coupling cycle. This multiscale update procedure reflects the faster evolution of biochemical fields relative to discrete cellular events, while maintaining accuracy and consistency across scales.

We discretize the endothelial cell equation using central differences in space and forward Euler in time:

$$\begin{array}{c} \frac{\partial n}{\partial t}=D_{n}\Delta n-\nabla \cdot \left(\chi (c)n\nabla c\right), \end{array}$$

using central differences:

$$\begin{array}{cc} \frac{\partial n}{\partial t} & \approx \frac{n_{i,j}^{k+1}-n_{i,j}^{k}}{\Delta t},\\ D_{n}\Delta n & \approx D_{n}\frac{n_{i-1,j}^{k}+n_{i+1,j}^{k}+n_{i,j-1}^{k}+n_{i,j+1}^{k}-4n_{i,j}^{k}}{\Delta x^{2}}. \end{array}$$

The nonlinear chemotaxis term −∇·(χ(c)n∇c) is discretized using a probabilistic finite difference scheme inspired by hybrid discrete–continuum models [25,40,52]. In this approach, the directional bias of cell motion is encoded as movement probabilities within a von Neumann neighborhood, and the divergence is then approximated by finite differences through fluxes at half indices:

$$\begin{array}{cc} \nabla \cdot (\chi n\nabla c) & \approx \frac{F_{i+\frac{1}{2},j}-F_{i-\frac{1}{2},j}}{\Delta x}+\frac{G_{i,j+\frac{1}{2}}-G_{i,j-\frac{1}{2}}}{\Delta x}, \end{array}$$

with

$$\begin{array}{cc} F_{i+\frac{1}{2},j}:= & \chi \left(c_{i+\frac{1}{2},j}\right)n_{i+\frac{1}{2},j}\frac{c_{i+1,j}-c_{i,j}}{\Delta x}\\ F_{i-\frac{1}{2},j}:= & \chi \left(c_{i-\frac{1}{2},j}\right)n_{i-\frac{1}{2},j}\frac{c_{i,j}-c_{i-1,j}}{\Delta x}\\ G_{i,j+\frac{1}{2}}:= & \chi \left(c_{i,j+\frac{1}{2}}\right)n_{i,j+\frac{1}{2}}\frac{c_{i,j+1}-c_{i,j}}{\Delta x}\\ G_{i,j-\frac{1}{2}}:= & \chi \left(c_{i,j-\frac{1}{2}}\right)n_{i,j-\frac{1}{2}}\frac{c_{i,j}-c_{i,j-1}}{\Delta x} \end{array}$$

with half-index values approximated by linear interpolation, for example:

$$\begin{array}{c} \chi \left(c_{i+\frac{1}{2},j}\right)=\frac{\chi \left(c_{i+1,j}\right)+\chi \left(c_{i,j}\right)}{2},\;n_{i+\frac{1}{2},j}=\frac{n_{i+1,j}+n_{i,j}}{2}. \end{array}$$

The same approach applies to calculating the values of $\chi \left(c_{i-\frac{1}{2},j}\right),\chi \left(c_{i,j+\frac{1}{2}}\right),\chi \left(c_{i,j-\frac{1}{2}}\right)$ and $n_{i-\frac{1}{2},j},n_{i,j+\frac{1}{2}},n_{i,j-\frac{1}{2}}$ in the remaining directions. Substituting the flux expressions yields the discrete update

(14) $$\begin{array}{c} n_{i,j}^{k+1}=n_{i,j}^{k}+\Delta t\left(\frac{D_{n}}{\Delta x^{2}}\delta^{2}n_{i,j}^{k}-\frac{1}{\Delta x}\left(\delta F_{i,j}+\delta G_{i,j}\right)\right) \end{array}$$

Prior analysis by Wang et al. [51] establishes consistency, stability, convergence, nonnegativity, and mass conservation for this explicit chemotaxis discretization, which we summarize below.

**Theorem** **1.**

*(i)*  *Consistency. The local truncation errors of the endothelial chemotaxis and ADI schemes satisfy*
$$\begin{array}{c} \tau_{\mathrm{endo}}=O\left(\Delta t+h^{2}\right),\;\tau_{\mathrm{ADI}}=O\left(\Delta t^{2}+h^{2}\right),\;\mathrm{for}\;\;\Delta x=\Delta y=h. \end{array}$$
*(ii)*  *Stability. The ADI scheme is unconditionally stable for pure linear diffusion. The finite difference scheme for endothelial chemotaxis is conditionally stable under the CFL condition (Equation (* 13 *)). Moreover, subject to the additional constraints*
(15) $$\begin{array}{cc} \Delta x & \leq 2\;\frac{D_{n}}{{∥\chi \left(c\right)\nabla c∥}_{\infty }},\;\Delta t\leq \frac{1}{4\frac{D_{n}}{\Delta x^{2}}+2\frac{{∥\chi \left(c\right)\nabla c∥}_{\infty }}{\Delta x}}, \end{array}$$
  *all motility probabilities $P_{0},P_{1},P_{2},P_{3},P_{4}$ remain nonnegative.*
*(iii)*  *Convergence. Let n(x,y,t) be the exact solution of the endothelial chemotaxis equation, and let $n_{i,j}^{k}$ be its numerical approximation. Under the CFL constraints (Equations (* 13 *) and (* 15 *)), and assuming sufficient regularity of n and c, the scheme satisfies*
$$\begin{array}{c} ∥n_{i,j}^{k}-n\left(x_{i},y_{j},t_{k}\right)∥=O\left(\Delta t+\Delta x^{2}\right). \end{array}$$
*(iv)*  *Nonnegativity. With nonnegative initial data and the CFL conditions (Equations (* 13 *) and (* 15 *)), the finite difference update preserves nonnegativity of $n_{i,j}^{k}$ at each time step.*
*(v)*  *Mass Conservation. With one-sided Neumann boundary approximation, the scheme conserves the total mass:*
$$\begin{array}{c} \sum_{i,j}\;n_{i,j}^{k+1}=\sum_{i,j}\;n_{i,j}^{k}. \end{array}$$

**Figure 8:**
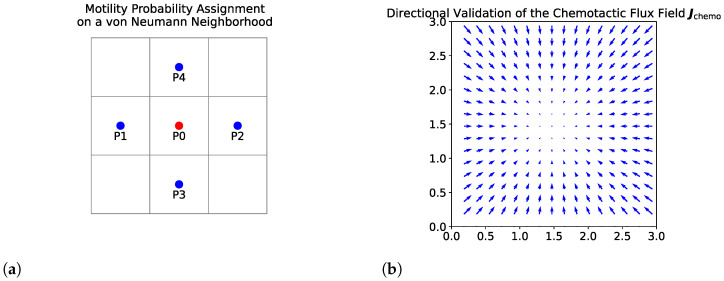
Motility probability structure and directional validation of chemotactic flux. (**a**) The Von Neumann neighborhood and associated movement probabilities. Schematic of Von Neumann neighborhood used for endothelial cell movement. The central red dot indicates the current cell position, which is associated with the probability of remaining stationary $P_{0}$. The four adjacent blue dots correspond to the Von Neumann neighborhoods: left ($P_{1}$), right ($P_{2}$), down ($P_{3}$), and up ($P_{4}$). We calculate movement probabilities based on chemotactic and diffusive cues. This figure uses the same neighborhood structure shown in Figure 3, but labels each neighbor direction with its corresponding motility probability. (**b**) Validating that the chemotactic flux field points toward the source, consistent with the model in Equation (1). Visualization of the chemotactic flux field $J_{\mathrm{chemo}}=\chi \left(c\right)n\nabla c$. Under a tumor-derived TAF concentration $c\left(x,y\right)=e^{-0.05({\left(x-1.5\right)}^{2}+{\left(y-1.5\right)}^{2})}$ over the unit square domain $U^{\prime }={\left[0,3\right]}^{2}$ (1 unit = 5 mm). We evaluate the flux assuming constant cell density n≡1 and the chemotactic sensitivity $\chi \left(c\right)=\chi_{0}/\left(1+\alpha \;c\right)$ with parameter values taken from the authoritative set in Table 3; see also the machine-readable files params.json and params.csv in the GitHub Repository in the Data Availability Statement Section. Flux vectors are oriented along ∇c with arrow lengths proportional to ∥χ(c)∇c∥, exhibiting chemotactic drift toward the TAF peak at (1.5,1.5). The alignment confirms the directional correctness of the flux discretization and its consistency with Equation (1).

To interpret the update probabilistically, we recast the finite difference scheme as a weighted sum of von Neumann neighborhood contributions:

(16) $$\begin{array}{c} n_{i,j}^{k+1}=n_{i,j}^{k}P_{0}+n_{i+1,j}^{k}P_{1}+n_{i-1,j}^{k}P_{2}+n_{i,j+1}^{k}P_{3}+n_{i,j-1}^{k}P_{4}, \end{array}$$

where $P_{0}$ is the probability of remaining stationary and $P_{1}$–$P_{4}$ correspond to movement into the four von Neumann neighbors (Figure 8a).

We derive the movement probabilities as

(17) $$\left\{\begin{array}{cc} P_{0}=1-4\frac{D_{n}\Delta t}{\Delta x^{2}} & -\frac{\Delta t\chi_{0}}{4\Delta x^{2}}\left(\frac{1}{1+\alpha c_{i+1,j}}+\frac{1}{1+\alpha c_{i,j}}\right)\left(c_{i+1,j}-c_{i,j}\right)\\ & +\frac{\Delta t\chi_{0}}{4\Delta x^{2}}\left(\frac{1}{1+\alpha c_{i-1,j}}+\frac{1}{1+\alpha c_{i,j}}\right)\left(c_{i,j}-c_{i-1,j}\right)\\ & -\frac{\Delta t\chi_{0}}{4\Delta x^{2}}\left(\frac{1}{1+\alpha c_{i,j+1}}+\frac{1}{1+\alpha c_{i,j}}\right)\left(c_{i,j+1}-c_{i,j}\right)\\ & +\frac{\Delta t\chi_{0}}{4\Delta x^{2}}\left(\frac{1}{1+\alpha c_{i,j-1}}+\frac{1}{1+\alpha c_{i,j}}\right)\left(c_{i,j}-c_{i,j-1}\right)\\ P_{1}=\frac{D_{n}\Delta t}{\Delta x^{2}}\; & -\frac{\Delta t\chi_{0}}{4\Delta x^{2}}\left(\frac{1}{1+\alpha c_{i+1,j}}+\frac{1}{1+\alpha c_{i,j}}\right)\left(c_{i+1,j}-c_{i,j}\right)\\ P_{2}=\frac{D_{n}\Delta t}{\Delta x^{2}}\; & +\frac{\Delta t\chi_{0}}{4\Delta x^{2}}\left(\frac{1}{1+\alpha c_{i-1,j}}+\frac{1}{1+\alpha c_{i,j}}\right)\left(c_{i,j}-c_{i-1,j}\right)\\ P_{3}=\frac{D_{n}\Delta t}{\Delta x^{2}}\; & -\frac{\Delta t\chi_{0}}{4\Delta x^{2}}\left(\frac{1}{1+\alpha c_{i,j+1}}+\frac{1}{1+\alpha c_{i,j}}\right)\left(c_{i,j+1}-c_{i,j}\right)\\ P_{4}=\frac{D_{n}\Delta t}{\Delta x^{2}}\; & +\frac{\Delta t\chi_{0}}{4\Delta x^{2}}\left(\frac{1}{1+\alpha c_{i,j-1}}+\frac{1}{1+\alpha c_{i,j}}\right)\left(c_{i,j}-c_{i,j-1}\right) \end{array}\right.$$

with chemotactic sensitivity modeled as $\chi \left(c\right)=\chi_{0}/\left(1+\alpha c\right)$. These coefficients combine symmetric diffusion with chemotactic bias from centered differences, producing net movement up TAF gradients.

Raw weights are clipped to remain nonnegative,

$$\begin{array}{c} P_{i}=\max\left\{P_{i},0\right\}, \end{array}$$

and then normalized so that they sum to 1:

$$\begin{array}{c} \sum_{i}P_{i}=1. \end{array}$$

Under CFL conditions (Equations (13) and (15)), positivity holds automatically and only normalization is needed.

Cell movement is sampled stochastically: a random number r∼Uniform[0,1] is compared against cumulative probabilities

(18) $$\begin{array}{c} \begin{array}{cc} R_{0} & =[0,P_{0}],\\ R_{j} & =\left(\sum_{i=0}^{j-1}P_{i},\;\sum_{i=0}^{j}P_{i}\right],\;j=1,\ldots ,4, \end{array} \end{array}$$

and the cell moves in the direction associated with the interval containing *r* [25,40,52]. This construction ensures conservation of total cell number and a complete probabilistic partition of the unit interval.

Finally, to validate the directional behavior of the chemotactic flux $J_{\mathrm{chemo}}=\chi \left(c\right)n\nabla c$ in Equation (1), we visualized the flux field under a representative tumor-derived TAF profile, $c\left(x,y\right)=e^{-0.05({\left(x-1.5\right)}^{2}+{\left(y-1.5\right)}^{2})}$. As shown in Figure 8b, the flux vectors align with the gradient of c(x,y) and consistently point toward the chemotactic source, confirming the correct implementation of the discretized flux term and directional consistency under tumor-induced TAF gradients.

To solve the diffusion-dominated PDEs for molecular fields (Equations (2)–(4)), we employ the ADI method. At each time step, two tridiagonal systems are solved sequentially: first implicit in *x* and explicit in *y*, then reversed. Reaction terms are evaluated explicitly.

For a generic reaction–diffusion equation

$$\begin{array}{c} \frac{\partial u}{\partial t}=D\Delta u+f\left(u,x,y,t\right), \end{array}$$

the ADI discretization reads

(19) $$\begin{array}{cc} \left(1-\frac{D\Delta t}{2\Delta x^{2}}\delta_{x}^{2}\right)U^{k+1/2} & =\left(1+\frac{D\Delta t}{2\Delta y^{2}}\delta_{y}^{2}\right)U^{k}+\frac{\Delta t}{2}f\left(U^{k}\right),\\ \left(1-\frac{D\Delta t}{2\Delta y^{2}}\delta_{y}^{2}\right)U^{k+1} & =\left(1+\frac{D\Delta t}{2\Delta x^{2}}\delta_{x}^{2}\right)U^{k+1/2}+\frac{\Delta t}{2}f\left(U^{k}\right), \end{array}$$

where $U^{k+1/2}\approx u\left(\left(k+1/2\right)\Delta t\right)$. Here, $\delta_{x}^{2}$ and $\delta_{y}^{2}$ denote second-order central difference operators.

This Douglas ADI scheme [24] is unconditionally stable for pure diffusion and computationally efficient for reaction–diffusion systems. In practice, this stability permits large time steps, but to ensure consistency with the explicit chemotaxis update, we restrict Δt to satisfy the CFL conditions (Equations (13) and (15)). This hybrid implicit–explicit strategy balances stability, accuracy, and efficiency in simulating the coupled dynamics.

We couple PDE and ABM updates in a global time-stepping loop. At each global iteration, we advance PDEs for *m* substeps of size Δt until reaching the ABM step $\Delta t^{\prime }=m\Delta t$. Agents then sample local fields, update motility, proliferation, mutation, branching, and anastomosis, and update PDE source and sink terms. The global hybrid solver flowchart (Figure 4), PDE update algorithm (Algorithm A1), and ABM update algorithm (Algorithm A2) contain full implementation details.

## 4. Mathematical Analysis

Recent advances in stochastic population models have established rigorous frameworks for demographic noise [53]. Inspired by such approaches, we extend stability and bifurcation analysis to the tumor angiogenesis context, where spatial heterogeneity and chemotaxis play key roles.

### 4.1. Unidirectional Coupling

In our baseline model (Equation (6)–(9)), endothelial cells chemotactically migrate along TAF gradients but do not secrete TAF; only hypoxic tumors act as TAF sources. We refer to this setting as unidirectional coupling: endothelial cells chemotactically migrate along TAF gradients but do not secrete TAF. Biologically, this reflects the canonical pathway in which hypoxia induces VEGF secretion via hypoxia-inducible factor-1α (HIF-1α) [10], and vessels mitigate hypoxia by restoring oxygen supply. The resulting negative feedback, where endothelial chemotaxis reduces TAF secretion, suggests a stabilizing effect that suppresses spatial heterogeneity.

To formalize this intuition, we consider the tumor-oxygen-endothelial-TAF system:

(20) $$\begin{array}{c} \left\{\begin{array}{c} \partial_{t}m=D_{m}\Delta m+\alpha_{n}m\left(1-m/m_{\max}\right)H\left(o-o_{\mathrm{hyp}}\right),\\ \partial_{t}o=D_{o}\Delta o-\xi_{o}o-\rho_{o}m+S_{o}\left(1-o\right)n,\\ \partial_{t}n=D_{n}\Delta n-\nabla \cdot \left(\chi \left(c\right)n\nabla c\right),\\ \partial_{t}c=D_{c}\Delta c+\eta mH\left(o_{\mathrm{hyp}}-o\right)-\xi_{c}c-\lambda cn. \end{array}\right. \end{array}$$

where *m* denotes the continuum tumor density field, *o* oxygen concentration, *n* endothelial density, and *c* TAF concentration. Tumor growth follows logistic kinetics with carrying capacity $m_{\max}$ and proliferation rate $\alpha_{n}$. The Heaviside functions $H(o-o_{\mathrm{hyp}})$ and $H(o_{\mathrm{hyp}}-o)$ enforce oxygen-threshold-dependent switching of proliferation and TAF secretion, respectively.

On the other hand, in the simplified formulation Equation (20), we approximate tumor density by a continuous logistic field m(x,t). We couple TAF production to hypoxia through a Heaviside switch $H(o_{\mathrm{hyp}}-o)$, which activates secretion when oxygen concentration falls below the hypoxia threshold $o_{\mathrm{hyp}}$. This binary switch simplifies the analysis and facilitates linearization around steady states. We note, however, that in reality, VEGF secretion responds gradually to decreasing oxygen levels rather than switching abruptly [54]. Thus, the Heaviside approximation is a modeling simplification. While a sharp cutoff suffices for our illustrative bifurcation analysis, future extensions could incorporate gradual hypoxia-VEGF coupling. In simulations the full model (Equation (20)) can use smooth kinetics to better reflect biological realism, and the Heaviside is only for analytical tractability.

**Theorem** **2** (Pattern Suppression under Unidirectional Coupling). *In the unidirectional tumor-oxygen-endothelial-TAF system (20), where only tumor cells secrete TAF and endothelial cells respond chemotactically but do not produce TAF, no spatial pattern emerges.*

**Proof** **(Sketch).** Linearizing around both normoxic ($c_{0}=0$) and hypoxic ($c_{0}>0$) equilibria yields dispersion relations with eigenvalues which have negative real parts. In both cases, the Routh–Hurwitz conditions are satisfied, so perturbations decay. Full derivations are given in Appendix F.  □

We note that this analysis assumes the linear saturation law for oxygen supply. In Appendix G, we repeat the argument under a Michaelis–Menten supply law and obtain the same conclusion: both the normoxic and hypoxic steady states remain linearly stable, and all nontrivial perturbations decay. Thus, the suppression of spatial patterns under unidirectional coupling is not an artifact of the linear approximation but persists under the more realistic nonlinear kinetics.

This analysis crucially relies on the assumption of unidirectional coupling. Endothelial cells chemotactically migrate along TAF gradients but do not secrete TAF. This kind of unidirectional chemotaxis model (cells responding to fixed signals without self-production) is known to be stable [55]. In contrast, if endothelial cells were allowed to secrete or amplify TAF (bidirectional coupling), the feedback structure would change. Such bidirectional signaling may give rise to chemotactic instabilities (see Section 4.2 and related discussion). This distinction is essential for interpreting both our results and comparisons with the literature.

### 4.2. Bidirectional Coupling

The preceding analysis established that unidirectional coupling does not produce Turing instabilities. In contrast, clinical observations of the tumor microenvironment often reveal the coexistence of vascularized and hypoxic regions. To reconcile this discrepancy, we introduce a reduced endothelial-chemoattractant (*n*-*c*) subsystem that incorporates bidirectional coupling. For clarity, we define bidirectional coupling as the case in which endothelial cells chemotactically migrate along TAF gradients and secrete TAF simultaneously.

Tumor-associated endothelial cells secrete TAF such as angiopoietin-1 (Ang-1), which acts synergistically with VEGFs to amplify angiogenesis [56,57,58]. In addition, autocrine VEGF production by endothelial cells has been documented in human placental endothelium, where VEGF release strongly correlates with sprouting activity in vitro [59]. Additional evidence of autocrine VEGF production can be found therein. Furthermore, tumor-derived microvesicles carrying activated oncogenic receptors can upregulate endothelial VEGF expression, shifting them toward an autocrine angiogenic phenotype within the tumor microenvironment [60]. Collectively, these findings indicate that endothelial cells are not passive responders to tumor-secreted cues. Instead, they can actively sustain and amplify angiogenesis through both paracrine and autocrine pathways.

To capture this feedback, we extend the endothelial–TAF pair in Equation (20) by allowing endothelial cells to produce chemoattractant at a rate $\eta_{n}\geq 0$. The resulting system is

(21) $$\begin{array}{c} \left\{\begin{array}{c} \partial_{t}n=D_{n}\Delta n-\nabla \cdot \left(\chi \left(c\right)n\nabla c\right),\\ \partial_{t}c=D_{c}\Delta c+\eta_{n}n-\xi_{c}c, \end{array}\right. \end{array}$$

with homogeneous Neumann boundary conditions. Here, $D_{n},D_{c}>0$ are diffusion coefficients, $\xi_{c}>0$ is the clearance rate of *c*, and $\chi \left(c\right)=\chi_{0}/\left(1+\alpha c\right)$ is the chemotactic sensitivity. We use $\eta_{n}$ to denote the TAF production rate by endothelial cells, and distinguish it from η, which denotes the TAF production rate by tumor cells. Setting $\eta_{n}=0$ recovers the unidirectional coupling mechanism. The bidirectional coupling scenario contains a self-reinforcing feedback loop:

$$\begin{array}{cc} \cdots \overset{\chi (c)\nabla c}{⟶} & n\;\mathrm{aggregation}\overset{\eta_{n}c}{⟶}c\;\sec\mathrm{retion}\overset{\chi (c)\nabla c}{⟶}\\ \; & \mathrm{enhanced}\;n\;\mathrm{migration}\overset{\eta_{n}c}{⟶}\mathrm{enhanced}\;c\;\sec\mathrm{retion}\overset{\chi (c)\nabla c}{⟶}\cdots \end{array}$$

In this section, we assume uniform baseline densities $n_{0}$, $c_{0}$ set by tumor production and vessel uptake, and focus on perturbations of n,c. We emphasize that the analysis of the reduced *n*-*c* subsystem captures the essence of the patterning mechanism, and acknowledge that the other fields (oxygen, drug, tumor) remain near uniform.

**Theorem** **3** (Pattern Formation under Bidirectional Coupling). *The system Equation (* 21 *) admits a spatially homogeneous steady state $\left(n_{0},c_{0}\right)$ with*

$$\begin{array}{c} \eta_{n}n_{0}-\xi_{c}c_{0}=0. \end{array}$$

*Linearizing around this steady state $\left(n,c\right)=\left(n_{0}+\tilde{n},c_{0}+\tilde{c}\right)$ yields the following system:*

$$\begin{array}{c} \left\{\begin{array}{c} \partial_{t}\tilde{n}=D_{n}\Delta \tilde{n}-\chi_{\mathrm{eff}}n_{0}\Delta \tilde{c},\\ \partial_{t}\tilde{c}=D_{c}\Delta \tilde{c}+\eta_{n}\tilde{n}-\xi_{c}\tilde{c}. \end{array}\right. \end{array}$$

*Here,*

$$\begin{array}{c} \chi_{\mathrm{eff}}\chi_{0}/\left(1+\alpha c_{0}\right) \end{array}$$

*is the effective chemotaxis sensitivity parameter at the steady state $\left(n_{0},c_{0}\right)$. We expand the small perturbations with respect to each spectral pair of the Neumann Laplacian:*

$$\begin{array}{c} \Delta \phi_{j}+\lambda_{j}\phi_{j}=0\;\mathrm{on}\;U,\;\nabla_{\vec{n}}\phi_{j}=0\;\mathrm{on}\;\partial U, \end{array}$$

*where $\vec{n}$ is the unit outward normal vector on ∂U. This expansion yields the following dispersion relation:*

$$\begin{array}{c} \sigma^{2}+a_{1}\left(\lambda_{j}\right)\sigma +a_{0}\left(\lambda_{j}\right)=0, \end{array}$$

*with*

$$\begin{array}{c} a_{1}\left(\lambda_{j}\right)=\left(D_{n}+D_{c}\right)\lambda_{j}+\xi_{c},\;a_{0}\left(\lambda_{j}\right)=D_{n}D_{c}\lambda_{j}^{2}+\left(D_{n}\xi_{c}-\eta_{n}\chi_{\mathrm{eff}}n_{0}\right)\lambda_{j}. \end{array}$$

*Turing instability requires*

$$\begin{array}{c} D_{n}D_{c}\lambda_{j}^{2}+\left(D_{n}\xi_{c}-\eta_{n}\chi_{\mathrm{eff}}n_{0}\right)\lambda_{j}<0 \end{array}$$

*for some $\lambda_{j}$. Only when*

$$\begin{array}{c} D_{n}\xi_{c}<\eta_{n}\chi_{\mathrm{eff}}n_{0}, \end{array}$$

*linear instability arises if the squared wavenumber $\lambda_{j}$ satisfies the following:*

$$\begin{array}{c} {\underline{\lambda }}_{j}=0<\lambda_{j}<\frac{\eta_{n}\chi_{\mathrm{eff}}n_{0}-D_{n}\xi_{c}}{D_{n}D_{c}}{\bar{\lambda }}_{j}. \end{array}$$

*In a finite-domain U, the admissible wavenumbers are discrete, with the maximum constrained by ${\bar{\lambda }}_{j}=\frac{\eta_{n}\chi_{\mathrm{eff}}n_{0}-D_{n}\xi_{c}}{D_{n}D_{c}}$.*

*Furthermore, $a_{1}\left(\lambda_{j}\right)>0$, precluding a Hopf bifurcation. More explicitly, since $a_{1}>0$ and $a_{0}<0$ at instability, the eigenvalues are real and one becomes positive, so patterns are stationary (no oscillatory eigenvalues). Complex conjugate roots, when present, are strictly damped. Hence, the only linear route to pattern formation is the stationary Turing band $a_{0}\left(\lambda_{j}\right)<0$.*

**Proof** **(Sketch).** Linearization of the bidirectional system about the homogeneous equilibrium $\left(n_{0},c_{0}\right)$ leads to a quadratic dispersion relation. Instability occurs exactly when $D_{n}\xi_{c}<\eta_{n}\chi_{\mathrm{eff}}n_{0}$, in which case a band of unstable wavenumbers exists and stationary Turing patterns form. Otherwise all perturbations decay. Full algebraic details are provided in Appendix F.  □

**Remark** **2.**

(i) *All results in Section 4.1 and Section 4.2 are derived from linearization around homogeneous steady states. They determine conditions for the onset of instability but do not address nonlinear dynamics (e.g., pattern selection), which remain open questions for future work.*
(ii) *Our analysis is in a two-dimensional cross-section, which necessarily omits true three-dimensional features such as vascular tortuosity and branching geometry. By Weyl’s law [61], the eigenvalue count satisfies*
$$\begin{array}{c} N\left(\lambda \right)\propto \mathrm{vol}\left(\Omega \right)\;\lambda^{d/2}, \end{array}$$
  *the number of Laplacian eigenmodes below a threshold grows faster in 3D (d=3) than in 2D (d=2). Thus, three-dimensional geometries generally admit more unstable modes within a given band, and one may expect similar instabilities, potentially with even broader unstable ranges.*
(iii) *An important corollary of Theorem 3 is that the instability band arises only through a Turing instability: since $a_{1}\left(\lambda \right)>0$, complex-conjugate eigenvalues are strictly damped and no Hopf bifurcation occurs. Thus, the model predicts the formation of stable spatial patterns rather than sustained oscillations. This outcome is consistent with classical results on chemotaxis-driven patterning, as reviewed in the Keller–Segel framework [62].*

The feedback loop formalized in (21) is strongly grounded in experimental evidence. Endothelial cells not only respond to exogenous VEGF and other TAFs but also secrete angiogenic factors such as Ang-1 and VEGF themselves, both constitutively and in response to oncogenic signals. This autocrine secretion establishes a self-reinforcing loop: local accumulation of endothelial cells enhances TAF release, which in turn sharpens chemotactic gradients and drives further aggregation. Tumor-derived microvesicles further enhance this positive feedback loop by upregulating endothelial VEGF expression, effectively converting endothelial cells from passive responders into active drivers of angiogenesis. In the model, this dynamic is encapsulated by the parameter $\eta_{n}$, which closes the feedback loop between endothelial density and chemoattractant concentration. From a systems perspective, bidirectional coupling increases the likelihood of runaway positive feedback and lowers the threshold for pattern-forming instabilities. From a biological perspective, it provides a parsimonious explanation for how endothelial cells can collectively sustain and amplify vascular sprouting within the tumor microenvironment. Thus, the inclusion of $\eta_{n}>0$ in our model is not only mathematically consequential but also biologically justified by emerging experimental findings on autocrine endothelial signaling.

## 5. Results

Our analysis predicts that any Fourier mode with squared wavenumber lies in the unstable band $\left(0,{\bar{\lambda }}_{j}\right)$ evolves into spatial patterns. However, we also note that even with the inequality $0<\lambda_{j}<{\bar{\lambda }}_{j}$ satisfied, a finite-domain may prevent patterning. The continuous unstable band $\left(0,{\bar{\lambda }}_{j}\right)$ must contain at least one admissible discrete Neumann eigenvalue of the domain. We demonstrate this constraint below.

### 5.1. Finite-Domain Constraints

Consider the Neumann spectral problem on the rectangle $U=\left[0,L_{a}\right]\times \left[0,L_{b}\right]$. The Neumann eigenpairs take the form

$$\begin{array}{cc} \phi_{p,q} & =c_{p,q}\cos\left(\frac{p\pi x}{L_{a}}\right)\cos\left(\frac{q\pi y}{L_{b}}\right),\\ \lambda_{p,q} & ={\left(\frac{p\pi }{L_{a}}\right)}^{2}+{\left(\frac{q\pi }{L_{b}}\right)}^{2}, \end{array}$$

for $\left(p,q\right)\in Z^{2}$. The dispersion relation reads

$$\begin{array}{c} \sigma^{2}+a_{1}\left(\lambda_{p,q}\right)\sigma +a_{0}\left(\lambda_{p,q}\right)=0, \end{array}$$

with $a_{1}\left(\lambda_{p,q}\right)=\left(D_{n}+D_{c}\right)\lambda_{p,q}+\xi_{c}$ and $a_{0}\left(\lambda_{p,q}\right)=D_{n}D_{c}\lambda_{p,q}^{2}+\left(D_{n}\xi_{c}-\eta_{n}\chi_{\mathrm{eff}}n_{0}\right)\lambda_{p,q}$. The continuous unstable band reads $\left(0,{\bar{\lambda }}_{p,q}\right)$ where ${\bar{\lambda }}_{p,q}=\frac{\eta_{n}\chi_{\mathrm{eff}}n_{0}-D_{n}\xi_{c}}{D_{n}D_{c}}$. Assume $L_{a}\leq L_{b}$. Patterns occur if and only if at least one admissible $\lambda_{p,q}$ lies in $\left(0,{\bar{\lambda }}_{p,q}\right)$. The first nontrivial Neumann mode has squared wavenumber $\pi^{2}/L_{b}^{2}$, so the necessary and sufficient condition for patterning reads

$$\begin{array}{c} \frac{\pi^{2}}{L_{b}^{2}}<{\bar{\lambda }}_{p,q}\Rightarrow L_{b}>\frac{\pi }{\sqrt{{\bar{\lambda }}_{p,q}}}. \end{array}$$

Note that $L_{b}>\frac{\pi }{\sqrt{{\bar{\lambda }}_{p,q}}}$ only guarantees that the first nontrivial Neumann mode (with p=0,q=1) lies in the unstable band. The domain must exceed a critical linear size to admit any unstable Neumann mode.

Next, we evaluate the critical length using the parameter set in Table 3. We vary the endothelial-chemoattractant production rate $\eta_{n}$ across orders of magnitude and set $n_{0}=1$, $D_{n}=4.61\times 10^{-4}$, $D_{c}=0.12$, α=0.6, $\xi_{c}=0.002$, $\chi_{0}=0.0599$, and $L_{a}=L_{b}=5$. The effective chemotactic sensitivity equals $\chi_{\mathrm{eff}}=\frac{\chi_{0}}{1+\alpha c_{0}}=\frac{\chi_{0}}{1+\alpha \eta_{n}n_{0}/\xi_{c}}$. Table 6 lists the computed values of ${\bar{\lambda }}_{p,q}$ and the critical length $L_{\mathrm{crit}}$ for $\eta_{n}\in \left\{0.001,0.01,0.05,0.1,0.5,1\right\}$. Increasing $\eta_{n}$ expands the unstable band $\left(0,{\bar{\lambda }}_{p,q}\right)$ and reduces the minimal domain $L_{\mathrm{crit}}$ that supports patterning.

Table 7 reports the first nine Neumann modes on ${\left[0,5\right]}^{2}$ and marks which modes satisfy $\lambda_{p,q}<\bar{\lambda }$ for the $\eta_{n}$ values above. The trivial uniform mode (0,0) with $\lambda_{0,0}=0$ does not participate in Turing instability.

Using the instability condition $D_{n}\xi_{c}<\eta_{n}\chi_{\mathrm{eff}}n_{0}$ together with $\chi_{\mathrm{eff}}=\frac{\chi_{0}}{1+\alpha \eta_{n}n_{0}/\xi_{c}}$, we obtain the explicit lower bound

$$\begin{array}{c} \eta_{n}>\frac{D_{n}\xi_{c}}{\left(\chi_{0}-D_{n}\alpha \right)n_{0}}≕\eta_{n}^{0}. \end{array}$$

With the parameter value above and $n_{0}=1$ we compute $\eta_{n}^{0}=2.4281\times 10^{-6}$. Therefore, any net endothelial cell to TAF production comparable to or larger than $2.43\times 10^{-6}$ crosses the linear threshold that permits a Turing chemotactic band, though a finite-domain must still contain an admissible mode.

Throughout, we assumed homogeneous Neumann (no-flux) boundaries, consistent with modeling tumor tissue bounded by impermeable surroundings. For completeness and comparison, we also consider periodic boundaries, which are widely used to emulate a toroidal domain and thereby approximate an effectively infinite tissue without edge effects. The smallest nonzero periodic mode has squared wavenumber ${\left(2\pi /L_{b}\right)}^{2}$. Hence, periodic boundaries alter the minimal admissible wavelength and change the finite-domain threshold to

$$\begin{array}{c} \frac{4\pi^{2}}{L_{b}^{2}}<{\bar{\lambda }}_{p,q}\Rightarrow L_{b}>\frac{2\pi }{\sqrt{{\bar{\lambda }}_{p,q}}}, \end{array}$$

which explains the factor-of-two difference in critical linear dimension relative to Neumann boundaries. Appendix H gives further details.

**Remark** **3** (Boundary Conditions). *The choice of boundary condition alters the smallest admissible eigenvalue: under Neumann BCs it is $\pi^{2}/L^{2}$, while under periodic BCs it is ${\left(2\pi \right)}^{2}/L^{2}$. Consequently, the critical domain size differs by a factor-of-two,*

$$\begin{array}{c} L_{\mathrm{crit}}^{\mathrm{Neumann}}=\frac{\pi }{\sqrt{\bar{\lambda }}},\;L_{\mathrm{crit}}^{\mathrm{Periodic}}=\frac{2\pi }{\sqrt{\bar{\lambda }}}. \end{array}$$

*We explicitly note this distinction to clarify that the instability threshold depends not only on system parameters but also on boundary conditions.*

When an unstable mode $e^{\sigma (\lambda_{j})t}\phi_{j}$ exists, the dominate pattern follows the mode with maximal growth rate, i.e., $e^{\sigma (\lambda_{j_{0}})t}\phi_{j_{0}}$, where $ℜ(\sigma \left(\lambda_{j_{0}}\right))$ attains its maximum. We identify the dominant squared wavenumber $\lambda_{j_{0}}$ via

$$\begin{array}{c} j_{0}=\arg\min_{j}|\lambda_{j}-\lambda^{*}{\left|,\;\mathrm{where}\;\;\frac{d\sigma (\lambda )}{d\lambda }\right|}_{\lambda^{*}}=0. \end{array}$$

The wavelength for each wavenumber $\sqrt{\lambda_{j}}$ is $\frac{2\pi }{\sqrt{\lambda_{j}}}$. Therefore, the n−c subsystem Equation (21) predicts spatially periodic patterns with spacing $\frac{2\pi }{\sqrt{\lambda_{j_{0}}}}$.

Thus, the model predicts that beyond a very small threshold of autocrine TAF production by endothelial cells, spatial patterns of vessels will emerge on domains with length exceeding $L_{\mathrm{crit}}$. Using our nondimensional parameters, $L_{\mathrm{crit}}=1.66$, which corresponds to about ≈8.3 mm in physical units.

### 5.2. Bifurcation Diagram

**Figure 9:**
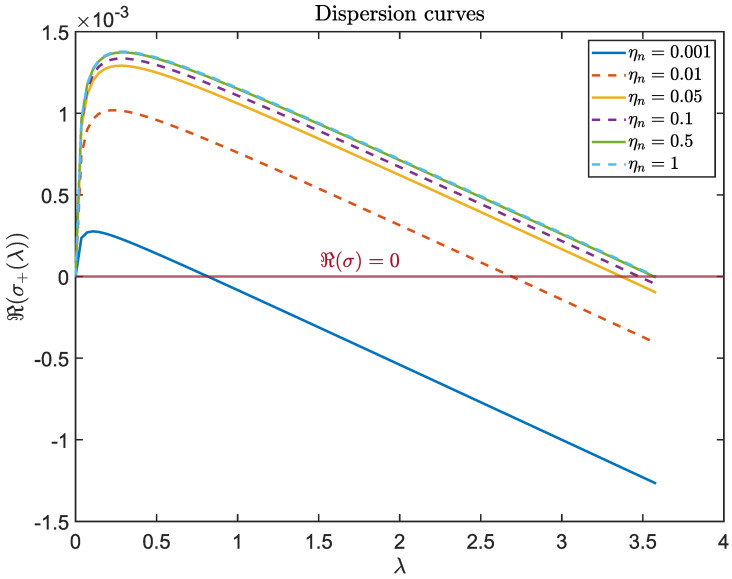
Dispersion curves $ℜ(\sigma_{+}\left(\lambda \right))$ vs. λ across $\eta_{n}\in \left\{0.001,0.01,0.05,0.1,0.5,1\right\}$. All parameter values are taken from the authoritative set in Table 3; see also the machine-readable files params.json and params.csv in the GitHub Repository in the Data Availability Statement Section. The horizontal green line marks the bifurcation threshold ℜ(σ)=0. Its intersection with each dispersion curve determines the critical value $\bar{\lambda }$, beyond which spatial patterns disappear. Larger values of $\eta_{n}$ shift the threshold to higher $\bar{\lambda }$, thereby enlarging the unstable band $\left(0,\bar{\lambda }\right)$ and promoting a wider range of unstable modes.

We fix the square domain $U={\left[0,5\right]}^{2}$ and plot dispersion curves $ℜ(\sigma_{+}\left(\lambda \right))$ versus λ for $\eta_{n}\in \left\{0.001,0.01,0.05,0.1,0.5,1\right\}$ in Figure 9. For each $\eta_{n}$, the dominant eigenvalue $ℜ(\sigma_{+}\left(\lambda \right))$ rises to a local maximum and then falls below zero as λ increases. For fixed λ, larger values of $\eta_{n}$ shift the dispersion curve upward, leading to higher values of $ℜ(\sigma_{+}\left(\lambda \right))$ and thereby reflecting the destabilizing effect of TAF production. The horizontal green solid line marks the zero-eigenvalue threshold ℜ(σ)=0. Its intersection with each curve determines the bifurcation value of λ, beyond which spatial patterns disappear. As $\eta_{n}$ increases, this bifurcation value increases, indicating that stronger TAF production enlarges the unstable band $\left(0,\bar{\lambda }\right)$ and thus promotes a wider range of unstable modes.

**Figure 10:**
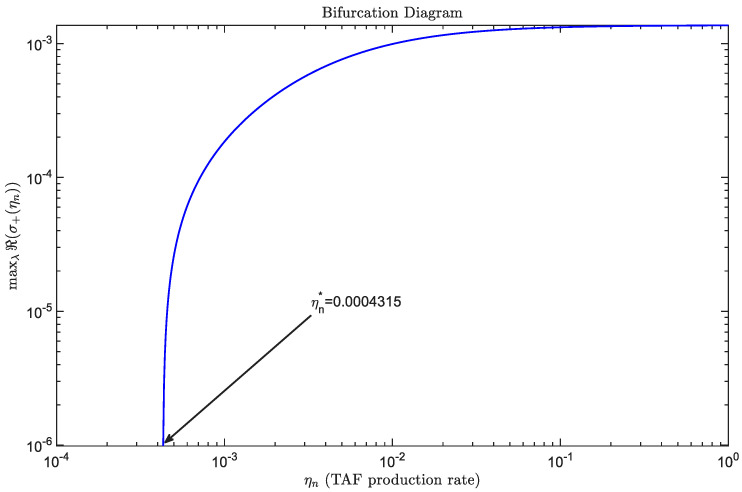
Bifurcation diagram of $\max_{\lambda }ℜ\left(\sigma_{+}\left(\eta_{n}\right)\right)$ vs. $\eta_{n}$. All parameter values are taken from the authoritative set in Table 3; see also the machine-readable files params.json and params.csv in the GitHub Repository in the Data Availability Statement Section. The maximum $\max_{\lambda }\;ℜ\left(\sigma_{+}\left(\eta_{n}\right)\right)$ is taken over all admissible $\lambda \in (0,{\bar{\lambda }}_{2})$. The black arrow indicates the threshold value for $\eta_{n}$ across which spatial patterns start to emerge.

Figure 10 presents the bifurcation diagram of $\max ℜ(\sigma_{+}\left(\eta_{n}\right))$ as a function of $\eta_{n}$. The black arrow highlights the critical bifurcation value of $\eta_{n}$, above which spatial patterns appear. Beyond this threshold, increasing $\eta_{n}$ further amplifies maxℜ(σ). Simulation yields a critical value $\eta_{n}^{*}=0.0004315$, which differs from the theoretical small parameter threshold $2.43\times 10^{-6}$. This discrepancy arises because very small $\eta_{n}$ produce an unstable band that remains too narrow to contain any admissible discrete wavenumber on the chosen domain. In such cases, the maximum $\max_{\lambda }\;ℜ\left(\sigma_{+}\left(\eta_{n}\right)\right)$ is evaluated over an empty set, and we assign it the value zero whenever $\eta_{n}<\eta_{n}^{*}$.

Solving the discrete mode condition $D_{n}\xi_{c}=\eta_{n}\chi_{\mathrm{eff}}n_{0}$ for the smallest nonzero Neumann eigenvalue yields the computational threshold

(22) $$\begin{array}{c} \eta_{n}^{*}=\frac{A\xi_{c}}{\chi_{0}n_{0}\xi_{c}-A\alpha n_{0}},\;A=D_{n}\xi_{c}+\frac{\pi^{2}D_{n}D_{c}}{\max{\left\{L_{a},L_{b}\right\}}^{2}}. \end{array}$$

Substituting the parameter values yields $\eta_{n}^{*}=4.2888\times 10^{-4}$, in close agreement with the simulation threshold reported in Figure 10. Appendix I contains the derivation.

The factor $\pi^{2}/\max{\left\{L_{a},L_{b}\right\}}^{2}$ originates from the smallest nonzero Neumann eigenvalue on the rectangle $U=\left[0,L_{a}\right]\times \left[0,L_{b}\right]$. The Neumann eigenpairs are

$$\begin{array}{c} \phi_{p,q}\left(x,y\right)\propto \cos\;\left(\frac{p\pi x}{L_{a}}\right)\cos\;\left(\frac{q\pi y}{L_{b}}\right),\;\lambda_{p,q}={\left(\frac{p\pi }{L_{a}}\right)}^{2}+{\left(\frac{q\pi }{L_{b}}\right)}^{2}, \end{array}$$

for integers p,q≥0. The trivial spatially uniform mode corresponds to (p,q)=(0,0) with $\lambda_{0,0}=0$. The first nontrivial modes are (1,0) and (0,1), whose squared wavenumbers are $\pi^{2}/L_{a}^{2}$ and $\pi^{2}/L_{b}^{2}$. Thus, the smallest nonzero eigenvalue is

$$\begin{array}{c} \lambda_{\min}=\frac{\pi^{2}}{\max{\left\{L_{a},L_{b}\right\}}^{2}}, \end{array}$$

which is the value we substitute into the instability inequality $\eta_{n}\chi_{\mathrm{eff}}n_{0}>D_{n}\xi_{c}+D_{n}D_{c}\lambda$ to obtain the finite-domain threshold. Equivalently, the discrete eigenindex (p,q) directly encodes the number of half-waves in each spatial direction and therefore links the admissible modes to physical domain lengths $L_{a},L_{b}$. For periodic boundaries, the corresponding minimal nonzero mode is (p,q)=(1,0) or (0,1) but with eigenvalue ${\left(2\pi /L\right)}^{2}$, which explains the factor-of-two difference in the critical linear dimension (Neumann vs. periodic) discussed elsewhere in the text.

We give some practical notes for the reader. Because $\chi_{\mathrm{eff}}=\chi_{0}/\left(1+\alpha c_{0}\right)$ depends implicitly on $\eta_{n}$ through $c_{0}=\eta_{n}n_{0}/\xi_{c}$, the inequality that defines $\eta_{n}^{*}$ is nonlinear in $\eta_{n}$. The form (22) makes the finite-domain dependence explicit and is valid precisely when $\chi_{0}\xi_{c}-A\alpha >0$; we therefore recommend reporting whether this positivity condition holds for the parameter regimes used in any numerical experiment (as done above).

### 5.3. Turing Instability

We validate the theoretical predictions under Neumann boundary conditions by selecting

$$\begin{array}{cc} \; & n_{0}=1,\;D_{n}=4.61\times 10^{-4},\;D_{c}=0.5\times 0.12,\;\alpha =0.6,\\ \; & \xi_{c}=5\times 0.002,\;\chi_{0}=20\times 0.0599,\;L_{a}=4,\;L_{b}=1. \end{array}$$

**Figure 11:**
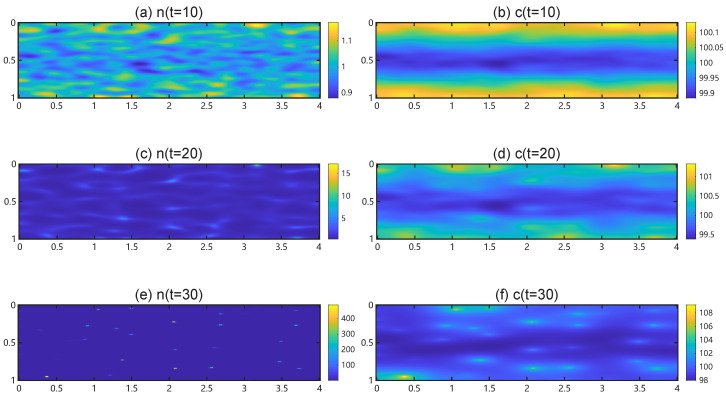
Turing patterns in the coupled *n*–*c* subsystem under Neumann boundary conditions. (**a**,**c**,**e**) Endothelial cell field *n* at t=10,20,30; (**b**,**d**,**f**) TAF field *c* at t=10,20,30. Axes correspond to spatial coordinates x∈[0,4],y∈[0,1]. Colorbars are normalized separately for each variable and time point to emphasize relative heterogeneity and spatial patterning. The endothelial field transitions from stripes to dot-like structures, while the TAF field evolves from a homogeneous state to stripe patterns, consistent with bidirectional coupling and progressive enhancement of spatial heterogeneity.

These parameters yield the critical domain length L=0.1179. Figure 11 shows Turing patterns in the coupled *n*–*c* subsystem. Endothelial density first organizes into elongated stripes and then splits into dot-like clusters as spatial heterogeneity amplifies. In contrast, the TAF field evolves from a nearly homogeneous state to stripes, demonstrating the interplay between the two fields during pattern selection.

**Figure 12:**
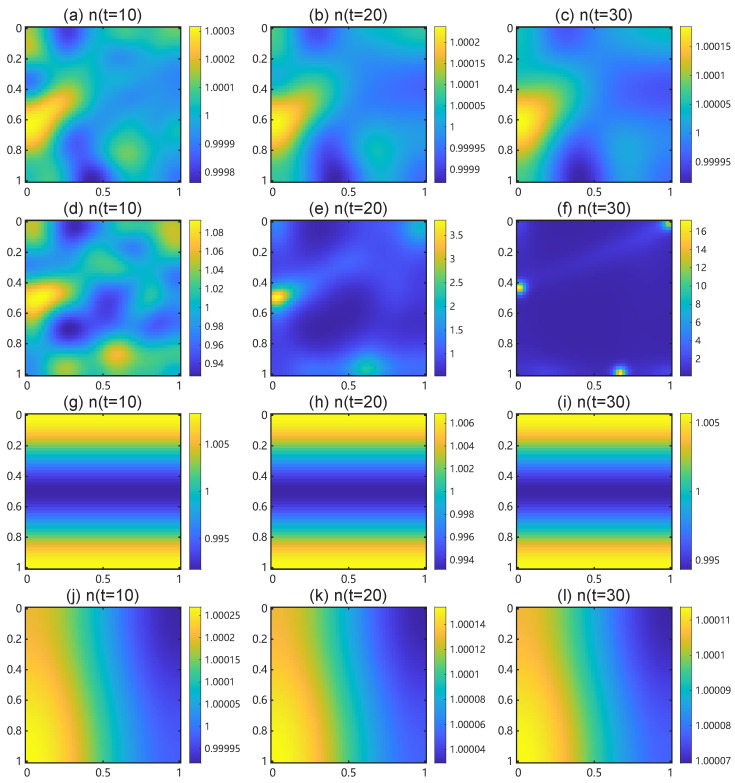
Snapshots of endothelial cell density *n* at t=10,20,30 across the four scenarios. (**a**–**c**) Scenario I; (**d**–**f**) Scenario II; (**g**–**i**) Scenario III; (**j**–**l**) Scenario IV. Axes denote x,y∈[0,1]. Colorbars are normalized per panel per time to highlight heterogeneity. Only Scenario II develops Turing patterns, manifesting as dot-like endothelial clusters.

**Figure 13:**
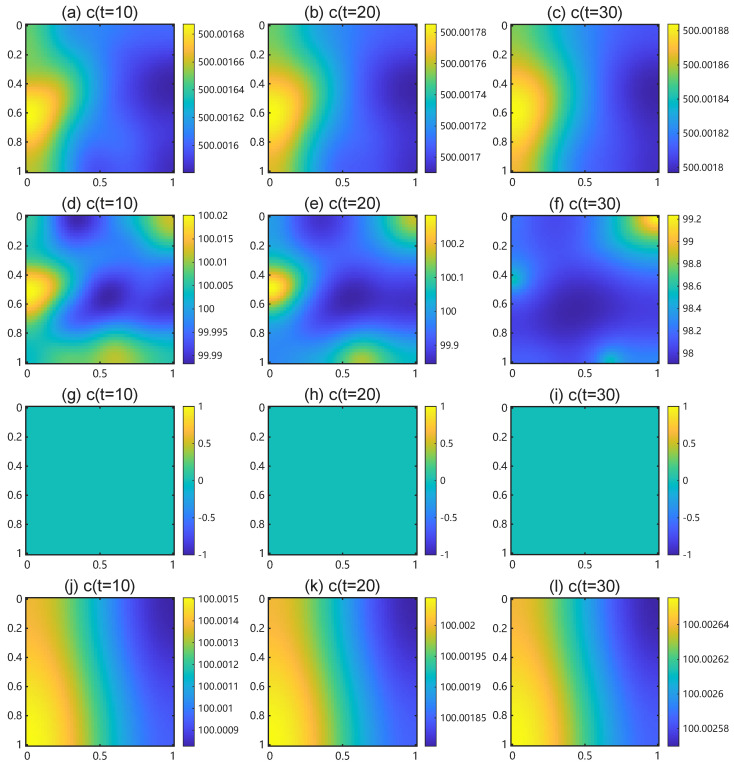
Snapshots of TAF concentration *c* at t=10,20,30 across the four scenarios. (**a**–**c**) Scenario I; (**d**–**f**) Scenario II; (**g**–**i**) Scenario III; (**j**–**l**) Scenario IV. Axes denote x,y∈[0,1]. Colorbars are normalized per panel per time. Consistent with Figure 12, only Scenario II exhibits Turing patterns in the TAF field. In Scenario III, *c* decays to zero due to the absence of endothelial feedback.

We next examine four representative parameter regimes under Neumann boundary conditions. In scenario I, we set

$$\begin{array}{cc} \; & \eta_{n}=1,\;n_{0}=1,\;D_{n}=4.61\times 10^{-4},\;D_{c}=0.12,\;\alpha =0.6,\\ \; & \xi_{c}=0.002,\;\chi_{0}=0.0599,\;L_{a}=1,\;L_{b}=1, \end{array}$$

which gives the critical domain length L=1.6602. Since $L>L_{a}$, the system admits no unstable modes, and patterns do not form (Figure 12a–c and Figure 13a–c). In scenario II, we set

$$\begin{array}{cc} \; & \eta_{n}=1,\;n_{0}=1,\;D_{n}=4.61\times 10^{-4},\;D_{c}=0.5\times 0.12,\;\alpha =0.6,\\ \; & \xi_{c}=5\times 0.002,\;\chi_{0}=20\times 0.0599,\;L_{a}=1,\;L_{b}=1, \end{array}$$

which decreases the critical length to L=0.1179. The reduction in $D_{c}$ and the amplification of $\left(\xi_{c},\chi_{0}\right)$ ensure $L<L_{a}$ and produce discrete unstable modes that generate Turing patterns (Figure 12d–f and Figure 13d–f). In scenario III, we impose unidirectional coupling by choosing

$$\begin{array}{cc} \; & \eta_{n}=0,\;n_{0}=1,\;D_{n}=4.61\times 10^{-4},\;D_{c}=0.5\times 0.12,\;\alpha =0.6,\\ \; & \xi_{c}=5\times 0.002,\;\chi_{0}=20\times 0.0599,\;L_{a}=1,\;L_{b}=1. \end{array}$$

This choice removes endothelial feedback. The system does not sustain Turing patterns, and *c* decays as shown in Figure 12g–i and Figure 13g–i. In Scenario IV, we increase diffusion with

$$\begin{array}{cc} \; & \eta_{n}=1,\;n_{0}=1,\;D_{n}=100\times 4.61\times 10^{-4},\;D_{c}=0.5\times 0.12,\;\alpha =0.6,\\ \; & \xi_{c}=5\times 0.002,\;\chi_{0}=20\times 0.0599,\;L_{a}=1,\;L_{b}=1, \end{array}$$

which yields L=1.1931. Because $L>L_{a}$, unstable modes are not available and patterns do not form (Figure 12j–l and Figure 13j–l).

These comparisons show three principles. First, $D_{c}$, $\xi_{c}$, and $\chi_{0}$ jointly control pattern onset, as Scenarios I and II illustrate. Second, bidirectional coupling is necessary and sufficient for instability, as Scenarios II and III demonstrate. Third, large endothelial diffusion coefficient $D_{n}$ suppresses patterns by enlarging the critical domain length, as Scenarios II and IV confirm. The maximal unstable mode

$$\begin{array}{c} \bar{\lambda }=\frac{\eta_{n}\;\frac{\chi_{0}}{1+\alpha \eta_{n}n_{0}/\xi_{c}}\;n_{0}-D_{n}\xi_{c}}{D_{n}D_{c}}=\xi_{c}\;\frac{\frac{\eta_{n}\chi_{0}n_{0}}{\xi_{c}+\alpha \eta_{n}n_{0}}-D_{n}}{D_{n}D_{c}}. \end{array}$$

does not vary monotonically with $\xi_{c}$. Scenarios I and II show that increasing $\xi_{c}$ can still permit patterns when concurrent changes in $D_{c}$ and $\chi_{0}$ enhance the net instability.

While our hybrid PDE-ABM framework can, in principle, generate explicit spatial simulations (e.g., vessel networks, resistant niches), the present work is methodological and does not attempt to construct a general-purpose simulation platform. Instead, we present dispersion relations, bifurcation diagrams, and Turing patterns under bidirectional coupling both as representative outputs of the framework and as validation of stability analysis. These results demonstrate that the model is capable of producing conditions under which heterogeneous vascular clusters and hypoxic niches may emerge, although we do not visualize full-scale simulations here.

In summary, our mathematical analysis shows that when endothelial cells respond only to TAF, the coupled tumor-oxygen-endothelial-TAF system is linearly stable. Under these conditions, it cannot generate Turing instability. This result highlights a stabilizing effect of unidirectional endothelial-TAF coupling, where vessels relieve hypoxia and thereby suppress further TAF production. However, introducing bidirectional endothelial–chemoattractant coupling, motivated by tumor-associated endothelial cell biology, restores the possibility of chemotactic instability. In this reduced *n*-*c* subsystem, instability arises when endothelial-driven TAF production is sufficiently strong relative to endothelial motility and TAF clearance. Under these conditions, the system develops spatially periodic vessel aggregation. The analysis further reveals that domain size and boundary conditions constrain the admissible unstable modes. Transient oscillations may emerge from damped dynamics, but not from Hopf bifurcations. Together, these results provide a mechanistic explanation for the coexistence of vascularized and hypoxic regions in tumors. Spatial self-organization arises not from canonical tumor-to-vessel signaling alone, but from bidirectional endothelial–TAF coupling. This mechanism is consistent with the universality of chemotactic pattern formation [55]: unidirectional coupling suppresses patterns, while bidirectional coupling restores them.

Biological Interpretations: The instability condition

$$\begin{array}{c} D_{n}\xi_{c}<\eta_{n}\chi_{\mathrm{eff}}n_{0} \end{array}$$

captures the competition between chemotactic aggregation and diffusive or decay damping. High TAF production $\eta_{n}$ and strong chemotactic sensitivity $\chi_{\mathrm{eff}}$ drives aggregation, while large endothelial diffusion $D_{n}$ or rapid TAF clearance $\xi_{c}$ counteract it. When the condition holds, the *n*–*c* subsystem predicts vascular patterns with characteristic spacing

$$\begin{array}{c} \frac{2\pi }{\sqrt{\lambda_{j_{0}}}}, \end{array}$$

set by diffusivities.

Biologically, this implies that even with uniform initial conditions, endothelial cells self-organize into clusters separated by hypoxic regions. Our model therefore predicts the clinically observed high spatial heterogeneity of the tumor microenvironment, where vascularized zones coexist with poorly perfused, hypoxic regions (Figure 1 in [63]). Such heterogeneity yields uneven oxygenation and drug penetration, creating protected niches for resistance persistence [47,64,65]. We further find no sustained oscillations (no Hopf bifurcation), consistent with angiogenic oscillations being transient phenomena [66].

In summary, the analysis predicts that chemotaxis-driven self-organization produces vascular and hypoxic heterogeneity, explaining why tumors often harbor resistant niches. Therapeutically, strategies that tuning TAF clearance $\xi_{c}$, reduce chemotactic sensitivity $\chi_{0}$, or increase random motility $D_{n}$ may suppress such pattern formation and improve perfusion homogeneity.

## 6. Discussion

We demonstrated that bidirectional endothelial–TAF coupling is both necessary and sufficient to generate Turing instability, whereas unidirectional models remain stable. Allowing endothelial cells to secrete chemoattractant transforms a homogeneous vasculature into clusters separated by hypoxic, poorly perfused regions. This mechanism provides a direct link between angiogenic signaling and tumor microenvironmental heterogeneity. By deriving an analytic threshold $\eta_{n}>\eta_{n}^{*}$ and corroborating it numerically, we identify endothelial-derived TAF production as the principal driver of instability.

Our results provide a new theoretical perspective within both classical and modern frameworks of pattern formation. The analytic instability criterion $D_{n}\xi_{c}<\eta_{n}\chi_{\mathrm{eff}}n_{0}$ links tumor angiogenesis directly to Turing morphogenesis theory [23]. This connection has implications for tissue engineering and regenerative medicine, where predictable vascularization is required to sustain cell viability and ensure homogeneous delivery of oxygen and therapeutics. Our results highlight tunable parameter levers, such as TAF clearance, chemotactic sensitivity, and endothelial motility, that can be adjusted to promote or suppress vascular patterning.

Previous hybrid PDE-ABM studies emphasized coupling discrete and continuum representations [17] but lacked analytic instability criteria. Our analysis fills the gap by showing how bidirectional endothelial-TAF coupling alone suffices to create vascular patterning and protected niches that can foster drug resistance. This finding extends prior computational models with predictive criteria derived from stability theory. This approach also resonates with recent work on chemotaxis and pattern formation, where coupling reaction–diffusion dynamics and chemotaxis enlarges instability regions and drives morphogenetic patterning in development [67]. Here, we adapt that perspective to pathological angiogenesis, showing that endothelial autocrine production can convert vessels from passive responders to active drives of spatial self-organization.

The biological and translational implications arise directly from the instability condition and the numerical simulations. Within tumors, endothelial-driven hypoxic refugia create protected niches that favor resistant clones and limit drug penetration. Although these features are well-known in tumor microenvironments, prior work has not connected them to bidirectional endothelial-TAF coupling. Our simulations (Figure 11, Figure 12 and Figure 13) confirm the theoretical analysis by showing that spatial Turing patterns appear only under bidirectional coupling when reduced TAF diffusion coefficient and elevated chemotactic sensitivity drive the system above the instability threshold. Increasing the endothelial diffusion coefficient or removing bidirectional feedback suppresses pattern formation. The parameters $\eta_{n}$, $D_{c}$, $D_{n}$, $\xi_{c}$, and $\chi_{0}$ therefore act together to regulate vascular heterogeneity. These findings suggest that feedback-induced endothelial patterning can create hypoxic refugia and that targeted modulation of chemotaxis, TAF clearance, or vascular motility may reduce such resistance niches, informing therapeutic design and vascular engineering strategies.

Although linear stability analysis predicts the onset and dominant wavelength of vascular patterns, nonlinear effects determine long-term morphology and amplitude. Branching, anastomosis, and other discrete ABM dynamics are likely to influence pattern selection. A systematic bifurcation analysis, supported by ensemble simulations across mutation rates, repair dynamics, and therapy schedules, would clarify how nonlinear interactions shape perfusion heterogeneity and resistance emergence. Such extensions are necessary to translate mathematical predictions into robust therapeutic strategies.

Despite its contributions, the present study should be viewed as a mechanistic proof of principle. Several limitations warrant discussion:

(i) Dimensionality. The model is restricted to a two-dimensional tissue slice with homogeneous Neumann boundaries, whereas real tumors and engineered tissues are three-dimensional with irregular geometries and mixed boundary conditions. Spectral theory indicates that the number of Laplacian eigenvalues below a given threshold λ scales as $N\left(\lambda \right)\propto \mathrm{vol}\left(\Omega \right)\lambda^{d/2}$, so more unstable modes arise in 3D (d=3) than in 2D (d=2). Consequently, our 2D simulations provide a conservative estimate: real 3D tissues with tortuous vasculature are expected to exhibit broader unstable bands, richer spatial patterning, and potentially lower thresholds for instability. Extending the framework to three dimensions will therefore be essential for quantitative calibration against in vivo data.
(ii) Biochemical kinetics and transport. Linear kinetic laws for oxygen and drugs capture low-concentration dynamics but neglect Michaelis–Menten saturation and active efflux. Blood flow is treated as idealized and constant, though in vivo perfusion is dynamic, rerouted, and occasionally reversed within single vessels [68]. Incorporating spatiotemporally variable flow fields and nonlinear kinetics would refine predictions of hypoxia, perfusion heterogeneity, and resistance niches.
(iii) Microenvironmental and therapeutic detail. Immune and stromal cells, which profoundly affect tumor progression and therapy response, are not explicitly represented. Drug dynamics are simplified as diffusion, uptake, and decay, without accounting for pharmacokinetics/pharmacodynamics or combination therapies. These omissions limit realism in resistance evolution and therapeutic predictions; coupling with immune ABMs and pharmacological models will be needed.

We discuss additional caveats, including mechanics, motility variants, alternative resistance schemes, and applications beyond oncology, in Appendix J.

We treat this study as a mechanistic proof-of-principle rather than a simulation atlas. We intentionally focus on deriving analytic instability thresholds and hybrid coupling principles, leaving full-scale PDE-ABM visualizations and biological validation for future work. To ensure reproducibility, we provide the scripts used to generate the analytic figures (dispersion plots, bifurcation plots, and Turing patterns; Figure 9, Figure 10, Figure 11, Figure 12 and Figure 13). Extending this methodological framework into a general-purpose computational platform capable of visualizing vessel morphologies and resistance niches will be an important next step.

Together, our framework links reaction–diffusion theory to vascular clustering and perfusion heterogeneity, which in turn foster drug-resistant niches. Bidirectional endothelial-TAF coupling constitutes a robust mechanism for chemotaxis-driven Turing instabilities in tumor angiogenesis. The analytic threshold and the instability condition $D_{n}\xi_{c}<\eta_{n}\chi_{\mathrm{eff}}n_{0}$ identify concrete intervention points targeting feedback disruption to suppress vascular patterning and resistance evolution. Addressing the dimensional, kinetic, and microenvironmental simplifications highlighted above will be essential to enhance the model’s predictive power and translational relevance, broadening its applicability to both oncology and regenerative medicine. More broadly, this study demonstrates how hybrid mathematical models can yield actionable biological insights, serving as a bridge between theoretical analysis, experimental validation, therapeutic design, and regenerative medicine.

## Figures and Tables

**Table 1:** Mechanisms encoded in each partial differential equation (PDE) for endothelial cells *n*, tumor angiogenic factor (TAF) *c*, drug *d*, and oxygen *o*. Diffusion coefficients are denoted by $D_{\phi }$, decay rates by $\xi_{\phi }$, uptake rates by λ or $\rho_{\phi }$, and source terms by η or $S_{\phi }$. Uptake terms are sinks (negative signs in PDEs). Decay terms also come with negative signs in PDEs.

Field	Diffusion	Decay	Uptake	Supply
*n*	$D_{n}$	None	None	None
*c*	$D_{c}$	$\xi_{c}$	λc	η from hypoxic cells
*d*	$D_{d}$	$\xi_{d}$	$\rho_{d}d$	$S_{d}\left(t\right)$ at vessels
*o*	$D_{o}$	$\xi_{o}$	$\rho_{o}$	$S_{o}\left(o_{\max}-o\right)$

**Table 2:** Characteristic quantities used for nondimensionalization.

Symbol	Quantity	Rationale
*L*	Length	Spatial extent of parent vessel to tumor distance
$\tau =L^{2}/D$	Time	Typical diffusion time scale or cell cycle duration
$n_{0}$, $c_{0}$, $d_{0}$, $o_{\max}$	Field concentrations	Normalization of PDE variables: $n_{0},c_{0},d_{0}$ are endothelial, TAF, drug scales, respectively, and the maximum concentration $o_{\max}$ is the oxygen scale

**Table 3:** The table lists all model parameters, providing their dimensional values (D-values, in SI units), corresponding nondimensional values (ND-values), and sources or justifications; see also the machine-readable files params.json and params.csv in the GitHub Repository in the Data Availability Statement Section. Nondimensionalization is performed with respect to the characteristic length (*L*), time scale ($\tau =L^{2}/D_{o}$), and concentration ($c_{0}$). Parameters labeled as “calibrated” were chosen to ensure consistency with established biological behavior, whereas those marked “(n/a)” correspond to quantities without currently available empirical measurements. Table 4 provides a complete description of all parameters and their modeling roles.

Parameter	Description	D-Value (SI Units)	ND-Value	Provenance
Δx	Spatial discretization	$2.5\times 10^{-5}$ m	0.005	Calculated
Δt	Temporal discretization	CFL condition (Equation (13))	CFL condition (Equation (13))	Stability constraint
*h*	Slab thickness for dimension conversion	$1\times 10^{-4}$ m	0.02	[30]
$R_{c}$	Tumor and vessel cell radius	$1.25\times 10^{-5}\;m$	0.005	[31]
$D_{c}$	TAF diffusion coefficient	$5.21\times 10^{-11}\;m^{2}/s$	0.12	[32,33]
$\xi_{c}$	TAF decay rate	$3.47\times 10^{-8}\;s^{-1}$	0.002	[34]
η	TAF production rate	$1.7\times 10^{-22}\;\mathrm{mol}/\left(\mathrm{cell}\cdot s\right)$	$6.27\times 10^{3}$	[35]
λ	TAF uptake rate	$2.71\times 10^{-20}\;m^{3}/\left(\mathrm{cell}\cdot s\right)$	0.1	[25]
$D_{d}$	Drug diffusion coefficient	$2.17\times 10^{-10}\;m^{2}$/s	0.5	[36]
$\xi_{d}$	Drug decay rate	$1.74\times 10^{-7}\;s^{-1}$	0.01	[18]
$\rho_{d}$	Drug uptake rate	$1.36\times 10^{-19}\;m^{3}/\left(\mathrm{cell}\cdot s\right)$	0.5	[18]
$S_{d}$	Drug supply rate	$3.94\times 10^{-20}\;\mathrm{mol}/\left(\mathrm{cell}\cdot s\right)$	2	[18]
$p_{r}$	Damage clearance rate	(n/a, nondimensionalized)	0.2	[18]
$D_{o}$	Oxygen diffusion coefficient	$2.78\times 10^{-10}\;m^{2}/s$	0.64	[37]
$\xi_{o}$	Oxygen decay rate	$4.34\times 10^{-7}\;s^{-1}$	0.025	[38]
$\rho_{o}$	Oxygen uptake rate	$6.25\times 10^{-17}\;\mathrm{mol}/\left(\mathrm{cell}\cdot s\right)$	34.39	[18]
$S_{o}$	Oxygen supply rate	$9.33\times 10^{-19}\;m^{3}/\left(\mathrm{cell}\cdot s\right)$	3.44	[39]
ε	Tumor motility intensity	(n/a)	0.0215	[40]
$o_{\max}$	Maximum oxygen concentration	$6.7\;\mathrm{mol}/m^{3}$	1	[35]
$o_{\mathrm{hyp}}$	Hypoxia threshold	$1.675\;\mathrm{mol}/m^{3}$	0.25	[35]
$o_{\mathrm{apop}}$	Apoptosis threshold	$0.335\;\mathrm{mol}/m^{3}$	0.05	[35]
$D_{n}$	Endothelial diffusion coefficient	$2.00\times 10^{-13}\;m^{2}/s$	$4.61\times 10^{-4}$	[25]
$\chi_{0}$	Chemotaxis coefficient	$2.60\times 10^{-4}\;m^{5}/\left(s\cdot \mathrm{mol}\right)$	0.0599	[25]
α or $k_{1}$ ($\alpha =c_{0}/k_{1}$)	Chemotaxis saturation parameter	$1.6667\times 10^{-7}\;\mathrm{mol}/m^{3}$	0.6	[25]
ψ	Minimum branching age	$6.48\times 10^{4}$ s	1.125	[25]
$c_{br}$	Baseline branching rate	(n/a)	1	[19]
$a_{S}^{death}$	Death threshold (sensitive cells)	(n/a)	0.5	[18]
$Th_{\mathrm{multi}}$	Death threshold ratio (resistant cells)	5	5	[18]
${℘}_{age}$	Cell cycle duration	$\mathrm{Uniform}[3.24\times 10^{4},3.96\times 10^{4}]\;s$	0.56–0.69	[41,42]
$\alpha_{n}$	Proliferation rate	Derived from $\log\left(2\right)/{℘}_{age}$	1.0082–1.2323	Derived
$F_{max}$	Maximum neighbor cell count	10	10	[19]

**Table 4:** Summary of all parameters used in the model, grouped by type: PDE system and agent-based model (ABM). This table provides an overview of the variables and their modeling roles. For specific numerical values (dimensional and nondimensional) and their units, refer to Table 3.

Parameter	Meaning
PDE-related parameters	
$D_{n},D_{c},D_{d},D_{o}$	Diffusion coefficients of endothelial cells (*n*), TAF (*c*), drug (*d*), and oxygen (*o*)
$\chi_{0}$	Chemotactic sensitivity coefficient
α	Saturation parameter for chemotaxis
$\xi_{c},\xi_{d},\xi_{o}$	Natural decay rates of TAF, drug, and oxygen, respectively
$\rho_{d},\rho_{o}$	Cellular uptake rates of drug and oxygen
$S_{d},S_{o}$	Vessel supply rates of drug and oxygen
η,λ	TAF production rate by hypoxic cells and uptake rate by endothelial cells
$\phi_{a},\phi_{v}$	Normalized indicator functions for tumor agents and vessel locations
$R_{c}$	Tumor and vessel cell radius
ABM-related parameters	
$\Lambda_{t}$, $\Lambda_{t}^{n}$, $\Lambda_{t}^{h}$, $V_{t}$, $T_{t}$	Sets of all tumor cells, normoxic tumor cells, hypoxic tumor cells, vessel cells, and endothelial tip cells at time *t*
$A_{t}$	Angiogenic network at time *t*
$id_{a}$, $id_{b}$	Lineage identifiers for tumor and endothelial tip cells
$a^{x}\left(t\right)$, $b^{x}\left(t\right)$, $v^{x}\left(t\right)$	Spatial coordinates of agents $a\in \Lambda_{t}$, $b\in T_{t}$, $v\in V_{t}$ at time *t*
$a^{o}\left(t\right)$, $a^{d}\left(t\right)$, $a^{dam}\left(t\right)$, $a^{death}\left(t\right)$, $a^{age}\left(t\right)$, $a^{mat}$	Local oxygen, drug level, accumulated DNA damage, death threshold, age, and maturation time for tumor cell $a\in V_{t}$
$b^{age}\left(t\right)$	Age of endothelial tip cell $b\in T_{t}$
µ	Mutation intensity for the Poisson process
$p_{r}$	DNA damage repair or clearance rate
ε	Tumor cell motility coefficient
$o_{\max}$	Maximum oxygen concentration
$o_{\mathrm{hyp}},o_{\mathrm{apop}}$	Hypoxia threshold and apoptosis threshold for oxygen concentration
$P_{0},P_{1},P_{2},P_{3},P_{4}$	Probabilities of endothelial cell remaining stationary or moving left, right, down, or up
ψ	Minimum age required for tip branching
$c_{br}$	Branching intensity coefficient
$a_{S}^{death},a_{R}^{death}$	Death thresholds for sensitive and resistant tumor cells
$Th_{\mathrm{multi}}$	Multiplicative factor defining resistance death threshold ($a_{R}^{death}=Th_{\mathrm{multi}}\cdot a_{S}^{death}$)
${℘}_{age}$	Tumor cell cycle duration
$\alpha_{n}$	Proliferation rate of normoxic tumor cells
$F_{max}$	Crowding threshold above which proliferation is suppressed

**Table 5:** Neighborhood structures and spatial rules used for different cellular processes.

Cell Type	Process	Spatial Rule/Neighborhood
Tumor cell	Migration	Continuous Brownian motion (not lattice-confined)
Tumor cell	Branching (daughter placement)	Continuous, off-lattice positioning
Tumor cell	Crowding effect	$F_{\max}$ check within $R_{c}$ neighborhood
Tip cell	Migration	Von Neumann neighborhood (4 sites)
Tip cell	Branching (new tip placement)	Moore neighborhood (8 sites)
Tip cell	Occupancy/anastomosis check	Von Neumann neighborhood (4 sites)

**Table 6:** Critical domain length $L_{\mathrm{crit}}$ for instability under Neumann boundary condition. We report values in dimensionless units, with physical length obtained by multiplying by the characteristic scale L=5mm. All parameter values are taken from the authoritative set in Table 3; see also the machine-readable files params.json and params.csv in the GitHub Repository in the Data Availability Statement Section.

$\eta_{n}$ (Production)	Computed $\chi_{\mathrm{eff}}$	${\bar{\lambda }}_{p,q}$ Upper Bound	Critical Domain Length $L_{\mathrm{crit}}=\frac{\pi }{\sqrt{{\bar{\lambda }}_{p,q}}}$ (Dimensionless Units, 1 Unit = 5 mm)
0.001	0.04608	0.81625	3.47727
0.01	0.01498	2.69031	1.91535
0.05	0.00374	3.36706	1.71208
0.1	0.00193	3.47621	1.68499
0.5	0.00040	3.56873	1.66300
1	0.00020	3.58065	1.66023

**Table 7:** First nine Neumann Laplacian eigenmodes on the unit square and instability membership for representative $\eta_{n}$ values used in the manuscript. A checkmark (✓) indicates the mode satisfies $\lambda_{p,q}<{\bar{\lambda }}_{p,q}$ and therefore lies inside the unstable band for that $\eta_{n}$.

Index	Mode (p,q)	$\lambda_{p,q}$	$\eta_{n}=0.001$	$\eta_{n}=0.01$	$\eta_{n}=0.05$	$\eta_{n}=0.1$	$\eta_{n}=0.5$	$\eta_{n}=1$
1	(0, 0)	0	–	–	–	–	–	–
2	(0, 1)	$\frac{\pi^{2}}{25}\approx 0.3948$	✓	✓	✓	✓	✓	✓
3	(1, 0)	$\frac{\pi^{2}}{25}\approx 0.3948$	✓	✓	✓	✓	✓	✓
4	(1, 1)	$\frac{2\pi^{2}}{25}\approx 0.7896$	✓	✓	✓	✓	✓	✓
5	(0, 2)	$\frac{4\pi^{2}}{25}\approx 1.5791$		✓	✓	✓	✓	✓
6	(2, 0)	$\frac{4\pi^{2}}{25}\approx 1.5791$		✓	✓	✓	✓	✓
7	(1, 2)	$\frac{5\pi^{2}}{25}\approx 1.9739$		✓	✓	✓	✓	✓
8	(2, 1)	$\frac{5\pi^{2}}{25}\approx 1.9739$		✓	✓	✓	✓	✓
9	(2, 2)	$\frac{8\pi^{2}}{25}\approx 3.1583$			✓	✓	✓	✓

**Figure A1:**
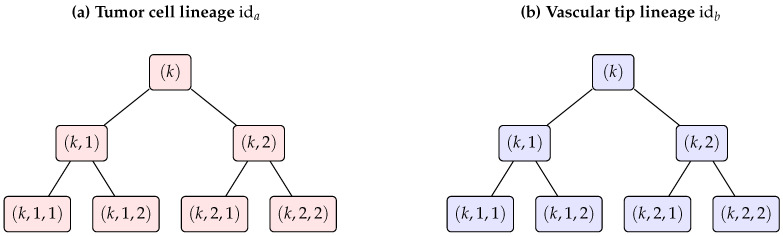
Binary trees showing hierarchical lineage identifiers for tumor cells ${\mathrm{id}}_{a}$ and vascular tips ${\mathrm{id}}_{b}$. Each node stores a tuple that records the ancestry (k), (k,1), (k,1,1), and so on. The layout presents tumor and vascular lineages side by side to emphasize their parallel branching structure while keeping notation consistent.

## Data Availability

The numerical simulation codes used to generate the (dispersion plots, bifurcation plots, and Turing patterns; Figure 9, Figure 10, Figure 11, Figure 12 and Figure 13) are publicly available at the GitHub repository (https://github.com/Louis-shuo-wang/BioEngineering.git) (accessed on 9 September 2025). The repository is released under the CC-BY 4.0 license to support reproducibility and transparency. We emphasize that only the simulation scripts are provided, as the present study focuses on methodological development rather than on implementing a complete hybrid simulation platform. We left the construction of such a general-purpose platform for future work.

## Abbreviations

    We use the following abbreviations in this manuscript:

ABC	ATP-binding cassette
ABM	Agent-based model
ADI	Alternating direction implicit
CFL	Courant–Friedrichs–Lewy
DTP	Drug-tolerant persister
HIF-1α	Hypoxia-inducible factor-1α
i.i.d.	Independent and identically distributed
PDE	Partial differential equation
TAF	Tumor angiogenic factor
VEGF	Vascular endothelial growth factor

## Appendix A. Comparison of Modeling Works

To situate our hybrid discrete–continuum model within the field, we compare it to previous representative mathematical models of cancer, focusing on tumor growth, angiogenesis, and drug resistance. Table A1 highlights three clear contrasts across prior models. Continuum reaction–diffusion studies capture bulk chemotactic and diffusive transport but omit single-cell stochasticity and clonal evolution [14,34]. Agent-based and hybrid frameworks incorporate discrete cellular behaviors and local microenvironments but often treat vasculature as static or neglect explicit resistance dynamics [18,40]. Recent hybrid models add mechanical realism or multi-omics layers [19,69] yet they do not couple endothelial-sourced angiogenic feedback to evolving tumor clones or provide analytic criteria for pattern onset in heterogeneous tumor microenvironment. Our model closed these gaps by combining dynamic chemotactic angiogenesis, stochastic neutral mutation, and a bifurcation analysis that yields an explicit finite-domain threshold for Turing instability, thereby linking vascular self-organization to resistance emergence.

**Table A1:** Comparison of our hybrid discrete–continuum model with prior mathematical models of cancer. Features include model type, angiogenesis treatment, resistance modeling, spatial heterogeneity, and limitations relative to our work. The novelties of our model include coupling between dynamic angiogenesis and resistance evolution, neutral mutations, and spatial pattern formation.

Model/Reference	Type	Angio.	Resist.	Spatial	Limitations
Balding et al. (1985) [70]	Continuum (RD)	Dynamic (waves)	None	Low	Early RD for vessel tips; lacks resistance/tumor cells; our model adds hybrid resistance evolution.
Byrne et al. (1995) [14]	Continuum (RD)	Dynamic (chemotaxis)	None	Low	Chemotactic vessel growth; no mutations; our model integrates ABM for stochastic resistance.
Anderson et al. (1998) [25]	Hybrid (PDE-ABM)	Dynamic (branching)	None	Medium	2D hybrid angiogenesis; static tumor; our model extends to dynamic tumor cells, mutations.
Murray (2002) [71]	Continuum (Keller–Segel)	Dynamic (chemotaxis)	None	Low	Biased cell movement; no resistance; our model couples with ABM clonal dynamics.
Anderson (2005, 2007) [38,40]	Hybrid (PDE-ABM)	None	Spontaneous	High	Foundational hybrid invasion/resistance; our model adds preexisting mutations, spatial pattern formation.
Billy et al. (2009) [34]	Continuum (RD)	Dynamic	Preexisting/acquired	Medium	Continuum angiogenesis; limited spatial resistance; our model includes neutral mutations, hybrids.
Gevertz (2014) [18]	ABM	Static	Preexisting/acquired	High	Resistance niches; no dynamic vessels; our model adds dynamic angiogenesis.
Spill et al. (2015) [49]	Hybrid (mesoscopic)	Dynamic	None	High	Mesoscopic angiogenesis; no resistance; our model adds mutations, spatial pattern formation.
Sun (2018) [72]	Review	None	Various	Various	Lacks angio–resistance coupling.
Altrock et al. (2015) [12]	Review	Varies	Varies	Varies	General oncology review; our model specifies hybrid angiogenesis and resistance evolution.
Yin (2019) [13]	Review	Varies	Preexisting/acquired	Varies	Resistance review; our model contributes novel hybrid integration.
Flandoli (2023) [19]	Hybrid (PDE-ABM)	Dynamic (mechanics)	None	High	Lennard-Jones forces; no resistance; our model adds mutations, spatial pattern formation.
Jamali (2024) [73]	Review	None	General	High	Immune-tumor ABM; no angiogenesis; our model focuses on vascular dynamics and resistance.
Our Model (2025)	Hybrid (PDE-ABM)	Dynamic (chemotaxis, branching)	Preexisting/spontaneous	High	2D simplification

## Appendix B. Drug Efflux Extension

Active drug efflux mediated by ATP-binding cassette (ABC) transporters such as P-glycoprotein constitutes a well-documented resistance mechanism [47]. We extend the drug field to include efflux by adding a source term that represents active transport from intracellular compartments back into the extracellular space:

(A1) $$\begin{array}{cc} \partial_{t}d\left(x,t\right) & =D_{d}\Delta d-\xi_{d}d-\rho_{d}d\sum_{a}\phi_{a}\left(x\right)+\kappa_{d}\sum_{a}\phi_{a}\left(x\right). \end{array}$$

Here, $\kappa_{d}$ denotes the efflux rate and $\phi_{a}\left(x\right)$ is the spatial indicator of cell *a*. The efflux term replenishes the extracellular drug concentration d(x,t) locally and can prevent intracellular accumulation even when the extracellular field remains high, thereby conferring resistance even in well-perfused regions.

We can quantify the relative impact of efflux by comparing the effective uptake $\rho_{d}d$ with the efflux $\kappa_{d}$. If $\kappa_{d}$ dominates, the cumulative intracellular drug concentration $a^{d}$ may remain below the death threshold $a^{death}$ despite significant extracellular levels. Future studies could calibrate $\kappa_{d}$ against experimental measurements of P-glycoprotein activity and test whether efflux alters the predicted spatial niches of resistance.

## Appendix C. Agent Trait Vector

In our hybrid PDE–ABM framework, each agent is represented by a structured state vector that encodes spatial, temporal, and lineage attributes. We describe tumor cell agents first, followed by endothelial tip cell agents. Presenting both in parallel highlights their shared structure, including hierarchical identifiers and state variables, as well as their biological differences, such as biochemical sensing versus vascular branching.

### Appendix C.1. Tumor Cell Traits

We characterize each tumor cell $a\in \Lambda_{t}$ by the state vector

$$\begin{array}{c} a=\left\{{\mathrm{id}}_{a},\;a^{x}\left(t\right),\;a^{o}\left(t\right),\;a^{d}\left(t\right),\;a^{dam}\left(t\right),\;a^{death}\left(t\right),\;a^{age}\left(t\right),\;a^{mat}\left(t\right)\right\}. \end{array}$$

The variable $a^{x}\left(t\right)=\left(a^{X}\left(t\right),a^{Y}\left(t\right)\right)$ (unit: m) denotes cell center position. The variable $a^{o}\left(t\right)$ (unit: $\mathrm{mol}/m^{3}$) represents local oxygen concentration, and $a^{d}\left(t\right)$ (unit: $\left(\mathrm{mol}\cdot s\right)/m^{3}$) is the cumulative drug level. The variable $a^{dam}\left(t\right)$ (unit: $\left(\mathrm{mol}\cdot s\right)/m^{3}$) represents drug-induced DNA damage and increases with drug exposure. The death threshold $a^{death}\left(t\right)$ (unit: $\left(\mathrm{mol}\cdot s\right)/m^{3}$) specifies the lethal level of $a^{dam}$ that triggers cell death. Two additional variables $a^{age}\left(t\right)$ (unit: s) and $a^{mat}\left(t\right)$ (unit: s) track the time elapsed since the last division and the cell cycle duration, respectively.

At the agent level, a tumor cell dies when $a^{dam}\left(t\right)>a^{death}\left(t\right)$. We initialize $a^{dam}=0$ and allow it to increase monotonically with drug exposure. We initialize the death threshold $a^{death}$ from a baseline value $T_{k}$. For sensitive cells, $a_{S}^{death}=0.5$, whereas for resistant cells, $a_{R}^{death}=Th_{\mathrm{multi}}\cdot a_{S}^{death}$. In the preexisting resistance case, $Th_{\mathrm{multi}}=5$ so that $a_{R}^{death}=2.5$. In the spontaneous mutation case, $Th_{\mathrm{multi}}$ evolves neutrally within phenotype bounds. Thus, $a^{death}$ is assigned deterministically from resistance rules rather than sampled probabilistically.

For the PDE–ABM formulation, we nondimensionalize the state variables by scaling position with the characteristic length *L*, concentrations with reference values $o_{\max},d_{0}$, and times with the characteristic time $\tau =L^{2}/D$:

$$\begin{array}{cc} \; & {\tilde{a}}^{x}=\frac{a^{x}}{L},\;{\tilde{a}}^{o}=\frac{a^{o}}{o_{\max}},\;{\tilde{a}}^{d}=\frac{a^{d}}{d_{0}\cdot \tau },\;{\tilde{a}}^{dam}=\frac{a^{dam}}{d_{0}\cdot \tau },\\ \; & {\tilde{a}}^{death}=\frac{a^{death}}{d_{0}\cdot \tau },\;{\tilde{a}}^{age}=\frac{a^{age}}{\tau },\;{\tilde{a}}^{mat}=\frac{a^{mat}}{\tau }. \end{array}$$

Henceforth, we omit tildes for readability and work exclusively with nondimensional variables.

We assign each tumor cell $a\in \Lambda_{t}$ a unique hierarchical lineage identifier:

$$\begin{array}{c} {\mathrm{id}}_{a}=\left(k,i_{1},\ldots ,i_{n}\right). \end{array}$$

Here, $k\in \{1,\ldots ,N_{0}\}$ denotes the founding ancestor index. $i_{j}\in \left\{1,2\right\}$ records the branching choice at the *j*-th division, and n∈N is the number of mitotic generations since initiation. The founding population n=0 is labeled by ${\mathrm{id}}_{a}=\left(k\right)$. If a cell of generation *n* has identifier ${\mathrm{id}}_{a}=\left(k,i_{1},\ldots ,i_{n}\right)$, then mitosis yields two generation-(n+1) daughter cells with identifiers:

$$\begin{array}{c} {\mathrm{id}}_{a^{\prime }}=\left(k,i_{1},\ldots ,i_{n},1\right),\;{\mathrm{id}}_{a"}=\left(k,i_{1},\ldots ,i_{n},2\right). \end{array}$$

This scheme establishes a binary tree structure that uniquely encodes ancestry and permits precise reconstruction of tumor cell lineages. Figure A1 (left) illustrates the hierarchical tumor cell identifier scheme ${\mathrm{id}}_{a}$. Each identifier encodes the ancestor index *k* and the sequence of binary division decisions $\left(k,i_{1},\ldots ,i_{n}\right)$. The root node (k) corresponds to the founding ancestor. Each mitotic event produces two daughter nodes, labeled by appending either “1” or “2” to the parent’s identifier. This recursive process yields a binary tree where each branch uniquely represents a mitotic lineage, allowing for the unambiguous reconstruction of any cell’s ancestry.

### Appendix C.2. Tip Cell Traits

In parallel to tumor agents, each endothelial tip cell $b\in T_{t}$ is characterized by

$$\begin{array}{c} b=\{{\mathrm{id}}_{b},b^{x}\left(t\right),\;b^{age}\left(t\right)\}. \end{array}$$

Here, $b^{x}\left(t\right)$ (unit: m) denotes position and $b^{age}$ (unit: s) tracks the time since the last branching. The unique identifier ${\mathrm{id}}_{b}$ encodes the hierarchical lineage of vascular tips, ensuring that each newly generated tip inherits its parent’s identifier with an appended branch label. In this way, ${\mathrm{id}}_{b}$ systematically records both the original tip and all subsequent branching events, following the same lineage-tracking rules used for tumor cell identifiers ${\mathrm{id}}_{a}$. Figure A1 (right) shows the analogous scheme for vascular tip identifiers ${\mathrm{id}}_{b}$. Similar to tumor cells, each identifier encodes the ancestor index *k* and the sequence of binary branching choices $\left(k,j_{1},\ldots ,j_{m}\right)$. The initial tip of the vessel is labeled (k). Each branching event produces two daughter tips, labeled by appending ‘1’ or ‘2’ to the parent’s label. This approach creates a binary tree structure that systematically captures the hierarchy of branching events. It also enables a precise reconstruction of the vascular lineage over successive branching generations. This lineage structure mirrors that of tumor cells, enabling analogous reconstruction of vascular sprouting hierarchies (Figure A1, right).

Together, the tumor and tip cell definitions provide a consistent bookkeeping system for heterogeneous cellular populations. Both employ hierarchical identifiers for ancestry, position vectors for spatial embedding, and time variables for dynamic updates. These parallel state-vector representations ensure compatibility between the PDE fields (oxygen, drug, TAF) and the ABM components (tumor proliferation, vascular branching), which is essential for the integrated hybrid framework.

## Appendix D. ABM Update Algorithm

For reproducibility and transparency, we detail the hybrid PDE-ABM update operator, which couples PDE fields (oxygen, drug, TAF) with discrete agents (tumor cells, tip cells, and vessels) in a two-way scheme (Figure 4).

The PDE solver advances concentrations using the ADI scheme (Equation (19)). Source terms derive from agent states at the previous ABM step. After solving the PDEs, we interpolate updated fields at agent positions to guide the ABM update. Endothelial density appears only through discrete vessel and tip agents and does not enter the PDE formulation.

The ABM executes every $\Delta t^{\prime }=m\Delta t$ to update tumor and tip cell populations and the angiogenic network. Algorithm A2 specifies the discrete agent rules: tumor cells undergo mutation, migration, death, aging, and proliferation based on local PDE values and intrinsic traits; tip cells undergo chemotactic migration, anastomosis, branching, and proliferation; vessel segments extend to reflect network growth. The ABM outputs $\left(\Lambda_{t_{k+1}},T_{t_{k+1}},V_{t_{k+1}}\right)$ and $A_{t_{k+1}}$, which refresh PDE sources for the next iteration.

Together, the flowchart and algorithms fully describe the coupled PDE-ABM time-stepping procedure, ensuring reproducibility while clearly separating PDE evolution, ABM dynamics, and coupling logic.

**Algorithm A1** Hybrid PDE–ABM solver (PDE update and coupling). The ABM update is summarized here for completeness, while Algorithm A2 provides detailed rules.
**Require:** Grid $\left(N_{x},N_{y},\Delta x,\Delta y\right)$, current time $t=t_{k}=k\Delta t^{\prime }$, current fields $c\left(\cdot ,t_{k}\right),d\left(\cdot ,t_{k}\right),o\left(\cdot ,t_{k}\right)$, agent sets (tumor cells $\Lambda_{t_{k}}$, tips $T_{t_{k}}$, vessels $V_{t_{k}}$), final time $T_{\max}$, PDE time step Δt, ABM step $\Delta t^{\prime }=m\Delta t$. **Ensure:** Updated fields and agent states until $t=T_{\max}$. 1: Initialize RNG(seed), set t←0. 2: Precompute geometry masks (tissue vs. vessel indicators). 3: **while** $t<T_{\max}$ **do** 4: **Load ABM sources:** Use agent states ($\Lambda_{t_{k}}$, $T_{t_{k}}$, $V_{t_{k}}$) from the previous ABM step to define PDE sources/sinks (oxygen uptake, VEGF secretion, drug metabolism). 5: **PDE update:** For p=1,…,m substeps of size Δt: (i) Apply ADI diffusion and explicit reaction updates to c,d,o with Neumann BCs (Equation (19)), using PDE fields from the previous PDE step $t=t_{k}+\left(p-1\right)\cdot \Delta t$ and ABM data from the previous ABM step $t=t_{k}$. (ii) Advance PDE clock: $t\leftarrow t_{k}+p\Delta t$. 6: **Sample PDE fields:** Interpolate updated $\left(c\left(\cdot ,t_{k+1}\right),d\left(\cdot ,t_{k+1}\right),o\left(\cdot ,t_{k+1}\right)\right)$ values at agent positions. 7: **ABM update:** Advance agents over one step $\Delta t^{\prime }$ (tumor proliferation, mutation, migration, death; tip anastomosis, branching, proliferation; angiogenic network update). See Algorithm A2. 8: **Coupling update:** From new agent states ($\Lambda_{t_{k+1}}$, $T_{t_{k+1}}$, $V_{t_{k+1}}$), refresh PDE sources/sinks for the next iteration. 9: **Output:** Save PDE fields and agent states if *t* is a checkpoint. 10: **end while**

## Appendix E. Unit Conversion in 2D and 3D and Parameter Estimation

Hybrid PDE–ABM models necessarily couple discrete cell densities with continuum concentrations. This requires explicit attention to unit consistency when performing two-dimensional simulations while drawing upon three-dimensional biological data.

**Remark** **A1** (Units and 2D vs. 3D consistency).
*We use normalized indicator functions $\phi_{a},\phi_{v}$ defined by*

$$\begin{array}{c} \phi_{a}\left(x,t\right)=\frac{1}{\pi R_{c}^{2}}\phi_{\left\{∥x-a^{x}\left(t\right)∥\leq R_{c}\right\}},\;\phi_{v}\left(x,t\right)=\frac{1}{\pi R_{c}^{2}}\phi_{\left\{∥x-v^{x}\left(t\right)∥\leq R_{c}\right\}}. \end{array}$$

*Then $\phi_{a},\phi_{v}$ have units $\mathrm{cell}/m^{2}$ and $\int_{U}\sum_{a}\phi_{a}\left(x,t\right)\;dx$ equals the number of cells. Hence, $\sum_{a}\phi_{a}\left(x,t\right)$ is the local areal cell density (cells per $m^{2}$).*

*In the three-dimensional formulation, these normalized indicator functions correspond to volumetric densities ($\mathrm{cell}/m^{3}$), so that $\sum_{a\in \Lambda_{t}}\phi_{a}$ and $\sum_{v\in V_{t}}\phi_{v}$ represent local volumetric cell densities. This interpretation is consistent with how the literature typically reports per cell secretion and uptake rates ($\eta ,\lambda ,\rho_{d},S_{d},\rho_{o},S_{o}$) in volumetric units. Accordingly, $n_{0}$ is introduced as the characteristic volumetric cell density and serves as the reference scale for these agent-based source and sink terms.*

*We can adopt two consistent modeling choices and state them explicitly:*

(i) *Volumetric (3D) concentrations. If $c=c_{3D}$ denotes a volumetric concentration (mol/$m^{3}$), the areal cell density can be converted to a volumetric cell density by dividing by an effective tissue thickness h (m). The source and uptake terms take the form*
  $$\begin{array}{c} \eta_{3D}\frac{\sum_{a}\phi_{a}}{h}\;\mathrm{and}\;\lambda_{3D}\;c_{3D}\frac{\sum_{v}\phi_{v}}{h}. \end{array}$$
  *Here, $\eta_{3D}$ has units mol/(cell·s) and $\lambda_{3D}$ has units $m^{3}$/(cell·s). These terms yield mol/($m^{3}$·s), as required for $\partial_{t}c_{3D}$.*
(ii) *Areal (2D) concentrations. If we instead evolve the slab-integrated concentration $c_{2D}=h\;c_{3D}$ ($\mathrm{mol}/m^{2}$), then the PDE uses areal source and uptake terms:*
  $$\begin{array}{c} \eta_{3D}\sum_{a}\phi_{a}\;\mathrm{and}\;\left(\frac{\lambda_{3D}}{h}\right)\;c_{2D}\sum_{v}\phi_{v}, \end{array}$$
  *so that $\eta_{2D}=\eta_{3D}$ (mol/(cell·s)) and $\lambda_{2D}=\lambda_{3D}/h$ ($m^{2}/\left(\mathrm{cell}\cdot s\right)$). Under the thin-slab assumption (no z variation), the in-plane diffusion coefficient $D_{c}$ and decay rate $\xi_{c}$ remain numerically unchanged.*

*Similar dimensional transitions apply to the drug and oxygen Equations (* 3 *)–(* 4 *). In the main text and parameter table (Table 3), we therefore report dimensional parameter values in standard 3D units (mol/$m^{3}$, cell/$m^{3}$, etc.), as commonly found in the literature. For two-dimensional simulations, the explicit thickness $h=1\times 10^{-4}$ m [30] is used to perform the required conversions. This ensures dimensional consistency between the discrete cell representations $\phi_{a},\phi_{v}$ and the continuous fields c,d,o. Nondimensionalization is then carried out using three-dimensional units, consistent with literature values and readily extendable to future 3D implementations without rescaling.*

Having established how volumetric and areal formulations are related, we next justify the specific parameter values adopted in our study. This ensures that subsequent nondimensionalization and simulation results can be traced back to biologically meaningful quantities.

We justify our modeling choice of each parameter.

(i) For the slab thickness used in areal and volumetric parameter dimension conversions, we set $h=1\times 10^{-4}$ m. This choice of *h* corresponds to a typical thin tissue slice used in in vitro assays and clinical research and ensures adequate oxygen diffusion [30]. This explicit choice ensures that all volumetric-to-areal and areal-to-volumetric conversions are fully traceable and reproducible.

(ii) The dimensional value for λ is $\lambda =2.71\times 10^{-20}\;m^{3}/\left(\mathrm{cell}\cdot s\right)$ from the nondimensionalization $\tilde{\lambda }=\lambda \tau n_{0}$.

(iii) The typical diffusion coefficient for chemotherapeutic drugs range from $10^{-13}$–$10^{-9}\;m^{2}$/s for chemotherapeutic drugs [36]. We chose the nondimensional value of $D_{d}=0.5$, corresponding to $D_{d}=2.17\times 10^{-10}\;m^{2}$/s, comparable to the value $3.4\times 10^{-10}\;m^{2}$/s for the anticancer drug doxorubicin.

(iv) The dimensional value for $\xi_{d}$ is $\xi_{d}=1.74\times 10^{-7}\;s^{-1}$ from the nondimensionalization ${\tilde{\xi }}_{d}=\tau \xi_{d}$.

(v) The dimensional value for $\rho_{d}$ is $\rho_{d}=1.36\times 10^{-19}\;m^{3}/\left(\mathrm{cell}\cdot s\right)$ from the nondimensionalization ${\tilde{\rho }}_{d}=\rho_{d}\tau_{0}$.

(vi) The dimensional value for $S_{d}$ is $S_{d}=3.94\times 10^{-20}\;\mathrm{mol}/\left(\mathrm{cell}\cdot s\right)$ from the nondimensionalization ${\tilde{S}}_{d}=\frac{S_{d}\tau n_{0}}{d_{0}}$ with $d_{0}=7.26\times 10^{-2}\;\mathrm{mol}/m^{3}$ [36].

(vii) Experimental oxygen supply rate spans the range $4\times 10^{-4}$–$1.39\;\mathrm{mol}/(m^{3}\cdot s)$ [39]. To calibrate our model, we require that the oxygen supply term in Equation (4), $S_{o}\;\left(o_{\max}-o\right)\sum_{v\in V_{t}}\phi_{v}$, matches the reported supply rate. Imposing this condition at baseline yields $S_{o}\;o_{\max}\;n_{0}\;=\;4\times 10^{-4}\;\mathrm{mol}/\left(m^{3}\cdot s\right)$, from which we obtain a dimensional estimate $S_{o}\;=\;9.33\times 10^{-19}\;m^{3}/\left(\mathrm{cell}\cdot s\right)$. Applying the nondimensionalization gives ${\tilde{S}}_{o}\;=\;S_{o}\;\tau \;n_{0}\;=\;3.44$.

(viii) Tumor necrotic core oxygen concentration measured ranges between 0.5–30% of the surrounding tissue level [35]. We chose $o_{\mathrm{hyp}}=0.25$ as the hypoxia threshold and $o_{\mathrm{apop}}=0.05$ as the apoptosis threshold.

(ix) Prior models lacked a consistent motility mechanism. Flandoli et al. [19] incorporated interactions via the Lennard–Jones potential and random movement, and the random motility coefficient $\varepsilon =10^{-3}$. We model random motility as Brownian diffusion. The corresponding Fokker–Planck equation for tumor probability density m(x,t) is

$$\begin{array}{c} \partial_{t}m=\frac{\varepsilon^{2}}{2}\Delta m, \end{array}$$

where effective diffusivity $\varepsilon^{2}/2$. We calibrate ε to match the experimental diffusivity $10^{-13}\;m^{2}/s$ [40], normalized by the reference diffusion $D=4.34\times 10^{-10}\;m^{2}/s$. Therefore, ε=0.0215 (dimensionless).

(x) $Th_{\mathrm{multi}}$ derives from IC50 values for non-small-cell lung cancer PC9 cells treated with tyrosine kinase inhibitors. Reported values of 100--1000 exceed the biologically reasonable range for our model. Gevertz et al. [18] used $Th_{\mathrm{multi}}=5$, which we also adopt.

Taken together, the unit conversions and parameter estimations provide a transparent foundation for our nondimensionalization procedure. They also ensure that the proposed two-dimensional simulations in this framework remain quantitatively anchored in experimentally reported three-dimensional data, facilitating reproducibility and future model extensions. The conversion also allows translating two-dimensional simulation outputs to three-dimensional biological and clinical predictions.

## Appendix F. Linear Stability Calculation

We collect here the full derivations of the dispersion relations used in Section 4. For readability, the main text only states the final instability criteria with concise proof sketches; the intermediate algebraic steps are given here for completeness and reproducibility.

### Appendix F.1. Proof of Theorem 2 (Unidirectional Coupling: Pattern Suppression)

**Proof.** Assuming a nonzero spatially homogeneous steady state $\left(m_{0},o_{0},n_{0},c_{0}\right)$, then it is easy to see $\left(m_{0},o_{0},n_{0},c_{0}\right)$ satisfies the following:

$$\begin{array}{c} \left\{\begin{array}{c} \alpha_{n}m_{0}\left(1-m_{0}/m_{\max}\right)H\left(o_{0}-o_{\mathrm{hyp}}\right)=0,\\ \xi_{o}o_{0}+\rho_{o}m_{0}=S_{o}\left(1-o_{0}\right)n_{0},\\ \eta m_{0}H\left(o_{\mathrm{hyp}}-o_{0}\right)=\xi_{c}c_{0}+\lambda c_{0}n_{0}. \end{array}\right. \end{array}$$

From the third equation, if $c_{0}=0$, then $o_{0}>o_{\mathrm{hyp}}$, while $o_{0}<o_{\mathrm{hyp}}$ when $c_{0}>0$. In the normoxic steady state $c_{0}=0$ and $o_{0}>o_{\mathrm{hyp}}$, and the steady state is $\left(m_{0},o_{0},n_{0},c_{0}\right)=\left(m_{\max},o_{0},n_{0},0\right)$. Since $c_{0}=0$, $\chi \left(c_{0}\right)=\chi_{0}$. The small perturbation $\left(\tilde{m},\tilde{o},\tilde{n},\tilde{c}\right)$ satisfies the following:

$$\begin{array}{c} \left\{\begin{array}{c} \partial_{t}\tilde{m}=D_{m}\Delta \tilde{m}-\alpha_{n}\tilde{m},\\ \partial_{t}\tilde{o}=D_{o}\Delta \tilde{o}-\xi_{o}\tilde{o}-\rho_{o}\tilde{m}+S_{o}\left(1-o_{0}\right)\tilde{n}-S_{o}n_{0}\tilde{o},\\ \partial_{t}\tilde{n}=D_{n}\Delta \tilde{n}-\chi_{0}n_{0}\Delta \tilde{c},\\ \partial_{t}\tilde{c}=D_{c}\Delta \tilde{c}-\xi_{c}\tilde{c}-\lambda n_{0}\tilde{c}. \end{array}\right. \end{array}$$

We expand with respect to each spectral pair of the Neumann Laplacian:

$$\begin{array}{c} \Delta \phi_{j}+\lambda_{j}\phi_{j}=0\;\mathrm{on}\;U,\;\nabla_{\vec{n}}\phi_{j}=0\;\mathrm{on}\;\partial U \end{array}$$

where $\vec{n}$ is the outward normal vector for ∂U. Writing $\left(\tilde{m},\tilde{o},\tilde{n},\tilde{c}\right)=e^{\sigma t}\phi_{j}\left(\hat{m},\hat{o},\hat{n},\hat{c}\right)$ yields the following linear system:

$$\begin{array}{c} \left(\begin{array}{cccc} \sigma +D_{m}\lambda_{j}+\alpha_{n} & 0 & 0 & 0\\ \rho_{o} & \sigma +D_{o}\lambda_{j}+\xi_{o}+S_{o}n_{0} & -S_{o}\left(1-o_{0}\right) & 0\\ 0 & 0 & \sigma +D_{n}\lambda_{j} & -\chi_{0}n_{0}\lambda_{j}\\ 0 & 0 & 0 & \sigma +D_{c}\lambda_{j}+\xi_{c}+\lambda n_{0} \end{array}\right)\left(\begin{array}{c} \tilde{m}\\ \tilde{o}\\ \tilde{n}\\ \tilde{c} \end{array}\right)=\left(\begin{array}{c} 0\\ 0\\ 0\\ 0 \end{array}\right). \end{array}$$

The dispersion relation reads as follows:

$$\begin{array}{c} \left(\sigma +D_{m}\lambda_{j}+\alpha_{n}\right)\left(\sigma +D_{o}\lambda_{j}+\xi_{o}+S_{o}n_{0}\right)\left(\sigma +D_{n}\lambda_{j}\right)\left(\sigma +D_{c}\lambda_{j}+\xi_{c}+\lambda n_{0}\right)=0. \end{array}$$

We note that the first factor $\left(\sigma +D_{m}\lambda_{j}+\alpha_{n}\right)$ has solution $\sigma =-D_{m}\lambda_{j}-\alpha_{n}<0$. The three factors give eigenvalue $-(D_{o}\lambda_{j}+\xi_{o}+S_{o}n_{0})$, $-D_{n}\lambda_{j}$, and $-(D_{c}\lambda_{j}+\xi_{c}+\lambda n_{0})$, respectively. All eigenvalues are negative. Therefore, no spatial patterns emerge from this normoxic steady state. On the other hand, in the hypoxic state $c_{0}>0$ and $o_{0}<o_{\mathrm{hyp}}$, and the steady state is $\left(m_{0},o_{0},n_{0},c_{0}\right)$ with nonzero components. The small perturbation satisfies the following:

$$\begin{array}{c} \left\{\begin{array}{c} \partial_{t}\tilde{m}=D_{m}\Delta \tilde{m},\\ \partial_{t}\tilde{o}=D_{o}\Delta \tilde{o}-\xi_{o}\tilde{o}-\rho_{o}\tilde{m}+S_{o}\left(1-o_{0}\right)\tilde{n}-S_{o}n_{0}\tilde{o},\\ \partial_{t}\tilde{n}=D_{n}\Delta \tilde{n}-\chi \left(c_{0}\right)n_{0}\Delta \tilde{c},\\ \partial_{t}\tilde{c}=D_{c}\Delta \tilde{c}+\eta \tilde{m}-\xi_{c}\tilde{c}-\lambda c_{0}\tilde{n}-\lambda n_{0}\tilde{c}. \end{array}\right. \end{array}$$

The Fourier mode solution $\left(\tilde{m},\tilde{o},\tilde{n},\tilde{c}\right)=e^{\sigma t}\phi_{j}\left(\hat{m},\hat{o},\hat{n},\hat{c}\right)$ satisfies the following:

$$\begin{array}{c} \left(\begin{array}{cccc} \sigma +D_{m}\lambda_{j} & 0 & 0 & 0\\ \rho_{o} & \sigma +D_{o}\lambda_{j}+\xi_{o}+S_{o}n_{0} & -S_{o}\left(1-o_{0}\right) & 0\\ 0 & 0 & \sigma +D_{n}\lambda_{j} & -\chi \left(c_{0}\right)n_{0}\lambda_{j}\\ -\eta & 0 & \lambda c_{0} & \sigma +D_{c}\lambda_{j}+\xi_{c}+\lambda n_{0} \end{array}\right)\left(\begin{array}{c} \tilde{m}\\ \tilde{o}\\ \tilde{n}\\ \tilde{c} \end{array}\right)=\left(\begin{array}{c} 0\\ 0\\ 0\\ 0 \end{array}\right). \end{array}$$

The dispersion relation reads as follows:

$$\begin{array}{cc} \; & \left(\sigma +D_{m}\lambda_{j}\right)\left(\sigma +D_{o}\lambda_{j}+\xi_{o}+S_{o}n_{0}\right)\\ \; & \;\times \left[\left(\sigma +D_{n}\lambda_{j}\right)\left(\sigma +D_{c}\lambda_{j}+\xi_{c}+\lambda n_{0}\right)+\lambda c_{0}\chi \left(c_{0}\right)n_{0}\lambda_{j}\right]=0. \end{array}$$

We note that the first factor $\left(\sigma +D_{m}\lambda_{j}\right)$ has solution $\sigma =-D_{m}\lambda_{j}<0$. The second factor $\left(\sigma +D_{o}\lambda_{j}+\xi_{o}+S_{o}n_{0}\right)$ gives σ<0. The 2×2 *n*-*c* block’s characteristic polynomial is

$$\begin{array}{c} \sigma^{2}+\left(\left(D_{n}+D_{c}\right)\lambda_{j}+\xi_{c}+\lambda n_{0}\right)\sigma +D_{n}D_{c}\lambda_{j}^{2}+\left(D_{n}\xi_{c}+\lambda n_{0}D_{n}+\lambda c_{0}\chi \left(c_{0}\right)n_{0}\right)\lambda_{j}. \end{array}$$

All coefficients are positive, and the Routh–Hurwitz criterion implies roots do not have positive real parts. Therefore, no spatial patterns emerge from this hypoxic steady state. Therefore, both in the normoxic and hypoxic steady states, all nontrivial perturbations decay. These results demonstrate that, under the unidirectional endothelial-TAF coupling, chemotactic feedback alone is insufficient to generate Turing instabilities.    □

**Algorithm A2** Agent-based model (ABM) update rules, executed once per step $\Delta t^{\prime }=m\Delta t$ in the hybrid solver (Algorithm A1).
**Require:** Grid $\left(N_{x},N_{y},\Delta x,\Delta y\right)$, current time $t=t_{k}=k\Delta t^{\prime }$, current fields $c\left(\cdot ,t_{k}\right),d\left(\cdot ,t_{k}\right),o\left(\cdot ,t_{k}\right)$, agent sets (tumor cells $\Lambda_{t_{k}}$, tips $T_{t_{k}}$, vessels $V_{t_{k}}$), final time $T_{\max}$, PDE time step Δt, ABM step $\Delta t^{\prime }=m\Delta t$; tumor motility coefficient ε; DNA repair rate $p_{r}$; oxygen thresholds $o_{\mathrm{hyp}},o_{\mathrm{apop}}$; normoxic division rate $\alpha_{n}$; crowding threshold $F_{\max}$; resistance factor bounds [0.5,4]; mutation intensity µ; tip branching threshold ψ and coefficient $c_{br}$; chemotaxis parameters $\left(D_{n},\chi_{0},\alpha \right)$; vessel doubling time $\tau_{\mathrm{tip}}=1.125$. **Ensure:** Updated agent states at $t_{k+1}=t_{k}+\Delta t^{\prime }$. 1: **for** each agent *x* at $t_{k}$ **do** 2: **if** *x* is a **tumor cell** *a* **then** 3: **Mutation (neutral):** With probability $µ\Delta t^{\prime }$, update mutable traits ($a^{death}$, $\alpha_{n}$, oxygen consumption): (i) For each trait $x_{i}$, draw $r_{i}∼\mathrm{Uniform}\left[0.7,1.7\right]$, (ii) $x_{i,\mathrm{new}}=\min\left\{\max\left(r_{i}x_{i},0.5x_{i,\mathrm{baseline}}\right),4x_{i,\mathrm{baseline}}\right\}$. 4: **Motility:** Update $a^{x}$ by Brownian motion with coefficient ε (Euler–Maruyama). 5: **Sensing:** (i) $a^{o}\left(t_{k+1}\right)=o\left(a^{x}\left(t_{k}\right),t_{k}\right)$, (ii) $a^{d}\left(t_{k+1}\right)=a^{d}\left(t_{k}\right)+d\left(a^{x}\left(t_{k}\right),t_{k}\right)\Delta t^{\prime }$. 6: **Damage & death:** Update $a^{dam}$ via drug uptake/repair. Remove *a* if $a^{dam}>a^{death}$ or $a^{o}\leq o_{\mathrm{apop}}$. 7: **Phenotype:** Classify as normoxic ($o>o_{\mathrm{hyp}}$), hypoxic ($o_{\mathrm{apop}}<o\leq o_{\mathrm{hyp}}$), or apoptotic ($o\leq o_{\mathrm{apop}}$). 8: **Aging:** If normoxic, $a^{age}\leftarrow a^{age}+\Delta t^{\prime }$. 9: **Division:** If $a^{age}\geq a^{mat}=\log\left(2\right)/\alpha_{n}$ and local density $F\leq F_{\max}$: (i) Place daughter $a_{1}$ at *a*’s position; sample random displacement for $a_{2}$. (ii) Daughters inherit $a^{death},\alpha_{n}$, oxygen consumption; split $a^{dam},a^{d}$; reset ages to 0. 10: **else if** *x* is a **tip cell** *b* **then** 11: **Motility:** Select move among Von Neumann neighbors with probabilities $P_{0},\ldots ,P_{4}$ (Equation (17)). 12: **if** target neighbor occupied **then** 13: **Anastomosis:** Convert *b* into vessel, stop migration/branching. 14: **end if** 15: **Aging:** $b^{age}\leftarrow b^{age}+\Delta t^{\prime }$. 16: **Branching:** If $b^{age}>\psi$ and vacant Moore neighbor exists, branch with prob. $\lambda_{br}\left(b,t_{k}\right)\Delta t^{\prime }$: (i) Place new tip at vacant Moore site, (ii) Reset both tips’ ages to 0. 17: **Proliferation:** Every $\tau_{\mathrm{tip}}$, elongate sprout by adding vessel agent behind *b*. 18: **end if** 19: **end for** 20: **return** Updated agent sets $\left(\Lambda_{t_{k}+\Delta t^{\prime }},T_{t_{k}+\Delta t^{\prime }},V_{t_{k}+\Delta t^{\prime }}\right)$, updated angiogenic network $A_{t_{k}+\Delta t^{\prime }}$.

**Remark** **A2.**

(i) *Strictly speaking, in the normoxic and hypoxic scenario, the $\lambda_{j}=0$ Fourier mode should be interpreted separately. It corresponds to spatially uniform perturbations, for which σ=0 is a neutral stable eigenvalue. This trivial zero mode corresponds to conservation of total mass or the unchanged average state, and does not affect the pattern-forming dynamics. Our focus is on $\lambda_{j}>0$, where perturbations represent genuine spatial inhomogeneities. For these modes, no positive growth rates σ emerge, confirming that aside from the trivial zero mode, all perturbations decay.*
(ii) *Our linear stability analysis assumes that perturbations remain confined within a fixed oxygen regime, either normoxic or hypoxic. Specifically, in the hypoxic state where $o_{0}<o_{\mathrm{hyp}}$, a sufficiently small perturbation ensures that $\tilde{o}+o_{0}<o_{\mathrm{hyp}}$, so that the system does not transition into the normoxic regime. An analogous argument applies in the normoxic state. In contrast, if fluctuations were large enough to cross the hypoxia threshold, both regimes would need to be considered simultaneously. Such regime switching is not inherently problematic from a modeling standpoint. The difficulty arises from our use of a Heaviside function to represent VEGF secretion, since small fluctuations near the threshold would cause the function to oscillate rapidly between 0 and 1, introducing artificial discontinuities. Because VEGF secretion is known to vary smoothly with oxygen levels, a smooth functional form would be more appropriate in this case. A detailed analysis of this threshold-crossing scenario, however, lies beyond the scope of the present study.*

Having established that unidirectional coupling cannot produce spatial patterns, we now turn to the bidirectional case, which leads to the instability criterion summarized in Theorem 3.

### Appendix F.2. Proof of Theorem 3 (Bidirectional Coupling: Pattern Formation)

**Proof.** Denote the spatial perturbation by $\left(\tilde{n},\tilde{c}\right)=\left(n-n_{0},c-c_{0}\right)$. At the steady state $\left(n_{0},c_{0}\right)$, by plugging $\left(n_{0},c_{0}\right)$ into Equation (21), we have $\eta_{n}n_{0}-\xi_{c}c_{0}=0$. Then the perturbation satisfies the following:

$$\begin{array}{c} \left\{\begin{array}{c} \partial_{t}\tilde{n}=D_{n}\Delta \tilde{n}-\chi_{\mathrm{eff}}n_{0}\Delta \tilde{c},\\ \partial_{t}\tilde{c}=D_{c}\Delta \tilde{c}+\eta_{n}\tilde{n}-\xi_{c}\tilde{c}. \end{array}\right. \end{array}$$

Expressing the perturbation by Fourier modes $\left(\tilde{n},\tilde{c}\right)=e^{\sigma t}\phi_{j}\left(\hat{n},\hat{c}\right)$ yields the following:

$$\begin{array}{c} \left\{\begin{array}{c} \sigma \tilde{n}=-D_{n}\lambda_{j}\tilde{n}+\chi_{\mathrm{eff}}n_{0}\lambda_{j}\tilde{c},\\ \sigma \tilde{c}=-D_{c}\lambda_{j}\tilde{c}+\eta_{n}\tilde{n}-\xi_{c}\tilde{c}. \end{array}\right. \end{array}$$

A nonzero solution $\left(\tilde{n},\tilde{c}\right)\neq \left(0,0\right)$ implies the above linear system is singular, where we have the following:

$$\begin{array}{c} \mathrm{Det}\left(\begin{array}{cc} \sigma +D_{n}\lambda_{j} & -\chi_{\mathrm{eff}}n_{0}\lambda_{j}\\ -\eta_{n} & \sigma +D_{c}\lambda_{j}+\xi_{c} \end{array}\right)=0. \end{array}$$

Therefore, we obtain the dispersion relation $\sigma =\sigma (\lambda_{j})$ as follows:

$$\begin{array}{c} \sigma^{2}+a_{1}\left(k\right)\sigma +a_{0}\left(k\right)=0, \end{array}$$

where $a_{1}\left(k\right)=\left(D_{n}+D_{c}\right)\lambda_{j}+\xi_{c}$ and $a_{0}\left(k\right)=D_{n}D_{c}\lambda_{j}^{2}+\left(D_{n}\xi_{c}-\eta_{n}\chi_{\mathrm{eff}}n_{0}\right)\lambda_{j}$. Linear instability requires ℜ(σ)>0 for some wavenumber *k*. Since $a_{0}\left(k\right)>0$ for all wavenumbers *k*, the only possibility is when $a_{0}\left(k\right)<0$, which implies that

$$\begin{array}{c} D_{n}\xi_{c}-\eta_{n}\chi_{\mathrm{eff}}n_{0}<0, \end{array}$$

and the permissible range for *k* is

$$\begin{array}{c} {\underline{\lambda }}_{j}=0<\lambda_{j}<\frac{\eta_{n}\chi_{\mathrm{eff}}n_{0}-D_{n}\xi_{c}}{D_{n}D_{c}}{\bar{\lambda }}_{j} \end{array}$$

□

For transparency and reproducibility, we further provide below the full algebraic derivation of the quadratic dispersion relation $\sigma^{2}+a_{1}\left(\lambda \right)\sigma +a_{0}\left(\lambda \right)=0$ and its coefficients. Starting from the reduced nondimensional endothelial-TAF system (cf. Equation (21)), we study perturbations about a spatially homogeneous steady state $\left(n_{0},c_{0}\right)$. For a homogeneous steady state (no spatial variation), the steady-state equations reduce to

$$\begin{array}{cc} 0 & =-\xi_{c}c_{0}+\eta_{n}n_{0}\;⟹\;c_{0}=\frac{\eta_{n}n_{0}}{\xi_{c}}, \end{array}$$

while $n_{0}$ is the uniform base endothelial density (determined by initial/boundary conditions or slow demographic dynamics not included in the reduced model).

We next do the first-order expansion and obtain the linearized equations. Write

$$\begin{array}{c} n\left(x,t\right)=n_{0}+\varepsilon \tilde{n}\left(x,t\right),\;c\left(x,t\right)=c_{0}+\varepsilon \tilde{c}\left(x,t\right), \end{array}$$

and expand to O(ε). For the chemotaxis flux term, we use the Taylor expansion

$$\begin{array}{cc} \chi (c) & =\chi \left(c_{0}\right)+\chi^{\prime }\left(c_{0}\right)\;\left(c-c_{0}\right)+O\left({\left(c-c_{0}\right)}^{2}\right)\\ \; & =\chi \left(c_{0}\right)+\varepsilon \chi^{\prime }\left(c_{0}\right)\;\tilde{c}+\varepsilon^{2}O\left({\tilde{c}}^{2}\right) \end{array}$$

Note that $c_{0}$ is spatially uniform, and thus $\nabla c_{0}\equiv 0$ and $\nabla c=\varepsilon \nabla \tilde{c}$. Now

$$\begin{array}{cc} \chi (c)\;n\nabla c & =\left[\chi \left(c_{0}\right)+\varepsilon \chi^{\prime }\left(c_{0}\right)\;\tilde{c}+\varepsilon^{2}O\left({\tilde{c}}^{2}\right)\right]\left(n_{0}+\varepsilon \tilde{n}\right)\varepsilon \nabla \tilde{c}\\ \; & =\varepsilon \chi \left(c_{0}\right)n_{0}\nabla \tilde{c}+O\left(\varepsilon^{2}\right), \end{array}$$

Hence, to first-order, the chemotactic flux linearizes simply to $\chi \left(c_{0}\right)\;n_{0}\nabla \tilde{c}$. (We therefore write $\chi_{\mathrm{eff}}:=\chi \left(c_{0}\right)=\chi_{0}/\left(1+\alpha c_{0}\right)$.) The linearized system is

(A2) $$\begin{array}{c} \left\{\begin{array}{c} \partial_{t}\tilde{n}=D_{n}\Delta \tilde{n}-\nabla \cdot \left(\chi_{\mathrm{eff}}\;n_{0}\nabla \tilde{c}\right),\\ \partial_{t}\tilde{c}=D_{c}\Delta \tilde{c}-\xi_{c}\tilde{c}+\eta_{n}\tilde{n}. \end{array}\right. \end{array}$$

We continue to project perturbations on Laplacian eigenmodes and obtain the ODE system. Let ${\left\{\phi_{j}\right\}}_{j\geq 0}$ be an orthonormal basis of Laplacian eigenfunctions on *U* satisfying

$$\begin{array}{c} \Delta \phi_{j}=-\lambda_{j}\phi_{j}, \end{array}$$

with the appropriate boundary conditions (Neumann, periodic, etc.). Expand $\tilde{n}$ and $\tilde{c}$ in this basis:

$$\begin{array}{c} \tilde{n}\left(x,t\right)=\sum_{j}a_{j}\left(t\right)\phi_{j}\left(x\right),\;\tilde{c}\left(x,t\right)=\sum_{j}b_{j}\left(t\right)\phi_{j}\left(x\right). \end{array}$$

Substituting into Equation (A2) and taking the $L^{2}\left(U\right)$ inner product with each eigenfunction ϕ, each mode $\left(a_{j},b_{j}\right)$ satisfies the linear ODE system

(A3) $$\begin{array}{c} \frac{d}{dt}\left(\begin{array}{c} a_{j}\\ b_{j} \end{array}\right)=\left(\begin{array}{cc} -D_{n}\lambda_{j} & \;\chi_{\mathrm{eff}}\;n_{0}\;\lambda_{j}\\ \eta_{n} & -D_{c}\lambda_{j}-\xi_{c} \end{array}\right)\left(\begin{array}{c} a_{j}\\ b_{j} \end{array}\right). \end{array}$$

We next derive the dispersion relation. Let σ denote a growth rate (temporal eigenvalue) for a fixed spatial eigenmode $\lambda =\lambda_{j}$. Then $\frac{da_{j}}{dt}=\sigma a_{j}$, $\frac{db_{j}}{dt}=\sigma b_{j}$. The characteristic equation is

$$\begin{array}{c} \det\left(\begin{array}{cc} \sigma +D_{n}\lambda & -\chi_{\mathrm{eff}}\;n_{0}\;\lambda \\ -\eta_{n} & \sigma +D_{c}\lambda +\xi_{c} \end{array}\right)=0. \end{array}$$

Expanding yields the quadratic dispersion relation

(A4) $$\begin{array}{c} \sigma^{2}+a_{1}\left(\lambda \right)\;\sigma +a_{0}\left(\lambda \right)=0, \end{array}$$

with

(A5) $$\begin{array}{cc} a_{1}\left(\lambda \right) & =\left(D_{n}+D_{c}\right)\;\lambda +\xi_{c}, \end{array}$$

(A6) $$\begin{array}{cc} a_{0}\left(\lambda \right) & =D_{n}D_{c}\;\lambda^{2}+\left(D_{n}\xi_{c}-\eta_{n}\chi_{\mathrm{eff}}n_{0}\right)\;\lambda . \end{array}$$

These expressions are the forms used in Section 4.

We next derive the instability condition and critical wavenumber. Turing instability occurs if there exists λ>0 with $a_{0}\left(\lambda \right)<0$ and $a_{1}\left(\lambda \right)>0$ (so one root σ becomes positive while the other remains negative). From (A6), the unstable band (if any) is

$$\begin{array}{c} 0<\lambda <\bar{\lambda },\;\bar{\lambda }=\frac{\eta_{n}\chi_{\mathrm{eff}}\;n_{0}-D_{n}\xi_{c}}{D_{n}D_{c}}, \end{array}$$

so instability requires $\eta_{n}\chi_{\mathrm{eff}}n_{0}>D_{n}\xi_{c}$. Therefore, we can derive the critical domain size expressions quoted in the main text once the admissible discrete $\lambda_{j}$ (which depend on the boundary conditions) are substituted. For example, with Neumann boundary conditions on $U=\left[0,L_{a}\right]\times \left[0,L_{b}\right]$ the smallest nonzero λ is $\lambda_{\min}=\pi^{2}/\max{\left\{L_{a},L_{b}\right\}}^{2}$; with periodic boundary conditions it is $\lambda_{\min}={\left(2\pi \right)}^{2}/\max{\left\{L_{a},L_{b}\right\}}^{2}$, explaining the factor-of-two difference in the critical linear dimension.

We give some comment on $\chi_{\mathrm{eff}}$ vs. $\chi^{\prime }\left(c_{0}\right)$. We used $\chi_{\mathrm{eff}}=\chi \left(c_{0}\right)$ in the dispersion relation. The above first-order expansion shows why $\chi^{\prime }\left(c_{0}\right)$ does not appear at linear order. In the expansion of χ(c)n∇c, terms involving $\chi^{\prime }\left(c_{0}\right)$ is at least second order in ε:

$$\begin{array}{c} \varepsilon^{2}\chi^{\prime }\left(c_{0}\right)\tilde{c}n_{0}\nabla \tilde{c}\;\varepsilon^{3}\chi^{\prime }\left(c\right)\tilde{c}\tilde{n}\nabla \tilde{c}. \end{array}$$

They vanish if we consider the expansion to the leading order.

The steps above provide the full algebraic derivation of the dispersion polynomial $\sigma^{2}+a_{1}\left(\lambda \right)\sigma +a_{0}\left(\lambda \right)=0$ used in Section 4, and show explicitly how $\eta_{n},\chi_{\mathrm{eff}},D_{n},D_{c},\xi_{c}$ enter the instability criterion. This appendix provides a fully transparent and reproducible account of the linear stability calculation.

## Appendix G. Linear Stability for Michaelis–Menten System

To assess whether the linear feedback saturation $S_{o}\left(o_{\max}-o\right)\sum_{v}\phi_{v}$ materially affects the linear stability conclusions, we tested the effect of a Michaelis–Menten form per vessel,

$$\begin{array}{c} F(o:)=S_{o}\;\frac{u}{K_{s}+u}\sum_{v}\phi_{v},\;u:=o_{\max}-o, \end{array}$$

We first modify the undirectional system equation as

(A7) $$\begin{array}{c} \left\{\begin{array}{c} \partial_{t}m=D_{m}\Delta m+\alpha_{n}m\left(1-m/m_{\max}\right)H\left(o-o_{\mathrm{hyp}}\right),\\ \partial_{t}o=D_{o}\Delta o-\xi_{o}o-\rho_{o}m+F\left(o\right)n,\\ \partial_{t}n=D_{n}\Delta n-\nabla \cdot \left(\chi \left(c\right)n\nabla c\right),\\ \partial_{t}c=D_{c}\Delta c+\eta mH\left(o_{\mathrm{hyp}}-o\right)-\xi_{c}c-\lambda cn. \end{array}\right. \end{array}$$

Assume a nonzero spatially homogeneous steady state $\left(m_{0},o_{0},n_{0},c_{0}\right)$, then it satisfies

$$\begin{array}{c} \left\{\begin{array}{c} \alpha_{n}m_{0}\left(1-m_{0}/m_{\max}\right)H\left(o_{0}-o_{\mathrm{hyp}}\right)=0,\\ \xi_{o}o_{0}+\rho_{o}m_{0}=F\left(o_{0}\right)n_{0},\\ \eta m_{0}H\left(o_{\mathrm{hyp}}-o_{0}\right)=\xi_{c}c_{0}+\lambda c_{0}n_{0}. \end{array}\right. \end{array}$$

In the hypoxic case, $\left(m_{0},o_{0},n_{0},c_{0}\right)$ has nonzero components; in the normaxic case, $\left(m_{0},o_{0},n_{0},c_{0}\right)=\left(m_{\max},o_{0},n_{0},0\right)$. We perform linear stability analysis around $\left(m_{0},o_{0},n_{0},c_{0}\right)$.

In the normoxic case, expanding the small perturbation $\left(\tilde{m},\tilde{o},\tilde{n},\tilde{c}\right)$ with respect to the Neumann spectral pair $\left(\lambda_{j},\phi_{j}\right)$ yields the following linear system

$$\begin{array}{c} \left(\begin{array}{cccc} \sigma +D_{m}\lambda_{j}+\alpha_{n} & 0 & 0 & 0\\ \rho_{o} & \sigma +D_{o}\lambda_{j}+\xi_{o}-F^{\prime }\left(o_{0}\right)n_{0} & -F(o_{0}) & 0\\ 0 & 0 & \sigma +D_{n}\lambda_{j} & -\chi_{0}n_{0}\lambda_{j}\\ 0 & 0 & 0 & \sigma +D_{c}\lambda_{j}+\xi_{c}+\lambda n_{0} \end{array}\right)\left(\begin{array}{c} \tilde{m}\\ \tilde{o}\\ \tilde{n}\\ \tilde{c} \end{array}\right)=\left(\begin{array}{c} 0\\ 0\\ 0\\ 0 \end{array}\right). \end{array}$$

Since *F* is decreasing in oxygen concentration: $F^{\prime }\left(o\right)<0$ for o≥0, it is easy to see the dispersion relation

$$\begin{array}{c} \left(\sigma +D_{m}\lambda_{j}+\alpha_{n}\right)\left(\sigma +D_{o}\lambda_{j}+\xi_{o}-F^{\prime }\left(o_{0}\right)n_{0}\right)\left(\sigma +D_{n}\lambda_{j}\right)\left(\sigma +D_{c}\lambda_{j}+\xi_{c}+\lambda n_{0}\right)=0. \end{array}$$

yields all eigenvalues with negative real parts. Therefore, no spatial patterns emerge from this normoxic steady state.

In the hypoxic state, expanding the small perturbation $\left(\tilde{m},\tilde{o},\tilde{n},\tilde{c}\right)$ with respect to the Neumann spectral pair $\left(\lambda_{j},\phi_{j}\right)$ yields the following linear system

$$\begin{array}{c} \left(\begin{array}{cccc} \sigma +D_{m}\lambda_{j} & 0 & 0 & 0\\ \rho_{o} & \sigma +D_{o}\lambda_{j}+\xi_{o}-F^{\prime }\left(o_{0}\right)n_{0} & -F(o_{0}) & 0\\ 0 & 0 & \sigma +D_{n}\lambda_{j} & -\chi \left(c_{0}\right)n_{0}\lambda_{j}\\ -\eta & 0 & \lambda c_{0} & \sigma +D_{c}\lambda_{j}+\xi_{c}+\lambda n_{0} \end{array}\right)\left(\begin{array}{c} \tilde{m}\\ \tilde{o}\\ \tilde{n}\\ \tilde{c} \end{array}\right)=\left(\begin{array}{c} 0\\ 0\\ 0\\ 0 \end{array}\right). \end{array}$$

The dispersion relation reads as follows:

$$\begin{array}{cc} \; & \left(\sigma +D_{m}\lambda_{j}\right)\left(\sigma +D_{o}\lambda_{j}+\xi_{o}-F^{\prime }\left(o_{0}\right)n_{0}\right)\\ \; & \;\times \left[\left(\sigma +D_{n}\lambda_{j}\right)\left(\sigma +D_{c}\lambda_{j}+\xi_{c}+\lambda n_{0}\right)+\lambda c_{0}\chi \left(c_{0}\right)n_{0}\lambda_{j}\right]=0. \end{array}$$

We note that the first two factors have negative solutions $-D_{m}\lambda_{j}<0$ and $-(D_{o}\lambda_{j}+\xi_{o}-F^{\prime }\left(o_{0}\right)n_{0})<0$. All coefficients of the 2×2 *n*-*c* block’s characteristic polynomial are positive, and the Routh–Hurwitz criterion implies roots do not have positive real parts. Therefore, no spatial patterns emerge from this hypoxic steady state.

Together, the linear stability analysis shows that the qualitative linear stability mechanism for the tumor-oxygen-endothelial-TAF system is robust to replacing the linear supply with Michaelis–Menten kinetics.

## Appendix H. Periodic Boundary Condition

The choice of boundary condition directly influences the admissible eigenmodes and, therefore, the onset of pattern-forming instabilities. Specifically, for the same rectangular domain $U=\left[0,L_{a}\right]\times \left[0,L_{b}\right]$, periodic conditions are imposed as

$$\begin{array}{c} \phi_{j}\left(0,y\right)=\phi_{j}\left(L_{a},y\right),\;\phi_{j}\left(x,0\right)=\phi_{j}\left(x,L_{b}\right). \end{array}$$

Then the spectral pair consists of

$$\begin{array}{cc} \phi_{p,q} & =c_{p,q}\exp\left(\frac{2p\pi x}{L_{a}}i+\frac{2q\pi y}{L_{b}}i\right),\\ \lambda_{p,q} & ={\left(\frac{2p\pi }{L_{a}}\right)}^{2}+{\left(\frac{2q\pi }{L_{b}}\right)}^{2}, \end{array}$$

for $\left(p,q\right)\in Z^{2}$. We obtain the same dispersion relation as in the homogeneous Neumann boundary condition case, and the following patterns occur if, and only if:

$$\begin{array}{c} \frac{4\pi^{2}}{L_{b}^{2}}<{\bar{\lambda }}_{p,q}\Rightarrow L_{b}>\frac{2\pi }{\sqrt{{\bar{\lambda }}_{p,q}}}. \end{array}$$

Note that the condition $L_{b}>\frac{2\pi }{\sqrt{{\bar{\lambda }}_{p,q}}}$ is just the requirement that the first nontrivial Fourier mode (with p=0,q=1) lies in the unstable band.

## Appendix I. Derivation of the Threshold $\eta_{n}^{*}$

Below, we give a detailed derivation of the threshold $\eta_{n}^{*}$. The instability condition is

$$\begin{array}{c} a_{0}\left(\lambda \right)=D_{n}D_{c}\lambda^{2}+\left(D_{n}\xi_{c}-\eta_{n}\chi_{\mathrm{eff}}n_{0}\right)\lambda <0,\;\lambda >0. \end{array}$$

Dividing by λ gives

$$\begin{array}{c} \eta_{n}\chi_{\mathrm{eff}}n_{0}>D_{n}\xi_{c}+D_{n}D_{c}\lambda . \end{array}$$

In a finite-domain with Neumann conditions, the smallest nonzero eigenvalue is $\lambda_{\min}=\pi^{2}/\max{\left\{L_{a},L_{b}\right\}}^{2}$. Hence, instability requires

$$\begin{array}{c} \eta_{n}\chi_{\mathrm{eff}}n_{0}>A,\;A:=D_{n}\xi_{c}+\frac{\pi^{2}D_{n}D_{c}}{\max{\left\{L_{a},L_{b}\right\}}^{2}}. \end{array}$$

Since $\chi_{\mathrm{eff}}=\chi_{0}/\left(1+\alpha c_{0}\right)$ and $c_{0}=\eta_{n}n_{0}/\xi_{c}$, substituting yields

$$\begin{array}{c} \eta_{n}>\frac{A\xi_{c}}{\chi_{0}n_{0}\xi_{c}-A\alpha n_{0}}. \end{array}$$

Thus, the computational threshold is Equation (22), the algebraic form of which contains a denominator proportional to $\chi_{0}\xi_{c}-A\alpha$ (since the factor $n_{0}$ cancels). Hence, the expression is meaningful only when the denominator is positive:

$$\begin{array}{c} \chi_{0}\xi_{c}-A\alpha >0⟺\chi_{0}n_{0}\xi_{c}-A\alpha n_{0}>0. \end{array}$$

If this condition fails, the finite-domain threshold $\eta_{n}^{*}$ (as written) is not defined, and no Turing instability exists for any $\eta_{n}$ because the chemotactic gain cannot overcome the stabilizing terms.

To aid reproducibility, we briefly check this condition using the parameter set employed in the numerical experiments (used in Table 6 and the dispersion plots): $D_{n}=4.61\times 10^{-4}$, $D_{c}=0.12$, α=0.6, $\xi_{c}=0.002$, $\chi_{0}=0.0599$ (with the unit-square domain $L_{a}=L_{b}=5$ in nondimensional units). Using these values, one obtains

$$\begin{array}{c} A=D_{n}\xi_{c}+\frac{\pi^{2}D_{n}D_{c}}{5^{2}}\approx 2.2761\times 10^{-5},\;\chi_{0}\xi_{c}\approx 1.1980\times 10^{-4}, \end{array}$$

so

$$\begin{array}{c} \chi_{0}\xi_{c}-A\alpha \approx 1.0614\times 10^{-4}\;>\;0, \end{array}$$

and the denominator is positive.

## Appendix J. Limitations

For completeness, we document further limitations not detailed in the main text:

(i) Cell motility and invasion. Tumor cells are modeled with random motility via Brownian dynamics. Directed processes such as chemotaxis and haptotaxis, which are critical for invasion and metastasis, are omitted. Their integration would capture invasive behavior more accurately.
(ii) Biophysical forces and mechanics. Mechanical influences, including interstitial fluid pressure, extracellular matrix resistance, and cell–cell adhesion, are absent. These processes strongly influence vascular remodeling, drug extravasation, and tumor cell motility. Elevated interstitial pressure, for example, can restrict drug penetration and promote resistance niches near vessels [74]. Incorporating mechanical interaction modules, such as Lennard–Jones potentials, would enhance the biophysical realism.
(iii) Resistance evolution. Resistance mutations are modeled as neutral Poisson events, omitting directional biases, fitness costs, and environment-dependent mutation rates. More mechanistic models integrating genotype–phenotype coupling, stress-induced mutagenesis, and multi-omics-informed resistance will be needed.
(iv) Generalizability. While motivated by oncology, the framework has potential relevance for regenerative medicine and tissue engineering. Translating to these non-tumor contexts will require adapting cellular phenotypes, angiogenic stimuli, and perfusion targets.
